# Shared and Disease-Preferential Matrisome Programs in Fibrosing Interstitial Lung Disease: Evidence from Cross-Cohort and Within-Cohort Analysis

**DOI:** 10.3390/cimb48090951

**Published:** 2026-09-17

**Authors:** Xu Zhang, Yuan Wang, Pulin Che

**Affiliations:** Department of Anesthesiology and Perioperative Medicine, Division of Molecular and Translational Biomedicine, University of Alabama at Birmingham, Birmingham, AL 35294, USA; xzhang6@uab.edu (X.Z.); wangyuan19940401@gmail.com (Y.W.)

**Keywords:** matrisome, extracellular matrix remodeling, fibrosing interstitial lung disease, idiopathic pulmonary fibrosis, hypersensitivity pneumonitis, systemic sclerosis-associated interstitial lung disease, comparative transcriptomics, differential diagnosis

## Abstract

Idiopathic pulmonary fibrosis (IPF), hypersensitivity pneumonitis (HP), and systemic sclerosis-associated interstitial lung disease (SSc-ILD) share the same structural outcome: extracellular matrix (ECM) deposition. Whether this shared endpoint reflects shared or distinct molecular programs remains unresolved. Differential expression results from four GEO datasets representing IPF, HP, non-IPF ILD, and SSc-ILD were intersected with the 1027-gene human matrisome masterlist and classified by threshold-defined disease preference. All four diseases shared a 21-gene upregulated matrisome core, and the matrisome fraction rose with cross-disease sharing, from 4.3% of single-disease upregulated genes to 45.7% of genes upregulated in all four (significant Cochran–Armitage trend), and the enrichment persisted when any single cohort was removed. Apparent disease preference was largely attributable to unequal statistical power: of 29 genes measurable in all four datasets, 24 showed a concordant sub-threshold effect in at least one comparator disease. Category composition of the threshold-defined signatures did not differ significantly between diseases, and the exploratory pathway panel is reported within diseases only, because significance scales with cohort size. In a within-cohort comparison of IPF with chronic HP in GSE150910, with platform, cohort, and control population held constant, matrisome genes were over-represented among the 2732 genes separating the two diseases, and 205 matrisome genes distinguished them. Fibrosing ILDs share a cross-cohort ECM core, and the apparent disease preference of the remaining signatures largely reflects differences in statistical power between cohorts. Disease-preferential matrisome expression is nonetheless detectable when disease and study are not confounded, so the limitation lies in threshold-based cross-study intersection rather than in the matrisome compartment itself.

## 1. Introduction

Fibrosing interstitial lung diseases (ILDs) encompass a heterogeneous group of disorders that share a common endpoint, progressive scarring of the lung parenchyma, yet arise from different etiologies and require distinct therapeutic strategies [1,2,3,4,5,6]. Among these, four diseases present a persistent diagnostic challenge [3,7]: idiopathic pulmonary fibrosis (IPF), hypersensitivity pneumonitis (HP), non-IPF ILD (a heterogeneous group including non-specific interstitial pneumonia and other fibrosing ILDs), and systemic sclerosis associated ILD (SSc-ILD) [6,8,9,10]. These diseases frequently overlap in clinical presentation: similar patterns on high-resolution computed tomography (HRCT) [11,12], comparable pulmonary function decline [13,14], and indistinguishable symptoms of progressive dyspnea and cough [2]. Even with multidisciplinary team discussion, the current reference standard for ILD diagnosis [5,6], yields uneven inter-observer agreement across international expert centers: k = 0.60 for a first-choice diagnosis of IPF, but only k = 0.24 for HP and k = 0.25 for idiopathic NSIP [7]. Misdiagnosis carries direct therapeutic consequences: IPF responds to antifibrotic therapy (nintedanib, pirfenidone), whereas routine chronic immunosuppression is not recommended, and the combination of prednisone, azathioprine, and N-acetylcysteine was associated with increased mortality [5,15,16]. HP may reverse with antigen avoidance and immunomodulation [9,17,18], SSc-ILD may stabilize with mycophenolate mofetil [19], nintedanib [10], or tocilizumab [20], and several non-IPF ILD subtypes are conventionally managed with corticosteroids or other immunosuppression [6].

The pathology of all fibrosing ILDs is expressed in a single structural compartment: the extracellular matrix (ECM). The ECM is a dynamic signaling platform that instructs cell behavior, stores growth factors, transduces mechanical signals, and regulates the balance between tissue repair and fibrotic remodeling [21,22,23,24]. In fibrotic lung disease, the ECM undergoes quantitative and qualitative transformation: collagen accumulates, cross-linking enzymes stiffen the matrix, proteolytic balance shifts, and the normal architecture is replaced by scar tissue. These ECM changes are not only downstream consequences of cellular pathology; they also sustain fibroblast activation and epithelial dysfunction, forming feedback loops that perpetuate disease progression [23,24,25,26]. How the ECM is remodeled in each disease is therefore central to its pathogenesis.

The matrisome, defined as the complete ensemble of ECM structural proteins and ECM-associated factors, provides a systematic scheme for studying ECM biology at the transcriptomic level [27]. First cataloged by Naba and colleagues [27,28], the human matrisome comprises 1027 genes organized into six categories across two divisions: the core matrisome (collagens, ECM glycoproteins, and proteoglycans, which form the structural scaffold) and the matrisome-associated compartment (ECM regulators such as proteases and cross-linking enzymes, ECM-affiliated proteins, including C-type lectins, galectins, and annexins, and secreted factors, including growth factors and cytokines that signal through the matrix) [27,28,29]. The classification has been applied widely in cancer, where matrisome signatures predict survival and metastatic potential [30,31,32]. In fibrotic lung disease, however, the matrisome classification has been applied only to single diseases, and not systematically across multiple ILDs to separate what is shared from what distinguishes one disease from another.

Despite the central role of ECM remodeling in all fibrosing ILDs, our understanding of how each disease reshapes its matrisome remains incomplete. At the pathogenic level, it is unclear whether IPF, HP, and SSc-ILD activate the same ECM gene programs at different intensities or engage different molecular circuits. This distinction would help explain why diseases with similar histological endpoints respond to different therapies. At the diagnostic level, current biomarker efforts have focused on single ECM-derived molecules such as MMP7, KL-6, and SP-D, which, although prognostically informative within IPF [33,34,35], show variable specificity for discriminating between ILD subtypes when used in isolation. Whether the matrisome as a whole is organized differently between these diseases has not been examined systematically. At the therapeutic level, the two approved antifibrotics for IPF (nintedanib, pirfenidone) [15,16] target broadly expressed growth-factor signaling pathways [36]; consistent with this, nintedanib slows FVC decline across fibrosing ILDs irrespective of the underlying diagnosis [10,14,37]. A systematic, cross-disease characterization of the matrisome transcriptome is therefore needed to separate shared fibrotic biology from disease-preferential programs.

A recent systematic review and individual-participant meta-analysis compared ILD subtypes directly and externally validated signatures for IPF, HP, NSIP and SSc-ILD [38]; several genes highlighted here, including MMP7, COMP and COL17A1, also appear in that analysis. What has not been performed systematically is parallel annotation of the full matrisome transcriptome across multiple fibrosing ILDs. Previous cross-disease work has approached the question from four directions, none of which resolves disease-associated ECM composition in human ILD tissue. Time-resolved ECM proteomics has mapped matrix remodeling in fine temporal detail, but in a single murine injury model instead of across human diseases [39]. Transcriptomic network analyses spanning IPF, fHP, and CTD-ILD have identified both shared and disease-associated pathways, but their ECM finding was a single shared fibrosis module, and annotation was pathway-level only, leaving gene-level matrix composition unresolved [40]. Finally, multi-ILD transcriptomic and spatial atlases include the relevant comparators but have not applied systematic matrisome classification [41]. Single ECM genes such as MMP7, periostin, and collagen I/III have been studied extensively in IPF [33], but gene-by-gene approaches cannot capture systems-level reorganization of the ECM compartment or separate call-based disease-preferential patterns from pan-fibrotic background.

In this study, we performed a systematic cross-disease matrisome transcriptomic analysis across IPF, HP, non-IPF ILD, and SSc-ILD. Using pre-processed differential expression results from four publicly available GEO datasets, we applied the Naba matrisome classification to define shared UP-set intersections and threshold-defined, disease-preferential DEG-call signatures. Our data identify a matrisome core shared across all four arms, and a within-cohort comparison of IPF with chronic hypersensitivity pneumonitis in a single series shows that the matrisome is also enriched among the genes that separate one fibrosing ILD from another. The threshold-defined candidate list derived across studies does not recover that signal, which places the difficulty in cross-study thresholding rather than in the matrisome compartment itself.

## 2. Materials and Methods

### 2.1. Data Sources and Study Design

This study analyzed publicly available bulk transcriptomic datasets from the NCBI Gene Expression Omnibus (GEO). Four series were selected, one per disease arm: GSE150910 for IPF (103 IPF lungs against 103 non-diseased controls, RNA-seq), GSE184316 for fibrotic HP (multiple cores from 9 explant lungs against 6 unused donor lungs, RNA-seq), GSE213001 for non-IPF ILD (9 patients with end-stage fibrosing ILD other than IPF against 14 non-diseased control donors whose lungs were deemed unsuitable for transplantation, RNA-seq), and GSE76808 for SSc-ILD (14 arrays from 7 reported patients against 4 controls, Affymetrix HG-U133A 2.0 microarray). Sample numbers, platforms, and source publications for each series are given in Table 1. Series were selected on four criteria: human lung tissue; disease samples with a diagnosis established in the source study; availability of non-diseased control samples within the same series; and accessible processed expression matrices or published differential expression results.

### 2.2. Terminology

HP denotes hypersensitivity pneumonitis in general; where the source cohort definition requires the distinction, fibrotic HP (fHP) and chronic HP (CHP) are used as defined in the originating study. The HP arm of the present analysis comprises fibrotic HP explant cores from GSE184316.

### 2.3. Design Limitations and Confounding Structure

Because each disease arm derives from a different series, disease is confounded with study, platform, control population, tissue sampling method, and statistical power. The arms differ along every one of these dimensions: the IPF arm (103 versus 103) is roughly an order of magnitude larger than the HP (9 versus 6), non-IPF ILD (9 versus 14) and SSc-ILD (7 versus 4) arms; SSc-ILD was profiled on an Affymetrix microarray, whereas the other three arms are RNA-seq; the HP arm comprises multiple cores per explanted lung and the non-IPF ILD arm multiple regional samples per donor, so expression profiles outnumber participants in both; and control populations range from non-diseased donor lungs to lungs declined for transplantation. Comparisons between arms are therefore treated throughout as descriptive, and the disease-preference classification defined below is operational, not biological.

### 2.4. Data Preprocessing and Differential Expression Analysis

Differential expression was computed by us for all four arms from sample-level data obtained from GEO. The IPF arm (GSE150910) was analyzed with DESeq2 1.50.2 on gene-level counts. The HP arm (GSE184316) was analyzed with limma 3.66.0 on log2 (FPKM + 1), the non-IPF ILD arm (GSE213001) with limma-voom on raw counts, and the SSc-ILD arm (GSE76808) with limma on normalized array intensities. The HP and non-IPF ILD arms contribute repeated regional samples from identified donors, and both were fitted with donor as a random block using limma duplicateCorrelation, so within-subject correlation is modeled in the primary analysis of each. The SSc-ILD arm was fitted the same way on the GEO patient label, which reproduces its primary results, although those composite labels do not establish donor identity and repeated sampling in that arm is therefore not documented. The blocked fit is retained as primary because it is the conservative choice: 1107 differentially expressed genes against 1226 unblocked, and 21 matrisome genes in the shared core against 25. The IPF arm contributes one expression profile per participant, so the question does not arise there. For the IPF arm, log2 fold changes and Benjamini–Hochberg adjusted *p*-values were used. For the HP arm (GSE184316), fibrotic HP explant cores were compared with donor control lungs. For the non-IPF ILD arm, end-stage non-IPF fibrosing ILD samples of GSE213001 were compared with non-diseased donors of the same series (Table 1). For the SSc-ILD arm (GSE76808), SSc-ILD open lung biopsies were compared with normal lung on Affymetrix HG-U133A 2.0 arrays. For each dataset, genes with a Benjamini–Hochberg adjusted *p*-value below 0.05 and an absolute log2 fold change greater than or equal to 0.585 were classified as differentially expressed and assigned as upregulated or downregulated by the sign of the fold change relative to that study’s controls.

Within-cohort comparison of IPF and chronic hypersensitivity pneumonitis. GSE150910 contains IPF (*n* = 103), chronic HP (*n* = 82), and non-diseased control (*n* = 103) lungs within a single series, processed on one platform against one control population. To test the disease-preference assignment without the confounding that limits the four-arm design, gene-level counts were obtained from the GEO Supplementary File, and IPF was compared directly with chronic HP using DESeq2 (pydeseq2 0.5.4) with Wald tests, Benjamini–Hochberg correction, and the same DEG thresholds used throughout (adjusted *p* < 0.05, |log2FC| ≥ 0.585). The design formula was ~condition, with diagnosis as the only term. Genes with fewer than 10 counts in total were excluded, leaving 18,353 genes tested and 17,634 with an adjusted *p*-value. As a sensitivity analysis, the comparison was repeated with the design ~sex + age + ever_smoked + sample_type + plate + condition, fitted to the 183 of 185 samples with sex and age recorded (102 IPF, 81 chronic HP); smoking status was kept as a three-level factor so that the 15 such samples without a recorded status were retained, and the unadjusted model was refitted on the same samples so that the comparison isolates the adjustment (Table S10). Sequencing batch could not be included: no batch contains both IPF and chronic HP samples, so batch is completely separated by diagnosis and is not estimable. Plate carries the technical term instead of recruiting site, which two of five centers render partially separated by diagnosis. Enrichment of gene sets among genes separating the two diseases was assessed by Fisher’s exact test, against the remaining tested genes for the matrisome as a whole and against the remaining tested matrisome genes for the candidate list.

Donor blocking in the arms with repeated regional sampling. The HP arm contributes 36 expression profiles from 9 donors and 24 control profiles from 6 donors, four regions per donor; the non-IPF ILD arm contributes 26 profiles from 9 donors and 41 control profiles from 14 donors, one to five regions per donor. Each arm was refitted in R 4.4.1 with limma 3.60.4 twice on the identical gene set, once with donor as a random block (duplicateCorrelation with statmod 1.5.0) and once without, so that the difference between the two isolates the blocking (Table S11). The blocked refit reproduces the primary results in each arm. For the HP arm, the log2 fold changes agree to within 1.2 × 10^−14^, the moderated t-statistics to a median ratio of 1.007, and the raw *p*-values to a correlation of 0.99999; for the non-IPF ILD arm, the median t-ratio is 1.002, and the raw *p*-value correlation is 0.999. The unblocked refit reproduces neither: its t-statistics are a median of 1.30 times larger in the HP arm and 1.39 times larger in the non-IPF ILD arm, close to the variance inflation of sqrt (1 + (*n* − 1) × rho) expected at the estimated within-donor correlations of 0.206 and 0.379. The SSc-ILD arm was blocked in the same way. Its published results are reproduced to within 9 × 10^−14^ by a limma fit blocked on the GEO patient label, which supplies 12 blocks across the 18 arrays at a correlation of 0.396 within label, and are not reproduced without it (median t ratio 1.06, and t statistics differing by up to 1.28 in absolute terms; Table S11). Because the composite labels, such as patient31_and_08, do not establish donor identity, that block is an assumption about the series rather than a documented design, and the shared core was therefore also recomputed with the SSc-ILD arm unblocked. Sharing degree and the shared matrisome core were then recomputed from the blocked refits of the HP and non-IPF ILD arms, with the IPF and SSc-ILD arms held unchanged.

### 2.5. Threshold-Defined Disease-Preference Classification

**Figure 1:**
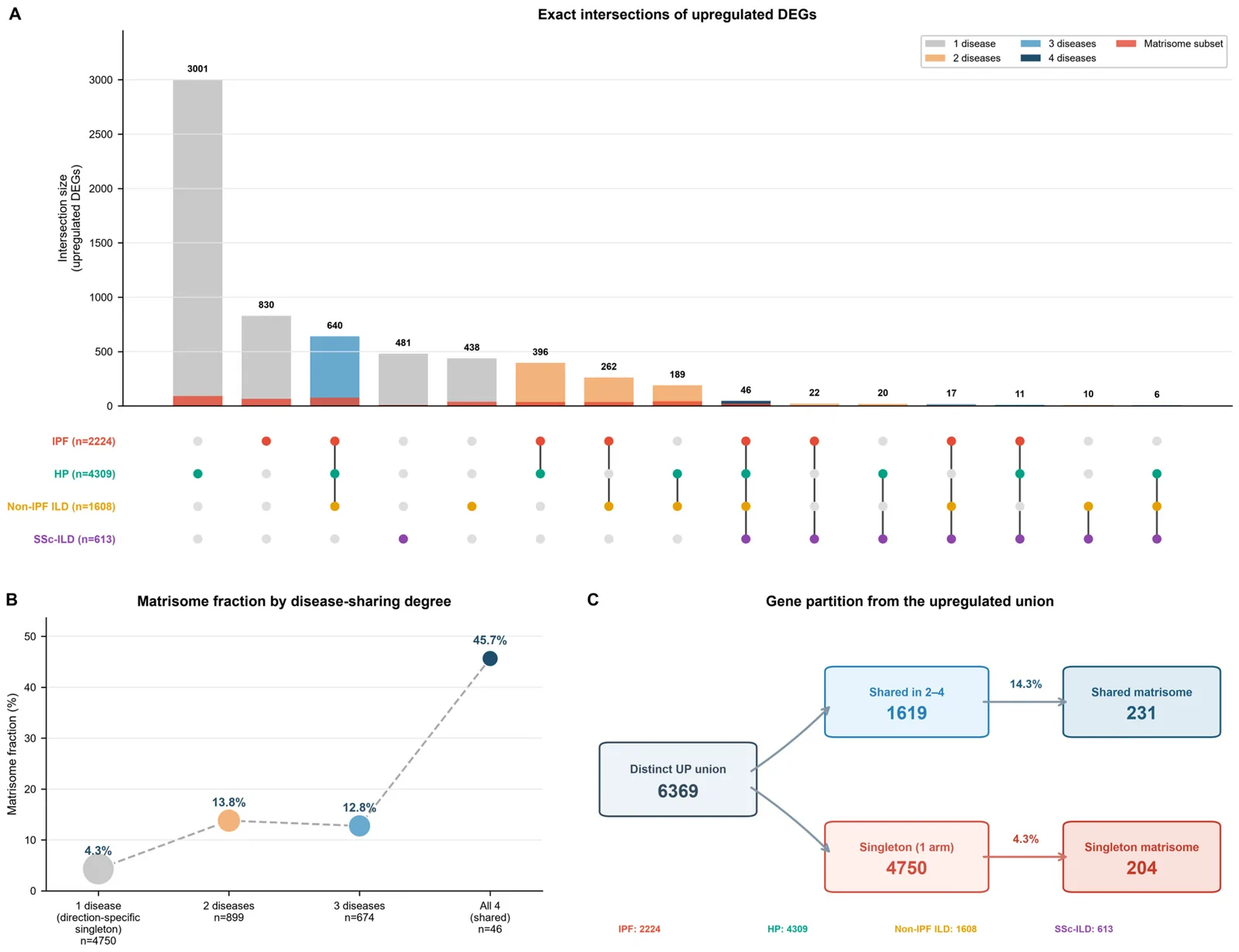
**Cross-disease transcriptomic architecture.** (**A**) UpSet plot showing exact intersections of upregulated DEGs across IPF, HP, non-IPF ILD, and SSc-ILD. Each column represents an UP-set intersection pattern; bar height indicates gene count, and the red overlay indicates matrisome content. HP-only genes dominate (3001), while the all-four intersection contains 46 genes, including 21 matrisome genes. (**B**) Observed matrisome fractions by UP-set sharing degree: 4.3% for genes present in one UP set, 13.8% for two, 12.8% for three, and 45.7% for all four. Bubble size reflects total gene count, and the dashed line joins the observed fractions. (**C**) Partition of the 6369-gene upregulated union into genes shared by two to four UP sets (1619, blue) and genes present in exactly one UP set (4750, red), with 231 and 204 matrisome genes, respectively; the percentage above each arrow is the matrisome fraction of that set. Direction-specific UP-set singleton status is not equivalent to the threshold-defined disease-preference rule used in later figures.

Two related but distinct classifications were used. For the direction-specific intersections in Figure 1, a gene was counted as present in exactly one upregulated-gene set when it was upregulated in only one arm, even if it was downregulated in another arm. For the threshold-defined disease-preferential signatures in Section 3.3, Section 3.4 and Section 3.5 and Figure S1, a gene had to meet DEG criteria in exactly one disease arm and fail DEG criteria in all three comparator arms, regardless of direction; genes meeting DEG criteria in multiple arms with opposing directions were classified as divergent and excluded from the threshold-defined signatures. A comparator arm in which a gene was not measured provides no evidence about that gene, and a non-significant comparator result accompanied by a concordant effect size is consistent with the same regulation detected at lower power, not with absence of regulation. For the 101 threshold-defined IPF-dominant matrisome genes, evidence classes were assigned from comparator coverage and effect sizes: coverage-limited genes lacked a result in at least one comparator arm; among genes measurable in all four datasets, power-sensitive genes had at least one comparator whose effect was concordant in direction with the IPF effect at |log2FC| greater than or equal to 0.3, and IPF-dominant genes had no such comparator. Sensitivity analyses raised the IPF effect threshold to |log2FC| greater than or equal to 0.8 and 1.0. As a partial replication, GSE184316, which includes IPF and UIP explant samples alongside the fibrotic HP samples constituting the HP arm, was used to test the same threshold and direction in an IPF-versus-control contrast with the same correction for correlation within lungs. Only 47 of the 101 genes were evaluable in that contrast.

### 2.6. Matrisome Annotation

Threshold-defined disease-preferential DEGs were intersected with the human matrisome masterlist (1027 genes; 274 core matrisome and 753 matrisome-associated), as defined by Naba et al. [27,28] and revised in the annotation subsequently distributed through the Matrisome Project portal [29]. Each matrisome gene was classified into its corresponding matrisome division and category (collagens, ECM glycoproteins, proteoglycans, ECM regulators, ECM-affiliated proteins, or secreted factors). Matrisome fractions were calculated within the corresponding direction-specific or threshold-defined disease-preferential DEG set, as specified for each analysis.

### 2.7. Functional and Pathway Analysis

Functional labels for IPF-dominant matrisome genes were used descriptively and were not treated as co-expression modules or as results of over-representation testing. For pathway comparison, 17 author-curated gene sets covering signaling, immune, and ECM programs relevant to fibrosing ILD were used as an exploratory panel. These sets were curated locally, informed by canonical pathway definitions, and are not extractions from a versioned database. Table S3a reports the complete membership of all 17 sets (248 gene-set memberships across 216 distinct genes) together with the nearest public counterpart and its stable identifier; 16 sets map to a KEGG, Reactome, or MSigDB Hallmark counterpart, whereas one set, fibroblast activation, has no canonical equivalent and is declared as author-curated. Upregulated and downregulated genes were combined in the over-representation test, so an enriched result indicates pathway involvement and not pathway activation or direction. For each disease, the DEG list limited to a common universe of 9239 gene symbols measurable in all four datasets (IPF 1430; HP 1772; non-IPF ILD 1300; SSc-ILD 904 genes) was tested for enrichment by one-sided Fisher’s exact test, with the pathway sets likewise limited to that universe, with Benjamini–Hochberg correction within each disease and FDR < 0.05 considered enriched. Because Fisher’s exact *p* scales with DEG list size and the arms differ substantially in sample size, enrichment results are reported within each disease only and are not compared between diseases; no cross-disease discrimination index was computed. For cross-disease comparison, the distribution of threshold-defined disease-preferential matrisome genes across the six Naba categories was computed for each disease and tested formally, as described below.

### 2.8. Statistical Analysis

Statistical testing was performed in Python 3.12.13 with pandas 3.0.1 and NumPy 2.3.5; figures were generated in Python 3.13.15 with matplotlib 3.11.1. Differential expression significance followed Benjamini–Hochberg correction (adjusted *p* < 0.05). Matrisome enrichment by cross-disease sharing degree was assessed against a background universe of 6369 distinct upregulated DEGs, of which 435 (6.83%) are matrisome members, using the hypergeometric test with odds ratios and Woolf 95% confidence intervals, and a Cochran–Armitage test was used for trend across sharing degree. Matrisome category composition was compared between diseases by chi-square with Monte-Carlo permutation and by pairwise contingency tests, with Wilson binomial 95% confidence intervals reported for each category proportion. Robustness of the sharing-degree enrichment was assessed by leave-one-cohort-out analysis: each disease arm was removed in turn, sharing degree was recomputed across the three retained arms, and both the enrichment odds ratio at maximum sharing degree and the Cochran–Armitage trend were re-estimated against the correspondingly recomputed background universe (Table S7; Figure S2).

## 3. Results

### 3.1. Distribution of Differentially Expressed Genes Across the Four Diseases

Differential expression analysis across the four fibrosing ILD datasets (Table 1) identified 6369 distinct upregulated genes and 2630 distinct downregulated genes. Total DEG counts for each arm, before intersection, are reported in Table S1. UpSet intersection analysis revealed that the majority of upregulated DEGs were present in one upregulated-gene set, with progressively fewer genes shared across two diseases, three diseases, or all four diseases (Figure 1A). HP contributed the largest single-disease upregulated pool, consistent with its broad immune-inflammatory transcriptional program. The all-four-diseases intersection (46 genes) was small but had the highest observed matrisome fraction.

The matrisome fraction increased with the degree of cross-disease sharing (Figure 1B). Genes upregulated in only one disease showed 4.3% matrisome content (204 of 4750), compared with 13.8% for two diseases (124 of 899), 12.8% for three diseases (86 of 674), and 45.7% for all four diseases (21 of 46). Against the background universe of 6369 upregulated DEGs (435 matrisome, 6.83%), matrisome representation was depleted among single-disease genes and enriched among shared genes: odds ratio 0.27 for a single disease (95% CI 0.22–0.33, *p* = 1.4 × 10^−37^), 2.65 for two diseases (2.13–3.31, *p* = 5.0 × 10^−16^), 2.24 for three (1.74–2.88, *p* = 3.5 × 10^−9^), and 11.99 for all four (6.65–21.60, *p* = 2.9 × 10^−13^), a significant increasing trend across sharing degree (Cochran–Armitage Z = 13.93, *p* = 4.4 × 10^−44^; Table S5). The rise is not stepwise: enrichment is comparable at sharing degrees two and three and increases sharply only at four. Of 435 upregulated matrisome DEGs across the four disease sets, 231 (53%) were shared by two or more diseases and 204 (47%) were direction-specific singletons (Figure 1C). The enrichment was not produced by any single cohort. In leave-one-cohort-out analysis, in which each arm was removed in turn and sharing degree recomputed across the three retained arms, matrisome genes remained over-represented among genes upregulated in every retained arm in all four analyses, and the increase with sharing degree persisted in each. The odds ratio was smallest when the SSc-ILD arm was removed, because that arm has the narrowest DEG list and is the binding constraint on the four-way intersection; the direction and significance of the effect were unchanged (odds ratios 2.48–11.78, all *p* < 1.1 × 10^−11^; Cochran–Armitage trend Z 7.78–14.82, all *p* < 7.6 × 10^−15^; Table S7; Figure S2).

The enrichment already accounts for within-subject correlation. The HP and non-IPF ILD arms carry repeated regional samples from identified donors, and both primary analyses fitted donor as a random block, with estimated within-donor correlations of 0.21 and 0.38. The SSc-ILD arm was fitted the same way on the composite GEO patient label (correlation 0.40), which reproduces its published results but does not itself establish donor identity. Refitting each arm with and without that block confirms it: the blocked refit reproduces the primary results, whereas the unblocked refit inflates its t-statistics by a median factor of 1.06 to 1.39 depending on the arm (Table S11). The shared core and the threshold-defined candidate list therefore already carry the correlation structure, and recomputing sharing degree from the blocked refit returns the same 46 upregulated genes and the same 21 matrisome members, with an odds ratio of 12.18 (6.76–21.93) at sharing degree four and a Cochran–Armitage trend of Z = 14.70; the odds ratio differs from 11.99 only because the background universe of upregulated DEGs moves from 6369 to 6663 as the two refits change those arms’ DEG lists. Recomputing the candidate list the same way returns 96 of its 101 genes; the five that differ (C1QTNF8, CLEC14A, IL13, ITIH1 and MUC3A) do so because the refit measures them in the HP arm where the original table did not, or because they sit at the significance threshold, not because of the correlation structure. The shared core does depend on the SSc-ILD block: recomputing with that arm unblocked, and all else as published, gives 58 shared upregulated genes, of which 25 are matrisome members, odds ratio 10.92 (6.43–18.53, *p* = 7.7 × 10^−15^). Five matrisome genes enter (COL1A2, COL5A2, MFAP2, PAMR1 and SFRP4), at adjusted *p* 0.052 to 0.073 blocked and 0.028 to 0.043 unblocked, and ASPN leaves, crossing the other way at 0.0498 blocked and 0.0514 unblocked. The enrichment is therefore robust to the assumption, while the membership of the core is not, and the 21-gene core reported here is the blocked result.

### 3.2. Exploratory Pathway Panel

**Figure 2:**
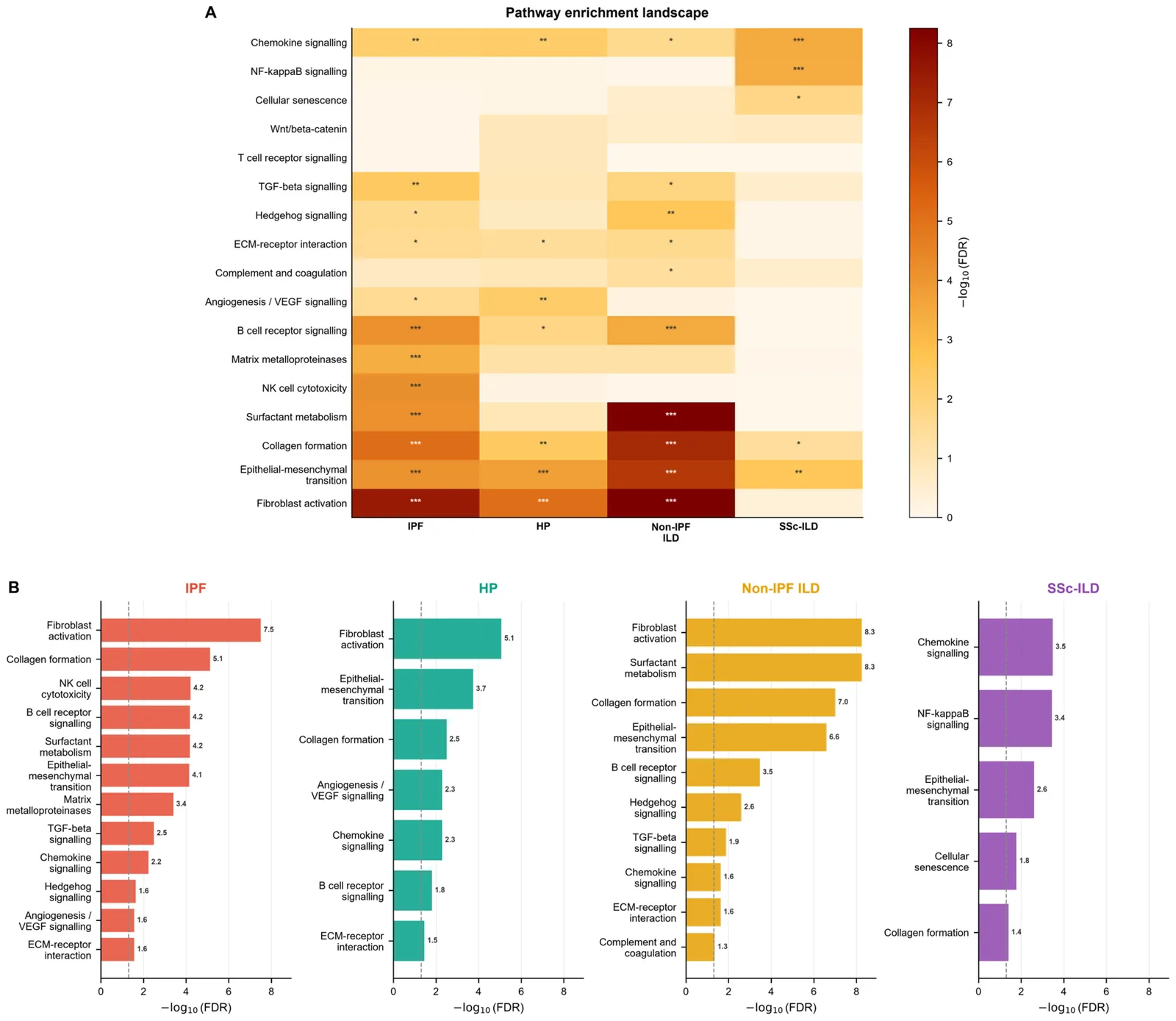
**Exploratory 17-program pathway panel across four fibrosing ILDs.** (**A**) Heatmap of -log10 (FDR) values, with significance markers (* FDR < 0.05, ** <0.01, *** <0.001). Fifteen programs were significant in at least one disease; Wnt/β-catenin and T-cell receptor signaling were not significant in any arm. (**B**) Ranked significant programs for each disease; the dashed line marks FDR = 0.05. Programs are ranked within each disease only; because significance scales with DEG list size, ranks are not comparable between diseases.

To determine whether divergence among fibrosing ILDs extends beyond the matrisome, we tested the full DEG list of each disease against 17 curated pathway gene sets (Figure 2). Fifteen of the seventeen pathways reached FDR < 0.05 in at least one disease; Wnt/β-catenin and T-cell receptor signaling did not.

Six programs were significant in at least three diseases (Figure 2A,B): chemokine signaling, collagen formation, and epithelial–mesenchymal transition in all four; fibroblast activation, ECM-receptor interaction, and B-cell receptor signaling in three of four. The remaining significant programs showed narrower patterns (Figure 2A,B). Surfactant metabolism was strongest in non-IPF ILD but also reached significance in IPF. Hedgehog signaling was significant in non-IPF ILD and IPF. NF-κB signaling and cellular senescence were significant only in SSc-ILD. Matrix metalloproteinases and NK-cell cytotoxicity were significant only in IPF.

The programs reaching significance within each disease are listed in Figure 2B. IPF was led by fibroblast activation and collagen formation, followed by NK-cell cytotoxicity, B-cell receptor signaling, and surfactant metabolism. HP was led by fibroblast activation, epithelial–mesenchymal transition, and collagen formation. Non-IPF ILD was led jointly by fibroblast activation and surfactant metabolism. SSc-ILD was led by chemokine signaling and NF-κB signaling. The number of significant programs differed substantially: 12 for IPF, 10 for non-IPF ILD, 7 for HP, and 5 for SSc-ILD. This ordering tracks cohort size and DEG list size and is therefore not interpretable as a biological difference between diseases.

### 3.3. Matrisome Genes Shared by All Four Diseases

**Figure 3:**
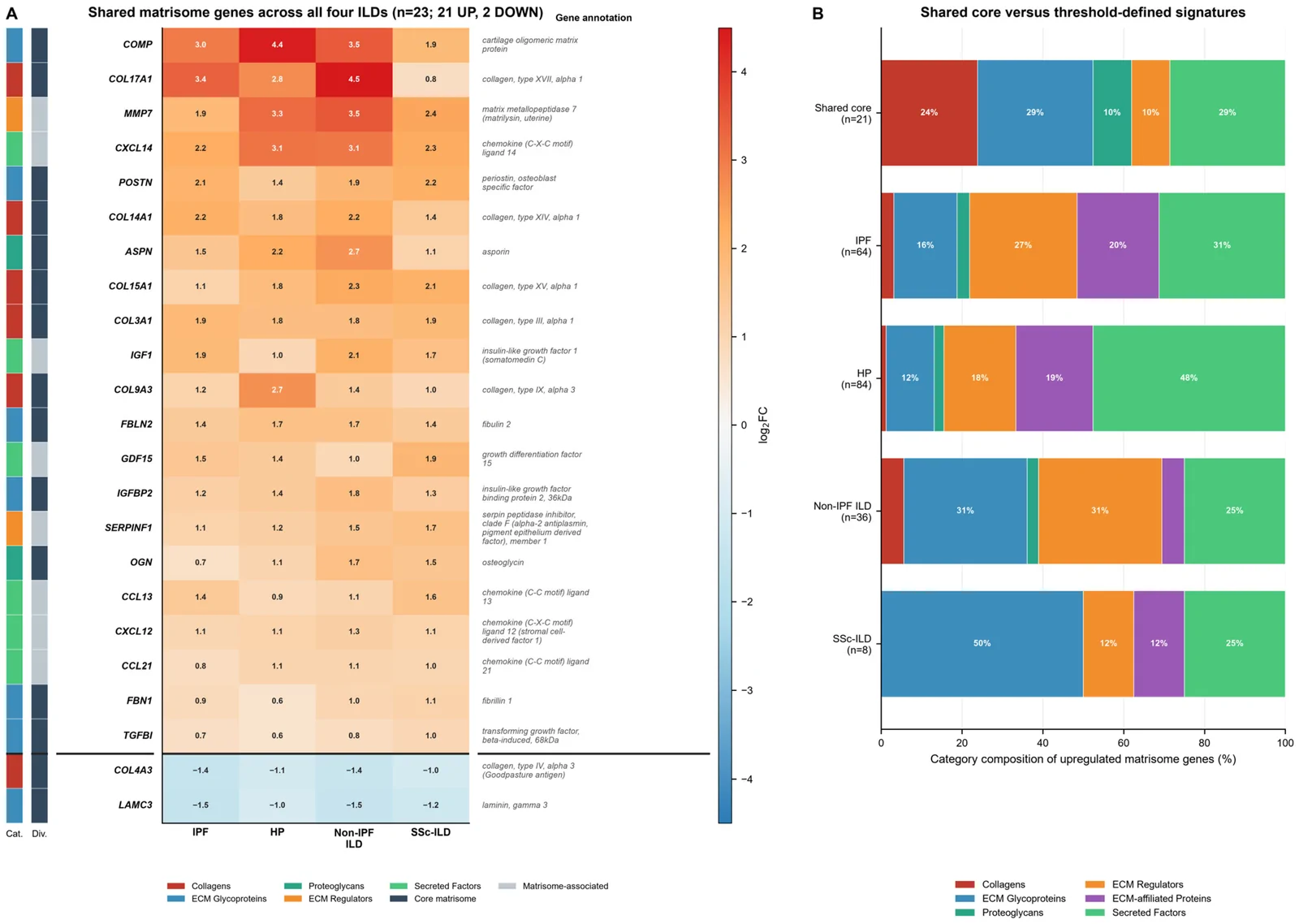
**Matrisome genes shared across all four ILDs.** (**A**) Annotated heatmap of 23 concordant matrisome genes: 21 upregulated and 2 downregulated. Color intensity and cell labels indicate log2 fold change; sidebars show matrisome category and division. (**B**) Category composition of the 21-gene upregulated shared core compared with upregulated threshold-defined signatures (IPF *n* = 64, HP *n* = 84, non-IPF ILD *n* = 36, SSc-ILD *n* = 8). The shared core contains 24% collagens, 29% ECM glycoproteins, 10% proteoglycans, 10% ECM regulators, and 29% secreted factors.

Twenty-one matrisome genes were upregulated in all four fibrosing ILDs, forming the shared upregulated matrisome core (Figure 3A). The core comprised five collagens (COL3A1, COL9A3, COL14A1, COL15A1, and COL17A1), six ECM glycoproteins (COMP, POSTN, FBLN2, IGFBP2, FBN1, and TGFBI), two proteoglycans (ASPN and OGN), two ECM regulators (MMP7 and SERPINF1), and six secreted factors (CXCL14, IGF1, GDF15, CCL13, CXCL12, and CCL21). Two additional matrisome genes, COL4A3 and LAMC3, were downregulated in all four ILDs, yielding a 23-gene concordant shared signature when both directions are considered.

The heatmap showed concordant directionality with variable magnitude across all 23 shared genes (Figure 3A). COMP showed the largest fold change in HP (log2FC = 4.4) but was consistently upregulated in all four diseases. COL17A1 peaked in non-IPF ILD and IPF, and MMP7 was upregulated across all four diseases. Figure 3B compares the category composition of the 21-gene shared core with the threshold-defined signatures.

### 3.4. Descriptive Category Composition of Threshold-Defined Signatures

The four fibrosing ILDs showed category profiles that differed descriptively but not significantly in their threshold-defined matrisome signatures (Figure S3, Table S6a,b). IPF was led by secreted factors and ECM regulators. HP was dominated by secreted factors. Non-IPF ILD showed equal contributions from ECM regulators and ECM glycoproteins. SSc-ILD was dominated by ECM glycoproteins.

**Figure 4:**
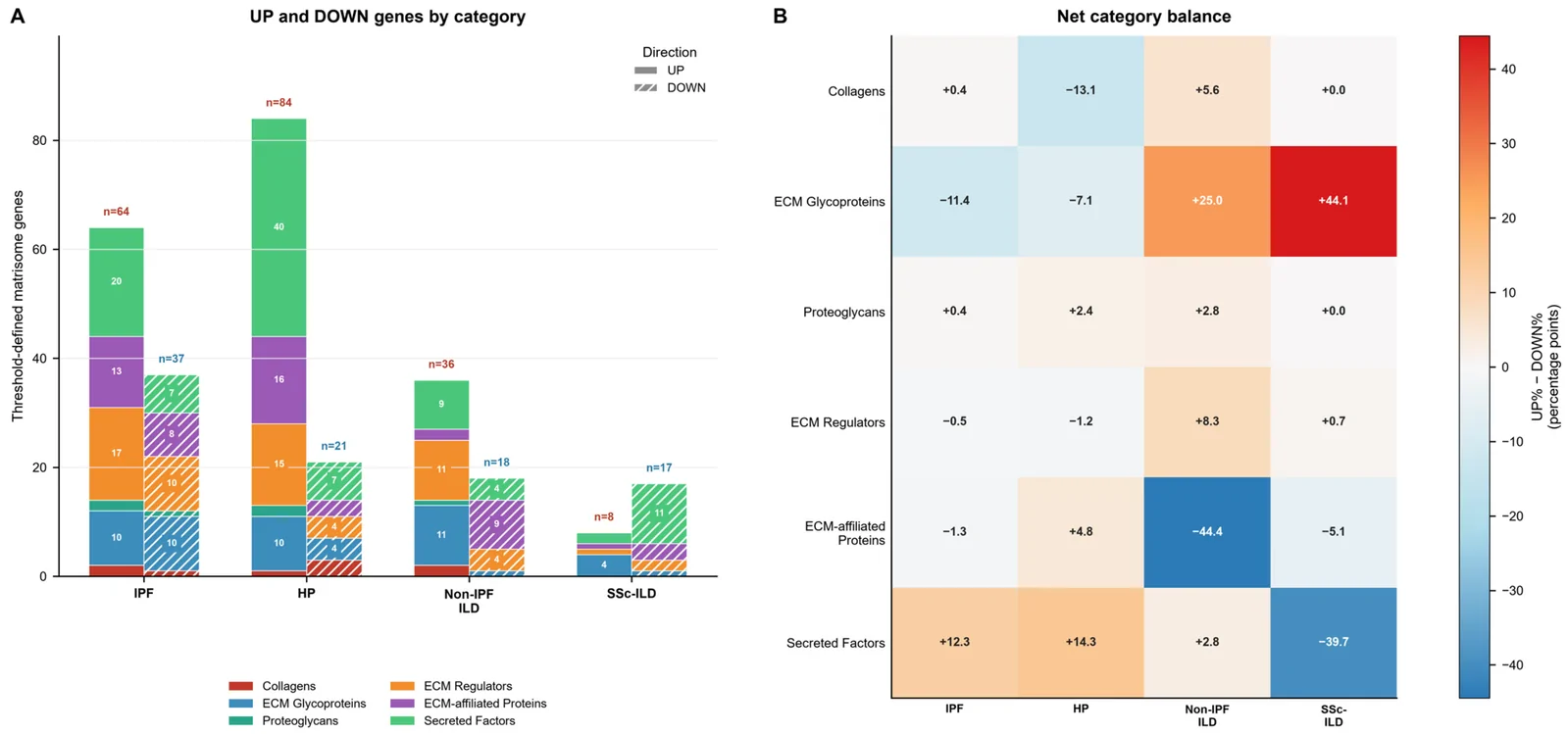
**Direction and category balance of the threshold-defined matrisome signatures.** (**A**) Upregulated and downregulated genes by category for each disease. (**B**) Net category balance heatmap showing the difference between upregulated and downregulated category percentages, in percentage points, for each disease. Category composition, with exact counts and Wilson binomial 95% confidence intervals, is shown in Figure S3; composition of the upregulated signatures did not differ significantly between diseases (chi-square = 22.93, df = 15, *p* = 0.086 and 20,000-permutation *p* = 0.088, computed from the counts in Table S6a; pairwise *p* = 0.25, 0.34 and 0.44 against IPF, Table S6b).

Upregulated and downregulated counts by category are shown in Figure 4A. IPF had 64 upregulated and 37 downregulated threshold-defined matrisome genes (Table 2). HP was gain-dominant (84 UP, 21 DOWN), non-IPF ILD had 36 UP and 18 DOWN, and SSc-ILD had 8 UP and 17 DOWN. The net category balance heatmap (Figure 4B) shows the difference between upregulated and downregulated category percentages, in percentage points, for each disease. Twelve of the twenty-four cells in the category-by-disease table had expected counts below five, so these distributions are too sparse to support inference; exact counts with Wilson binomial confidence intervals are given in Table S6a.

### 3.5. A Threshold-Defined IPF-Dominant Matrisome Candidate List

**Figure 5:**
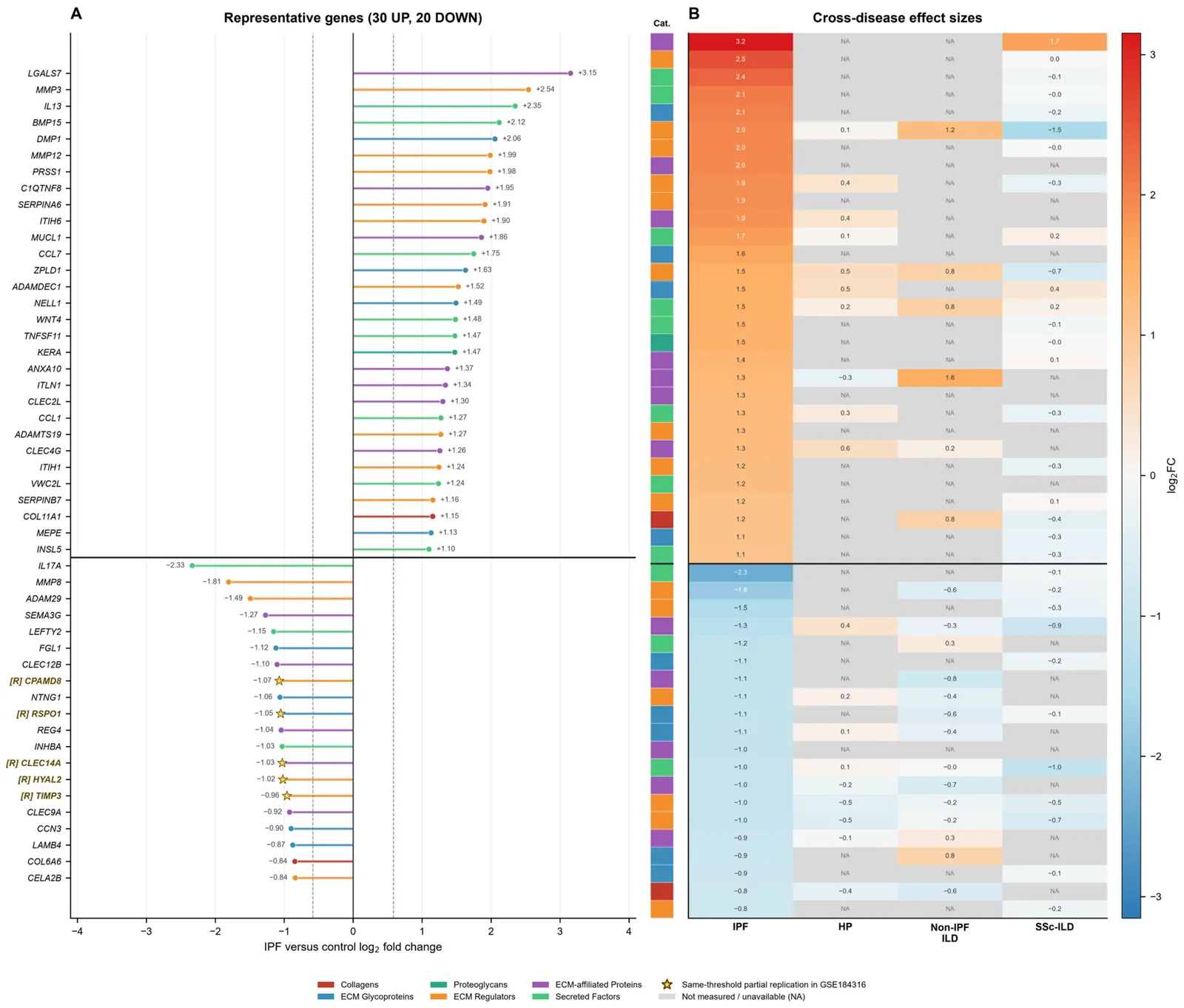
**Representative genes from the threshold-defined IPF-dominant matrisome candidate list.** (**A**) The 30 highest-effect upregulated and 20 strongest downregulated genes from the 101-gene signature, organized by IPF log2 fold change and matrisome category. Dashed lines mark |log2FC| = 0.585. Gold stars and gold [R]-prefixed gene labels mark five genes in this displayed subset with the same-threshold partial replication in the GSE184316 IPF contrast. (**B**) Cross-disease log2 fold changes for the same 50 genes. The complete 101-gene signature (64 UP, 37 DOWN), including all eight partially replicated genes, is shown in Figure S1 and Table S2. Gray cells in the heatmaps indicate unavailable measurements, not zero change.

Having identified the shared matrisome core, we next asked which matrisome genes met DEG criteria in IPF alone and failed to reach significance in any of the three comparator arms, regardless of direction. This threshold-based definition yielded 101 genes. Because it rests on significance calls, not on formal comparison of effect sizes between diseases, the resulting list is operational: we present it as a candidate list, not a disease-specific signature. Figure 5 displays the 30 highest-effect upregulated and 20 strongest downregulated genes, while Figure S1 shows the complete signature.

The 101-gene signature comprised 64 upregulated and 37 downregulated transcripts (Figure 5 and Figure S1; Table 3 and Table 4). Among the upregulated genes, secreted factors and ECM regulators were the most frequent categories, followed by ECM-affiliated proteins, ECM glycoproteins, collagens, and proteoglycans. The downregulated genes were led by ECM glycoproteins and ECM regulators, followed by ECM-affiliated proteins and secreted factors. Cross-disease effect sizes place important limits on this assignment (Figure 5B and Figure S1B). Only 29 of the 101 genes were measurable in all four datasets; the remaining 72 lacked a result in at least one comparator arm and are coverage-limited. Of 303 possible comparator measurements, 165 (54.5%) were available, and 61 of those (37.0%) showed a concordant direction with |log2FC| ≥ 0.3, consistent with the same regulation detected at lower power. Among the 29 genes measurable in all four datasets, only 5 showed no concordant comparator signal, 24 showed at least one, and 21 had a comparator effect at least half the magnitude of the IPF effect (Table S4). The list therefore partitions into 5 IPF-dominant genes, 24 power-sensitive genes, and 72 coverage-limited genes. A missing comparator measurement is not evidence that the gene is unregulated in that disease.

ECM regulators were prominent in both directions within the IPF signature, with gains of MMP3 and MMP12 and losses of TIMP3 and HYAL2. This category-level prominence, however, was not unique to IPF: ECM regulators also accounted for 31% of the non-IPF ILD upregulated signature. Moreover, MMP3 was coverage-limited, and MMP12 and TIMP3 were classified as power-sensitive.

### 3.6. Within-Cohort Comparison of IPF and Chronic Hypersensitivity Pneumonitis

**Figure 6:**
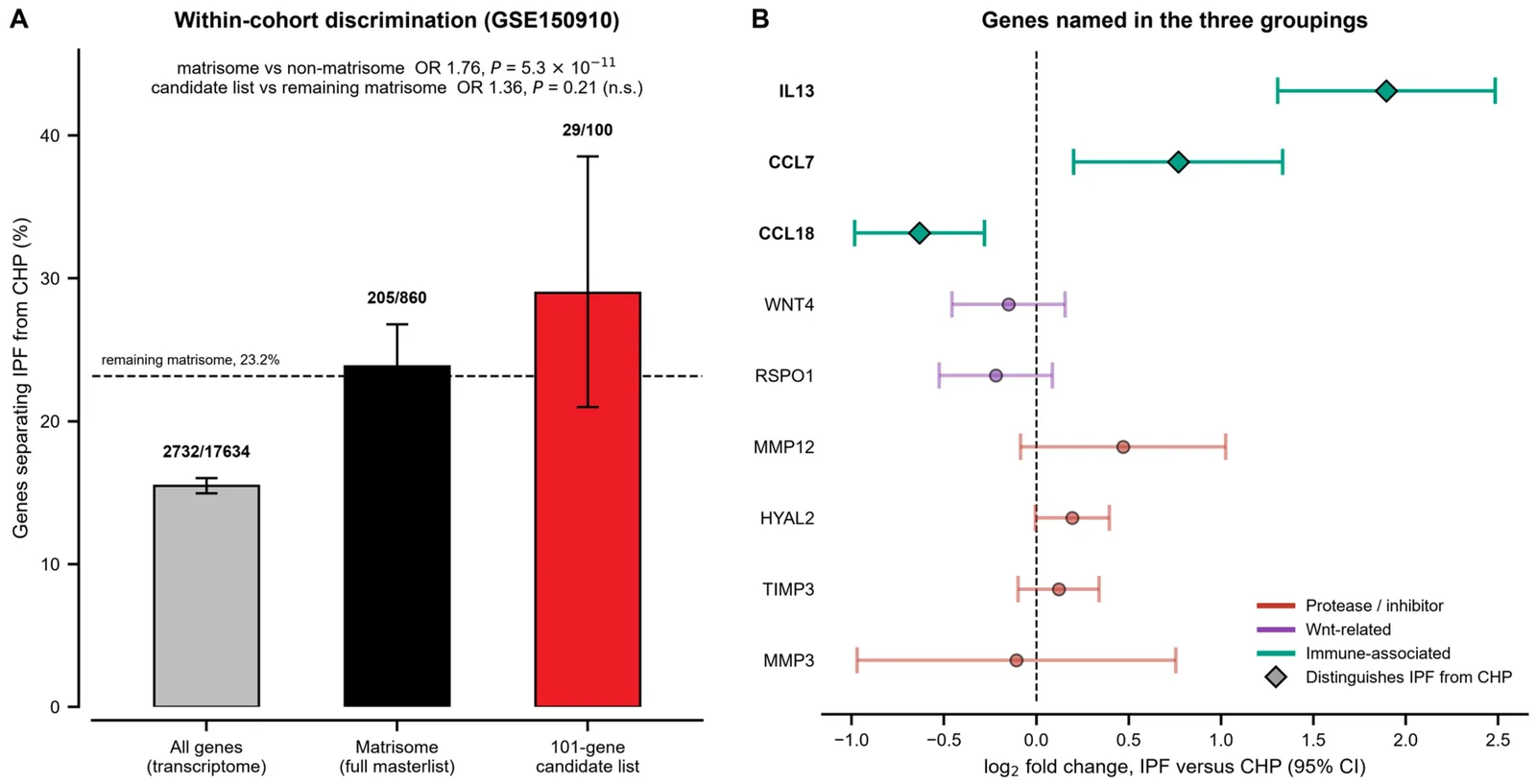
**Within-cohort comparison of IPF and chronic hypersensitivity pneumonitis in GSE150910 (103 IPF versus 82 chronic HP, one platform, one control population).** (**A**) Percentage of genes separating the two diseases, with Wilson binomial 95% confidence intervals, for the whole transcriptome, the full matrisome masterlist, and the 101-gene threshold-defined candidate list; all three count genes distinguishing the two diseases in either direction, and the dashed line marks the rate in the remaining tested matrisome, the background against which the candidate list is tested. Matrisome genes are over-represented among distinguishing genes, whereas the candidate list is not distinguishable from the rest of the matrisome. Table S12 reports every contingency test with the definition applied on each side. (**B**) Effect sizes with 95% confidence intervals for the genes named in the three descriptive groupings; estimates are from the unadjusted model, and filled diamonds distinguish IPF from chronic HP at adjusted *p* < 0.05 and |log2FC| ≥ 0.585, while intervals crossing zero do not; of the three immune-associated genes, CCL7 no longer meets these criteria under covariate adjustment (Table S10). Full per-gene results are in Table S8.

The four-arm design cannot separate disease from study. GSE150910 permits the comparison that can, because it contains IPF, chronic HP, and control lungs in one series on one platform. Comparing IPF directly with chronic HP within that cohort, 2732 of 17,634 tested genes (15.5%) met DEG criteria, so the two diseases are transcriptionally distinguishable in general (Figure 6A).

Matrisome genes were over-represented among those distinguishing genes: 205 of 860 matrisome genes tested (23.8%) separated IPF from chronic HP, compared with 15.1% of non-matrisome genes (odds ratio 1.76, *p* = 5.3 × 10^−11^), with 141 higher in IPF and 64 higher in chronic HP. Disease-preferential matrisome expression is therefore detectable once disease is no longer confounded with study, platform, and control population; the 205 genes are listed in Table S9.

The threshold-defined candidate list did not capture it. Of the 101 genes, 100 were testable in this cohort, and all 100 fall within the 860 matrisome genes tested, so the comparison that matters is with the remaining 760. In total, 29 of the 100 (29.0%) separated IPF from chronic HP, against 176 of 760 (23.2%) in the rest of the tested matrisome: odds ratio 1.36 (95% CI 0.85–2.15, *p* = 0.21). Applying the direction-concordant definition of IPF preference to both sides gives 20 of 100 (20.0%) against 115 of 746 (15.4%), odds ratio 1.37 (95% CI 0.81–2.33, *p* = 0.25), the comparison set falling from 760 to 746 because 14 of those genes have no IPF-arm fold change and cannot be scored for concordance; against the rest of the transcriptome under the same any-direction criterion the list is enriched, 29 of 100 against 2703 of 17,534 (odds ratio 2.24, 95% CI 1.45–3.46, *p* = 7.0 × 10^−4^; Figure 6A, Tables S8 and S12). The result was similar in every evidence class: under the direction-concordant definition, 2 of 5 IPF-dominant, 4 of 24 power-sensitive, and 14 of 71 coverage-limited genes were confirmed; the coverage-limited class fell from 72 to 71 because REG4 is not testable in this cohort, and 3 of 5, 7 of 24, and 19 of 71 distinguished the two diseases in either direction. The list is therefore enriched for discriminating genes relative to the transcriptome, as any matrisome-derived list would be, but not detectably more than the matrisome from which it was drawn, and the limitation lies in the threshold-based intersection rather than in the matrisome compartment.

Individual assignments behaved accordingly (Figure 6B). Of the genes highlighted in the Discussion, IL13 and CCL7 were confirmed as IPF-preferential, whereas MMP3, MMP12, TIMP3, HYAL2, WNT4 and RSPO1 did not distinguish the two diseases. CCL18 was significantly higher in chronic HP than in IPF, the reverse of the direction implied by its original threshold-defined assignment. Covariate adjustment left the comparison substantially unchanged. Effect sizes agreed closely between the adjusted and unadjusted models (Spearman r = 0.978, direction agreement 96.7%), 92.1% of the genes called under the unadjusted model, among those with an adjusted *p* value in both models, continued to meet the same DEG criteria under the adjusted one, and the matrisome enrichment persisted: 197 of 885 matrisome genes (22.3%) against 15.6% of non-matrisome genes, odds ratio 1.54 (95% CI 1.31–1.82, *p* = 5.5 × 10^−7^), against odds ratio 1.69 in the unadjusted model on the same samples (Table S10). Among the individual genes, IL13 (log2FC 1.85, adjusted *p* = 1.0 × 10^−8^) and CCL18 (log2FC −0.72, adjusted *p* = 2.2 × 10^−4^) kept their assignments under adjustment, whereas CCL7 fell below both thresholds (log2FC 0.51, adjusted *p* = 0.11, against log2FC 0.74 and adjusted *p* = 0.024 unadjusted on the same samples).

## 4. Discussion

This study presents a systematic cross-disease comparison of matrisome gene expression across four clinically confusable fibrosing ILDs using the Naba matrisome annotation, combined with an exploratory 17-program pathway panel. Two findings emerge. First, the matrisome carries a shared fibrotic core: 45.7% of the genes upregulated in all four cohorts are matrisome members, compared with 6.8% of the background universe (odds ratio 11.99), the enrichment increases significantly with the degree of cross-disease sharing, and it persists when any single cohort is removed. Second, disease-preferential matrisome expression is detectable once disease is no longer confounded with study, platform, and control population: within GSE150910, matrisome genes were over-represented among the genes separating IPF from chronic HP (odds ratio 1.76, *p* = 5.3 × 10^−11^), and 205 matrisome genes distinguished the two diseases. What the data do not support is the threshold-defined route to that second signal. Under a threshold-based DEG-call definition, the 101-gene IPF-dominant matrisome list showed minimal overlap with those of HP, non-IPF ILD, and SSc-ILD, but direct comparison of effect sizes indicates that this separation is substantially attributable to unequal statistical power: among genes measurable in all four datasets, only 5 of 29 lacked a concordant sub-threshold signal in at least one comparator disease. That list was not distinguishable from the rest of the tested matrisome, with 20 of the 100 testable genes remaining IPF-preferential against chronic HP (odds ratio 1.37, *p* = 0.25). In total, 8 of the 47 genes evaluable in the GSE184316 IPF-versus-control contrast (1 upregulated and 7 downregulated) were reproduced there; because that series also provides the HP comparator arm and its controls, this replication is not independent. The threshold-based cross-study intersection, rather than the matrisome compartment, is therefore what limits the four-arm analysis.

### 4.1. The IPF-Dominant Matrisome: Three Descriptive Groupings

The 101 IPF-dominant matrisome genes can be organized into three interpretive groupings, each descriptive and based on curated gene function rather than derived as co-expression modules. The within-cohort comparison in GSE150910 tests them gene by gene. IL13 and CCL7 remain IPF-preferential against chronic HP, whereas CCL18 is significantly higher in chronic HP (log2FC −0.63, adjusted *p* = 0.0013), so the immune-associated grouping does not behave as a unit. CCL7’s IPF preference does not survive covariate adjustment either, whereas the assignments of IL13 and CCL18 do (Table S10). Neither the protease and inhibitor grouping (MMP3, MMP12, TIMP3, HYAL2) nor the Wnt-related grouping (WNT4, RSPO1) distinguishes the two diseases once study, platform, and control population are held constant; both are described below as features of the IPF arm relative to its own controls rather than as disease-preferential patterns. The first grouping concerns protease and inhibitor transcripts, and centers on the concurrent upregulation of MMP3 and MMP12 with downregulation of TIMP3 and HYAL2. TIMP3 is the only matrix-bound member of the TIMP family and the inhibitor with the broadest protease coverage [46]. The protease gain and inhibitor loss did not reach significance in HP, non-IPF ILD, or SSc-ILD, but MMP3 was coverage-limited, MMP12 and TIMP3 were power-sensitive, and MMP12 showed a concordant sub-threshold effect in non-IPF ILD, so non-significance in those arms does not establish IPF-selectivity. Because the measurements derive from bulk tissue, a shift in macrophage abundance is an equally plausible explanation for the protease signal. The second grouping, immune-associated fibrogenic signaling, comprises IL13, CCL18, and CCL7. In our data, CCL18 and CCL7 met DEG criteria in IPF alone. IL13 has no HP-arm result in the original table but is measured in the refit, where it meets the criteria (log2FC 0.64, adjusted *p* = 9.6 × 10^−4^), which is why it is one of the five genes that leave the candidate list there; its preference in the direct comparison is unaffected (log2FC 1.90, adjusted *p* = 3.5 × 10^−9^), because the HP effect against controls is much smaller than the IPF effect of 2.35. CCL18, however, is reported as increased and experimentally supported in SSc-ILD in the source study for that arm, and our own comparator values do not support discrimination. CCL18 therefore serves as a worked example of how unequal power and thresholding can generate spurious specificity assignments; it is not evidence that immune inputs to fibrogenesis differ between these diseases. CCR6 is expressed on fibroblast lines from IPF and NSIP lungs but is largely absent in HP and sarcoidosis lines [47], offering a receptor-level basis for selectivity, although the CCL18 receptor remains contested. Anti-IL-13 blockade with tralokinumab did not improve outcomes in IPF [48]. The third grouping, Wnt-related remodeling, includes WNT4 upregulation and RSPO1 downregulation. Reduced RSPO1 would attenuate LGR-dependent potentiation of Wnt signaling at the alveolar progenitor niche [49,50], while WNT4 gain acts on the ligand side; both observations are, to our knowledge, new to IPF, run counter to reports that RSPO2 is increased in IPF, and require independent validation before being treated as disease biology. The divergent behavior of WIF1, an extracellular Wnt antagonist [51,52], reduced in IPF but increased in SSc-ILD, is a separate observation that merits its own investigation. Of the eight genes reproduced in the IPF samples of GSE184316, three belong to these groupings: TIMP3 and HYAL2 from the protease and inhibitor grouping, and RSPO1 from the Wnt-related grouping, all downregulated, alongside CPAMD8, CLEC14A, CTSW, and FGF12, while ADAMTS4 was upregulated.

### 4.2. Hypothesis-Generating Tissue Candidates

The threshold-defined matrisome lists provide hypothesis-generating tissue candidates. No classifier was trained, no case-versus-case diagnostic model was tested, and no external validation was performed. We specifically do not propose CCL18 as discriminating content, because it is prognostic in SSc-ILD and our own comparator values do not separate the diseases. All candidates derive from lung tissue rather than from accessible clinical samples such as blood or bronchoalveolar lavage fluid [53]. Their behavior in accessible compartments is unknown; assay accessibility, particularly for matrix-bound TIMP3, remains to be determined. The arms also differed descriptively, though not significantly, in the direction and category structure of their matrisome response: SSc-ILD was the only arm in which downregulated genes outnumbered upregulated ones (8 up, 17 down); ECM regulators were prominent in both directions of the IPF signature; the non-IPF ILD upregulated signature was co-dominated by ECM glycoproteins and ECM regulators; and the SSc-ILD upregulated signature was dominated by ECM glycoproteins. Because the category differences described above did not reach statistical significance, category structure is reported as description only. Whether such a panel could contribute to the diagnostic challenge posed by these four diseases, which currently requires multidisciplinary review and sometimes surgical biopsy, cannot be determined from these data. Establishing diagnostic utility would require a trained classifier with withheld validation, which we did not attempt here.

### 4.3. Exploratory Therapeutic Hypotheses

The IPF-dominant matrisome list suggests experimental hypotheses, not therapeutic strategies. Bulk transcript abundance does not establish protein abundance, extracellular localization, processing, enzymatic activity, or causal contribution to disease, and for ECM biology in particular, transcript level is a poor proxy for extracellular protein. Disease preference does not predict tractability: IL13 is among the most strongly upregulated IPF-dominant genes, yet both anti-IL-13 antibodies tested in IPF, tralokinumab and lebrikizumab, failed to show benefit [54]. The protease pattern could motivate experimental testing of MMP3 or MMP12 inhibition, although both are reported in rheumatoid arthritis-associated ILD, COPD and systemic sclerosis and are therefore not IPF-selective. We make no therapeutic proposal for TIMP3: the direction of change in our data runs opposite to reports of increased TIMP3 in IPF fibroblastic foci, and Timp3-null mice develop airspace enlargement. A CCL18-CCR6 relationship has been proposed, but the CCL18 receptor remains unresolved, and CCR6 is also expressed on NSIP fibroblasts [47]. The downregulated genes raise a complementary question, namely whether loss of extracellular regulators such as RSPO1 is functionally consequential; this is untested and runs counter to reports that RSPO2 is increased in IPF. This approach differs from nintedanib and pirfenidone, which act on fibroblast effector pathways, and is visible only because the analysis captured downregulated as well as upregulated matrisome genes. Given the failure of ECM-directed agents such as simtuzumab, protein-level and functional confirmation should precede any target selection.

### 4.4. Comparison with Prior Work

Our findings address a specific gap in the existing literature. Prior work using cultured fibroblasts has emphasized similarity between the IPF and SSc fibrotic programs [55], whereas our whole-tissue analysis separates the arms under a threshold-based definition that, as shown above, is strongly power-dependent. Multi-ILD single-cell RNA sequencing atlases include the relevant comparators but have not applied systematic matrisome annotation [56]. A 2024 cross-ILD transcriptomic study identified shared pathways across IPF, fibrotic HP, and CTD-ILD without matrisome-level annotation [40]. More recently, a systematic review and individual-participant meta-analysis developed and externally validated signatures for IPF, HP, NSIP, and SSc-ILD and performed direct comparisons between subtypes [38]. That study is larger, uses individual-participant data and multiple cohorts per disease, and several genes we highlight, including MMP7, COMP, and COL17A1, also appear there. The contribution of the present work is therefore not the cross-disease comparison itself but the systematic application of the Naba matrisome classification to parallel fibrosing ILD arms, so that shared and divergent signal is resolved by ECM structural category.

### 4.5. Limitations

The central limitation of this study is that disease is confounded with study, in the ways enumerated in the Methods. Its principal consequence is one of power: the IPF arm is roughly an order of magnitude larger than the other three (Table 1), giving it greater power to detect modest fold changes that fail to reach significance in the smaller arms, and the effect-size analysis reported above quantifies that consequence, with only 5 of 29 fully measured genes showing no concordant comparator signal. We therefore performed the direct within-study comparison of IPF against chronic HP within GSE150910, reported above. Within that comparison, sequencing batch is completely separated by diagnosis and could not be modeled, so a technical contribution to the difference cannot be excluded; harmonized sample-level reprocessing of all four arms and formal interaction testing remain necessary to extend that result to the other two diseases, and are the subject of planned follow-up work. Of the 101 genes in the candidate list, only 29 were measurable in all four datasets, and 72 were coverage-limited because at least one comparator result was unavailable.

Bulk transcriptomes cannot resolve cell-type contributions, so an apparent increase in a matrisome transcript may reflect a change in the abundance of fibroblasts, epithelial cells, endothelial cells, or macrophages rather than altered transcription within any cell type. This applies particularly to CCL18, IL13, and the matrix metalloproteinases. Computational deconvolution or integration with single-cell and spatial datasets is needed to assign these genes to specific cell populations. The predictions require validation by qRT-PCR, proteomics, or immunohistochemistry, and the cross-sectional design cannot address temporal dynamics of matrix remodeling. The replication reported here is a within-series consistency check rather than an independent test, because the GSE184316 IPF samples come from the same series that supplies the HP arm; the 54 genes that could not be tested there are uninformative, not discordant. Finally, 46 gene symbols in the source annotation are suspected to have been corrupted by spreadsheet date conversion; these were excluded from interpretation but may affect coverage counts.

## 5. Conclusions

This study applies the Naba matrisome classification in parallel across four clinically confusable fibrosing ILDs. Its principal finding is that matrisome genes are strongly over-represented among genes upregulated across multiple cohorts, with a significant increasing trend across sharing degree: 45.7% of the genes shared by all four arms are matrisome members, against 6.8% of the background universe (odds ratio 11.99). Together with the finding that apparent disease preference largely reflects unequal statistical power, this indicates a common ECM program engaged at differing intensities across these diseases. A within-cohort comparison of IPF with chronic HP nonetheless shows that disease-preferential matrisome expression is detectable once disease is no longer confounded with study, platform, and control population; what the threshold-based intersection recovered was not that signal. The IPF-dominant matrisome candidate list organizes into three descriptive groupings: protease and inhibitor transcripts (MMP3 and MMP12 gain paired with loss of the matrix-bound inhibitor TIMP3 and of HYAL2), immune-associated signaling (IL13, CCL18, and CCL7), and Wnt-related remodeling (WNT4 gain paired with reduction in the potentiator RSPO1 at the alveolar progenitor niche). When IPF is compared directly with chronic HP within a single cohort, IL13 and CCL7 remain IPF-preferential, CCL7 only before covariate adjustment, while CCL18 is higher in chronic HP, so none of the three groupings holds as a unit. All three are threshold-defined and power-sensitive, and are offered as hypothesis-generating tissue candidates rather than as diagnostic content or disease-selective therapeutic targets. They also draw attention to loss of extracellular regulators, a compartment largely overlooked by single-marker biomarker studies, as a direction of change worth examining alongside the better-studied gain of matrix and protease genes.

## Figures and Tables

**Table 1:** Summary of transcriptomic datasets used in this study. Counts denote unique participants; where a series contributes more expression profiles than participants, this is stated explicitly. The HP arm (GSE184316) comprises multiple cores per explanted lung and the non-IPF ILD arm (GSE213001) comprises multiple regional samples per donor, so expression profiles exceed participant numbers in both; the GEO record for GSE76808 lists more assay samples than unique participants and the exact participant count could not be determined unambiguously. Platform, control population, and tissue sampling method differ between arms, so disease is confounded with study throughout.

GEO ID	Disease	Platform	Participants (Disease/Control)	Expression Profiles	Tissue Sampling	Control Population	Source of DE Results	Reference
GSE150910	IPF	RNA-seq	103/103	One profile per lung	Explanted or surgical lung tissue	Non-diseased donor lungs	Computed by the authors (DESeq2)	Furusawa et al. 2020 [42]
GSE184316	Fibrotic HP (fHP)	RNA-seq	9/6 lungs	Multiple cores per lung; profiles exceed participants	Multiple regional cores per explanted lung	Unused donor lungs	Computed by the authors (limma on log2 FPKM)	De Sadeleer et al. 2022 [43]
GSE213001	Non-IPF ILD	RNA-seq	9/14	Multiple regional samples per donor; profiles exceed participants	Regional sampling of explanted lung	Donor lungs unsuitable for transplantation	Computed by the authors (limma-voom)	Jaffar et al. 2022 [44]
GSE76808	SSc-ILD	Affymetrix HG-U133A 2.0 microarray	7/4 as reported; not unambiguously determinable from the GEO record	14/4 arrays; GEO record lists more assay samples than participants	Open (surgical) lung biopsy	Non-diseased lung	Computed by the authors (limma)	Christmann et al. 2014 [45]

**Table 2:** Cross-disease comparison of threshold-defined matrisome signatures.

Metric	IPF	HP	Non-IPF ILD	SSc-ILD
Threshold-defined UP (all)	812	2846	413	462
Threshold-defined DOWN (all)	525	533	276	269
Threshold-defined matrisome UP	64	84	36	8
Threshold-defined matrisome DOWN	37	21	18	17
Matrisome fraction among threshold-defined UP	7.9%	3.0%	8.7%	1.7%
Core matrisome fraction among threshold-defined UP	21.9%	15.5%	38.9%	50.0%
Top threshold-defined UP category	Secreted Factors	Secreted Factors	ECM Glycoproteins/ECM Regulators	ECM Glycoproteins
Top threshold-defined DOWN category	ECM Glycoproteins/ECM Regulators	Secreted Factors	ECM-affiliated Proteins	Secreted Factors

**Table 3:** Representative IPF-dominant upregulated matrisome genes. The full list for all 64 upregulated genes is given in Table S2.

Gene	Division	Category	Functional Annotation
COL11A1	Core matrisome	Collagens	Fibrillar collagen; cartilage/fibrosis
COL9A1	Core matrisome	Collagens	FACIT collagen
DMP1	Core matrisome	ECM Glycoproteins	Dentin matrix protein; mineralization
ZPLD1	Core matrisome	ECM Glycoproteins	Zona pellucida-like domain
NELL1	Core matrisome	ECM Glycoproteins	Neural EGFL-like; osteogenic
KERA	Core matrisome	Proteoglycans	Keratocan; corneal proteoglycan
MMP3	Matrisome-associated	ECM Regulators	Stromelysin-1; broad ECM degradation
MMP12	Matrisome-associated	ECM Regulators	Macrophage elastase
PRSS1	Matrisome-associated	ECM Regulators	Trypsin-1; serine protease
SERPINA6	Matrisome-associated	ECM Regulators	Corticosteroid-binding globulin
ITIH6	Matrisome-associated	ECM Regulators	HA matrix stabilizer
ADAMDEC1	Matrisome-associated	ECM Regulators	Decysin; immune metalloprotease
LGALS7	Matrisome-associated	ECM-affiliated Proteins	Galectin-7; epithelial apoptosis
C1QTNF8	Matrisome-associated	ECM-affiliated Proteins	C1q/TNF-related; adipokine
MUCL1	Matrisome-associated	ECM-affiliated Proteins	Small epithelial glycoprotein
ANXA10	Matrisome-associated	ECM-affiliated Proteins	Annexin A10
ITLN1	Matrisome-associated	ECM-affiliated Proteins	Intelectin-1; secreted lectin
IL13	Matrisome-associated	Secreted Factors	Th2 cytokine; pro-fibrotic
BMP15	Matrisome-associated	Secreted Factors	TGF-β superfamily
CCL7	Matrisome-associated	Secreted Factors	Monocyte chemoattractant (MCP-3)
CCL18	Matrisome-associated	Secreted Factors	M2 macrophage; pro-fibrotic biomarker
WNT4	Matrisome-associated	Secreted Factors	Wnt ligand
TNFSF11	Matrisome-associated	Secreted Factors	RANKL; osteoclast/immune
CCL1	Matrisome-associated	Secreted Factors	I-309; Th2-associated chemokine
S100A11	Matrisome-associated	Secreted Factors	Calgizzarin; TGF-β-responsive

**Table 4:** Representative IPF-dominant downregulated matrisome genes. Full statistics and evidence classes for all 37 downregulated genes are given in Table S2.

Gene	Division	Category	Functional Annotation
IL17A	Matrisome-associated	Secreted Factors	Pro-inflammatory; Th17 cytokine
MMP8	Matrisome-associated	ECM Regulators	Neutrophil collagenase
ADAM29	Matrisome-associated	ECM Regulators	Non-catalytic ADAM
SEMA3G	Matrisome-associated	ECM-affiliated Proteins	Anti-angiogenic semaphorin
LEFTY2	Matrisome-associated	Secreted Factors	TGF-β/Nodal antagonist
FGL1	Core matrisome	ECM Glycoproteins	Fibrinogen-like 1; hepatokine
CLEC12B	Matrisome-associated	ECM-affiliated Proteins	Myeloid inhibitory lectin
CPAMD8	Matrisome-associated	ECM Regulators	C3/PZP-like protease inhibitor
NTNG1	Core matrisome	ECM Glycoproteins	Netrin-G1; axon guidance
RSPO1	Core matrisome	ECM Glycoproteins	R-spondin 1; Wnt/stem cell niche
REG4	Matrisome-associated	ECM-affiliated Proteins	regenerating islet-derived family, member 4
INHBA	Matrisome-associated	Secreted Factors	Activin A; TGF-β superfamily
CLEC14A	Matrisome-associated	ECM-affiliated Proteins	Endothelial C-type lectin
HYAL2	Matrisome-associated	ECM Regulators	GPI-anchored hyaluronidase
TIMP3	Matrisome-associated	ECM Regulators	Matrix-bound MMP/ADAM inhibitor
CLEC9A	Matrisome-associated	ECM-affiliated Proteins	Cross-presenting DC lectin
CCN3	Core matrisome	ECM Glycoproteins	NOV; CCN matricellular protein
LAMB4	Core matrisome	ECM Glycoproteins	Laminin β4 chain
COL6A6	Core matrisome	Collagens	Collagen VI α6 chain
CELA2B	Matrisome-associated	ECM Regulators	chymotrypsin-like elastase family, member 2B

## Data Availability

All datasets analyzed in this study are publicly available and were not generated by the authors. Transcriptomic data were obtained from the NCBI Gene Expression Omnibus (GEO, https://www.ncbi.nlm.nih.gov/geo/, accessed on 6 March 2026) under accession numbers GSE150910, GSE184316, GSE213001 and GSE76808. The human matrisome masterlist was obtained from the Matrisome Project (https://matrisomedb.org/, accessed on 26 February 2026).

## Supplementary Materials

The following supporting information can be downloaded at: https://www.mdpi.com/article/10.3390/cimb48090951/s1.

## Abbreviations

The following abbreviations are used in this manuscript:

Abbreviation	Definition
AT2	alveolar type 2
BAL	bronchoalveolar lavage
DEG	differentially expressed gene
ECM	extracellular matrix
EMT	epithelial to mesenchymal transition
FDR	false discovery rate
fHP	fibrotic hypersensitivity pneumonitis
FVC	forced vital capacity
GEO	Gene Expression Omnibus
HP	hypersensitivity pneumonitis
HRCT	high-resolution computed tomography
ILD	interstitial lung disease
IPF	idiopathic pulmonary fibrosis
log_2_FC	log_2_ fold change
MMP	matrix metalloproteinase
NK	natural killer
non-IPF ILD	non-IPF interstitial lung disease
NSIP	non-specific interstitial pneumonia
padj	adjusted *p*-value
SSc-ILD	systemic sclerosis associated interstitial lung disease
TGF-β	transforming growth factor beta
UIP	usual interstitial pneumonia
