# Supplementary material for: Shared and Disease-Preferential Matrisome Programs in Fibrosing Interstitial Lung Disease: Evidence from Cross-Cohort and Within-Cohort Analysis
Source: Curr Issues Mol Biol. 2026 Sep 17;48(9):951. doi: 10.3390/cimb48090951 (PMC13605140; doi:10.3390/cimb48090951)
Supplement: Supplementary file 1 [file cimb-48-00951-s001.zip › Supplementary_Figures_S1-S3.pdf]

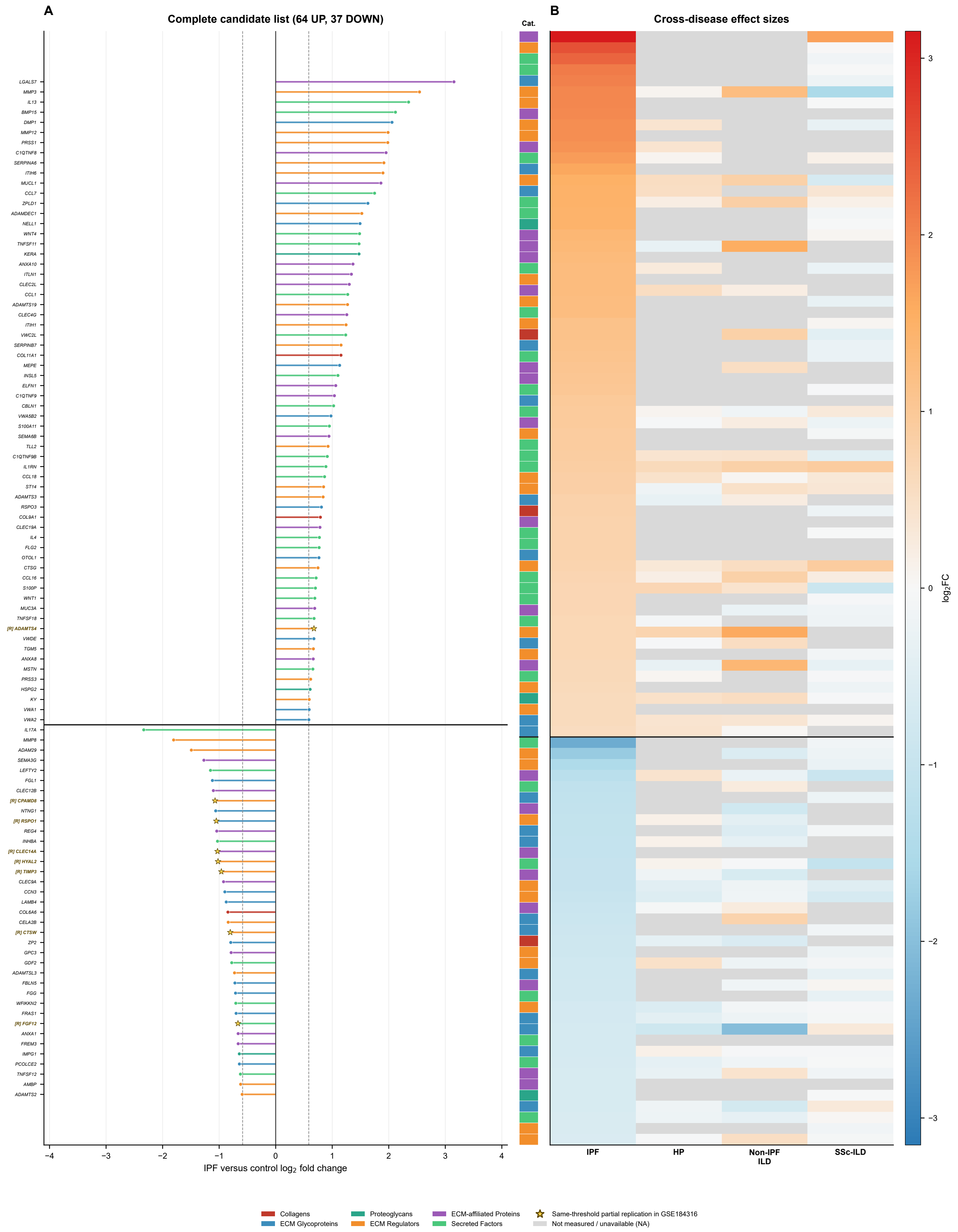

Figure S1. Complete 101-gene threshold-defined IPF-dominant matrisome candidate list (64 UP, 37 DOWN) and cross-disease effect-size heatmap; gray cells denote unavailable measurements, and gold stars denote eight same-threshold partial replications in GSE184316.

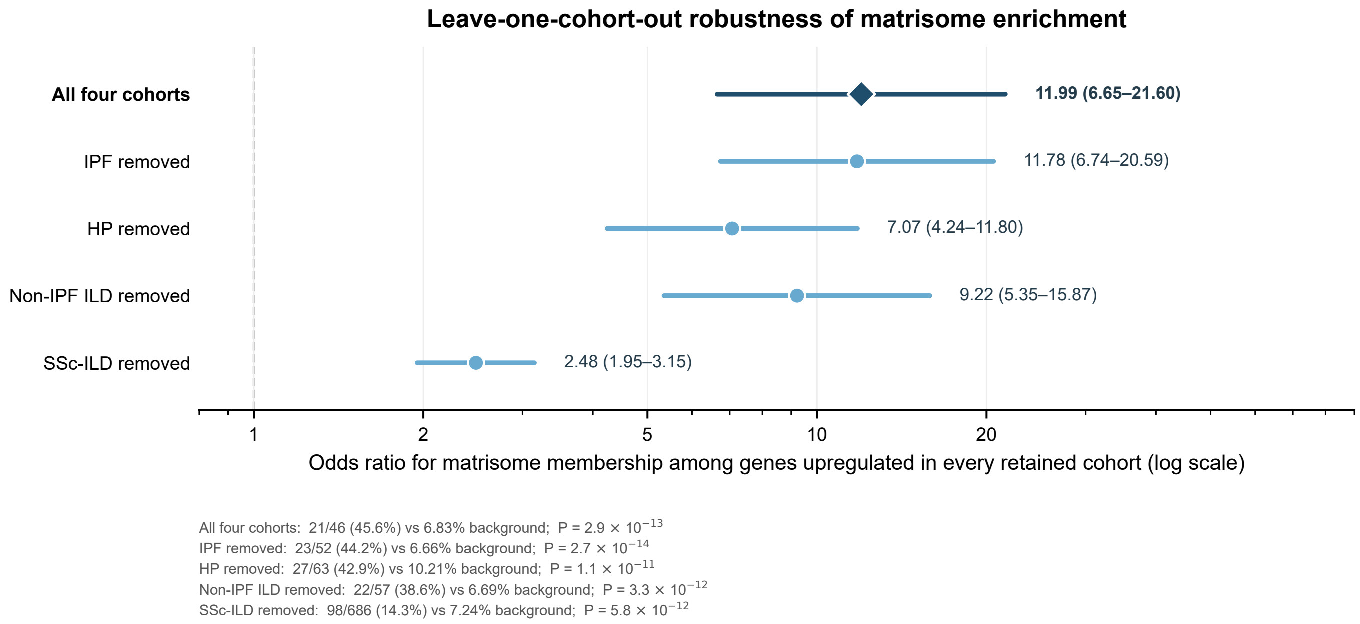

Figure S2. Leave-one-cohort-out robustness of matrisome enrichment, shown as a forest plot of odds ratios with 95% confidence intervals for the full four-cohort analysis and for each leave-one-cohort-out analysis.

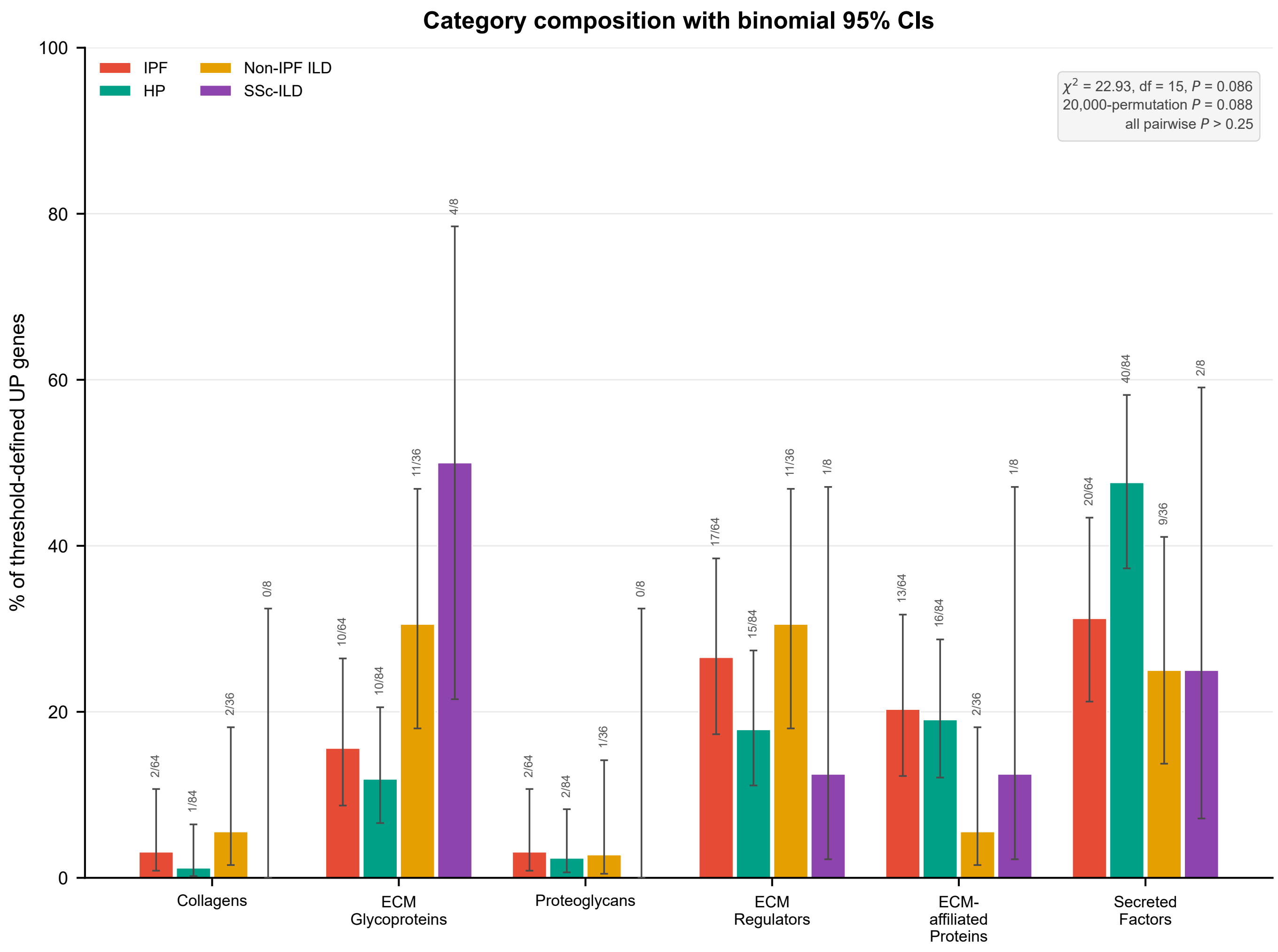

Figure S3. Matrisome category composition of the threshold-defined signatures, with exact counts and Wilson binomial 95% confidence intervals.
