# Supplementary material for: Shared and Disease-Preferential Matrisome Programs in Fibrosing Interstitial Lung Disease: Evidence from Cross-Cohort and Within-Cohort Analysis
Source: Curr Issues Mol Biol. 2026 Sep 17;48(9):951. doi: 10.3390/cimb48090951 (PMC13605140; doi:10.3390/cimb48090951)
Supplement: Supplementary file 1 [file cimb-48-00951-s001.zip › TableS1_CrossDiseaseMatrisome_ILD_DEG_summary.pdf]

Table S1. CrossDiseaseMatrisome ILD DEG summary

Disease_arm	Dataset	Total_up_DEGs	Total_down_DEGs	Total_DEGs	Tested_gene_symbols
IPF	GSE150910	2224	1289	3513	17398
HP	GSE184316	4309	1109	5418	17837
Non-IPF ILD	GSE213001	1608	850	2458	14732
SSc-ILD	GSE76808	613	494	1107	13156
