# Supplementary material for: Shared and Disease-Preferential Matrisome Programs in Fibrosing Interstitial Lung Disease: Evidence from Cross-Cohort and Within-Cohort Analysis
Source: Curr Issues Mol Biol. 2026 Sep 17;48(9):951. doi: 10.3390/cimb48090951 (PMC13605140; doi:10.3390/cimb48090951)
Supplement: Supplementary file 1 [file cimb-48-00951-s001.zip › TableS2_CrossDiseaseMatrisome_ILD_IPF_dominant_101genes.pdf]

Table S2. Complete 101-gene IPF-dominant matrisome candidate list

Gene_symbol	Direction	Matrisome_division	Matrisome_category	Gene_name	IPF_log2FC	IPF_adj_P	IPF_mean_expr
LGALS7	UP	Matrisome-associated	ECM-affiliated Proteins	lectin, galactoside-binding, soluble, 7	3.1529	3.630×10^{-20}	180.9651
MMP3	UP	Matrisome-associated	ECM Regulators	matrix metallopeptidase 3 (stromelysin 1, progelatinase)	2.5439	2.708×10^{-6}	9.5192
IL13	UP	Matrisome-associated	Secreted Factors	interleukin 13	2.3506	1.343×10^{-15}	42.9093
BMP15	UP	Matrisome-associated	Secreted Factors	bone morphogenetic protein 15	2.118	5.408×10^{-10}	4.959
DMP1	UP	Core matrisome	ECM Glycoproteins	dentin matrix acidic phosphoprotein 1	2.0573	4.369×10^{-10}	2.3376
MMP12	UP	Matrisome-associated	ECM Regulators	matrix metallopeptidase 12 (macrophage elastase)	1.9876	4.868×10^{-10}	154.5562
PRSS1	UP	Matrisome-associated	ECM Regulators	protease, serine, 1 (trypsin 1)	1.9831	2.094×10^{-11}	5.7969
C1QTNF8	UP	Matrisome-associated	ECM-affiliated Proteins	C1q and tumor necrosis factor related protein 8	1.9525	1.279×10^{-8}	17.4915
SERPINA6	UP	Matrisome-associated	ECM Regulators	serpin peptidase inhibitor, clade A (alpha-1 antiproteinase, antitrypsin), member 6	1.9138	1.980×10^{-5}	3.9166
ITIH6	UP	Matrisome-associated	ECM Regulators	inter-alpha (globulin) inhibitor H5-like	1.8972	4.532×10^{-5}	1.9154
MUCL1	UP	Matrisome-associated	ECM-affiliated Proteins	mucin-like 1	1.8613	1.330×10^{-4}	23.137
CCL7	UP	Matrisome-associated	Secreted Factors	chemokine (C-C motif) ligand 7	1.7491	9.263×10^{-7}	195.1198
ZPLD1	UP	Core matrisome	ECM Glycoproteins	zona pellucida-like domain containing 1	1.6311	4.736×10^{-11}	3.6913
ADAMDEC1	UP	Matrisome-associated	ECM Regulators	ADAM-like, decysin 1	1.5233	1.187×10^{-8}	137.1593
NELL1	UP	Core matrisome	ECM Glycoproteins	NEL-like 1 (chicken)	1.4917	1.537×10^{-9}	26.2183
WNT4	UP	Matrisome-associated	Secreted Factors	wingless-type MMTV integration site family, member 4	1.4835	2.316×10^{-17}	32.545
TNFSF11	UP	Matrisome-associated	Secreted Factors	tumor necrosis factor (ligand) superfamily, member 11	1.4743	5.294×10^{-12}	10.5396

KERA	UP	Core matrisome	Proteoglycans	keratocan	1.4737	8.008×10^{-15}	10.32
ANXA10	UP	Matrisome-associated	ECM-affiliated Proteins	annexin A10	1.3669	1.023×10^{-10}	20.133
ITLN1	UP	Matrisome-associated	ECM-affiliated Proteins	intelectin 1 (galactofuranose binding)	1.339	0.0013	23.0807
CLEC2L	UP	Matrisome-associated	ECM-affiliated Proteins	C-type lectin domain family 2, member L	1.3015	1.560×10^{-4}	4.9336
CCL1	UP	Matrisome-associated	Secreted Factors	chemokine (C-C motif) ligand 1	1.2741	9.980×10^{-5}	7.6228
ADAMTS19	UP	Matrisome-associated	ECM Regulators	ADAM metallopeptidase with thrombospondin type 1 motif, 19	1.2708	2.432×10^{-5}	7.0165
CLEC4G	UP	Matrisome-associated	ECM-affiliated Proteins	C-type lectin domain family 4, member G	1.2578	2.292×10^{-10}	94.9218
ITIH1	UP	Matrisome-associated	ECM Regulators	inter-alpha (globulin) inhibitor H1	1.2445	1.422×10^{-22}	31.6725
VWC2L	UP	Matrisome-associated	Secreted Factors	von Willebrand factor C domain-containing protein 2-like	1.2384	1.243×10^{-4}	1.6925
SERPINB7	UP	Matrisome-associated	ECM Regulators	serpin peptidase inhibitor, clade B (ovalbumin), member 7	1.1562	0.0036	19.6209
COL11A1	UP	Core matrisome	Collagens	collagen, type XI, alpha 1	1.1548	3.976×10^{-6}	28.7948
MEPE	UP	Core matrisome	ECM Glycoproteins	matrix extracellular phosphoglycoprotein	1.1308	9.752×10^{-4}	5.9236
INSL5	UP	Matrisome-associated	Secreted Factors	insulin-like 5	1.101	6.905×10^{-5}	6.8594
ELFN1	UP	Matrisome-associated	ECM-affiliated Proteins	extracellular leucine-rich repeat and fibronectin type III domain containing 1	1.0623	1.958×10^{-13}	38.6804
C1QTNF9	UP	Matrisome-associated	ECM-affiliated Proteins	C1q and tumor necrosis factor related protein 9	1.0383	3.327×10^{-8}	18.1971
CBLN1	UP	Matrisome-associated	Secreted Factors	cerebellin 1 precursor	1.0245	0.0122	3.8278
VWA5B2	UP	Core matrisome	ECM Glycoproteins	von Willebrand factor A domain containing 5B2	0.9782	4.199×10^{-10}	35.9229
S100A11	UP	Matrisome-associated	Secreted Factors	S100 calcium binding protein A11	0.9494	1.813×10^{-7}	347.9587
SEMA6B	UP	Matrisome-associated	ECM-affiliated Proteins	sema domain, transmembrane domain	0.9425	1.451×10^{-5}	183.2059

				(TM), and cytoplasmic domain, (semaphorin) 6B			
TLL2	UP	Matrisome-associated	ECM Regulators	tolloid-like 2	0.9261	2.679×10^{-6}	4.4689
C1QTNF9B	UP	Matrisome-associated	Secreted Factors	C1q and tumor necrosis factor related protein 9B	0.9126	1.530×10^{-9}	16.3082
IL1RN	UP	Matrisome-associated	Secreted Factors	interleukin 1 receptor antagonist	0.8884	1.176×10^{-5}	2463.5005
CCL18	UP	Matrisome-associated	Secreted Factors	chemokine (C-C motif) ligand 18 (pulmonary and activation-regulated)	0.8644	6.762×10^{-5}	16875.5149
ST14	UP	Matrisome-associated	ECM Regulators	suppression of tumorigenicity 14 (colon carcinoma)	0.847	6.278×10^{-6}	32.862
ADAMTS3	UP	Matrisome-associated	ECM Regulators	ADAM metallopeptidase with thrombospondin type 1 motif, 3	0.8388	0.0011	6.0575
RSPO3	UP	Core matrisome	ECM Glycoproteins	R-spondin 3 homolog (Xenopus laevis)	0.8094	1.108×10^{-5}	72.9223
COL9A1	UP	Core matrisome	Collagens	collagen, type IX, alpha 1	0.7908	5.397×10^{-5}	5.2085
CLEC19A	UP	Matrisome-associated	ECM-affiliated Proteins	C-type lectin domain family 19, member A	0.7833	4.441×10^{-6}	42.6764
IL4	UP	Matrisome-associated	Secreted Factors	interleukin 4	0.773	0.0016	29.1991
FLG2	UP	Matrisome-associated	Secreted Factors	filaggrin family member 2	0.7685	3.653×10^{-5}	3.01
OTOL1	UP	Core matrisome	ECM Glycoproteins	otolin-1	0.7654	0.0015	2.5804
CTSG	UP	Matrisome-associated	ECM Regulators	cathepsin G	0.7481	0.0193	9.4592
CCL16	UP	Matrisome-associated	Secreted Factors	chemokine (C-C motif) ligand 16	0.715	0.0022	18.7804
S100P	UP	Matrisome-associated	Secreted Factors	S100 calcium binding protein P	0.7028	0.0011	1806.6603
WNT1	UP	Matrisome-associated	Secreted Factors	wingless-type MMTV integration site family, member 1	0.6923	6.192×10^{-6}	13.884
MUC3A	UP	Matrisome-associated	ECM-affiliated Proteins	mucin 3A, cell surface associated	0.6901	3.664×10^{-4}	48.858
TNFSF18	UP	Matrisome-associated	Secreted Factors	tumor necrosis factor (ligand) superfamily, member 18	0.6789	2.380×10^{-4}	26.0119

ADAMTS4	UP	Matrisome-associated	ECM Regulators	ADAM metallopeptidase with thrombospondin type 1 motif, 4	0.6766	0.05	1142.3329
VWDE	UP	Core matrisome	ECM Glycoproteins	von Willebrand factor D and EGF domains	0.6758	1.338×10^{-5}	20.9903
TGM5	UP	Matrisome-associated	ECM Regulators	transglutaminase 5	0.6667	2.720×10^{-4}	7.175
ANXA8	UP	Matrisome-associated	ECM-affiliated Proteins	annexin A8	0.6642	2.620×10^{-4}	965.9856
MSTN	UP	Matrisome-associated	Secreted Factors	myostatin	0.6576	8.086×10^{-7}	13.2895
PRSS3	UP	Matrisome-associated	ECM Regulators	protease, serine, 3	0.6185	0.0064	9.9916
HSPG2	UP	Core matrisome	Proteoglycans	heparan sulfate proteoglycan 2	0.6091	2.133×10^{-7}	3904.4242
KY	UP	Matrisome-associated	ECM Regulators	kyphoscoliosis peptidase	0.5933	1.156×10^{-5}	11.8624
VWA1	UP	Core matrisome	ECM Glycoproteins	von Willebrand factor A domain containing 1	0.5919	8.080×10^{-15}	2697.195
VWA2	UP	Core matrisome	ECM Glycoproteins	von Willebrand factor A domain containing 2	0.5871	1.828×10^{-4}	21.7672
IL17A	DOWN	Matrisome-associated	Secreted Factors	interleukin 17A	-2.3338	1.141×10^{-5}	1.7563
MMP8	DOWN	Matrisome-associated	ECM Regulators	matrix metallopeptidase 8 (neutrophil collagenase)	-1.8055	4.312×10^{-5}	18.576
ADAM29	DOWN	Matrisome-associated	ECM Regulators	ADAM metallopeptidase domain 29	-1.4917	5.758×10^{-18}	17.904
SEMA3G	DOWN	Matrisome-associated	ECM-affiliated Proteins	sema domain, immunoglobulin domain (Ig), short basic domain, secreted, (semaphorin) 3G	-1.2699	3.642×10^{-14}	1209.8405
LEFTY2	DOWN	Matrisome-associated	Secreted Factors	left-right determination factor 2	-1.1533	2.129×10^{-12}	72.9606
FGL1	DOWN	Core matrisome	ECM Glycoproteins	fibrinogen-like 1	-1.1194	6.237×10^{-6}	19.5462
CLEC12B	DOWN	Matrisome-associated	ECM-affiliated Proteins	C-type lectin domain family 12, member B	-1.1024	8.147×10^{-13}	43.917
CPAMD8	DOWN	Matrisome-associated	ECM Regulators	C3 and PZP-like, alpha-2-macroglobulin domain containing 8	-1.0724	2.951×10^{-28}	1002.82
NTNG1	DOWN	Core matrisome	ECM Glycoproteins	netrin G1	-1.0608	1.448×10^{-15}	176.7653
RSPO1	DOWN	Core matrisome	ECM Glycoproteins	R-spondin homolog	-1.0502	2.555×10^{-13}	142.4096

				(Xenopus laevis)			
REG4	DOWN	Matrisome-associated	ECM-affiliated Proteins	regenerating islet-derived family, member 4	-1.0422	0.0066	0.9555
INHBA	DOWN	Matrisome-associated	Secreted Factors	inhibin, beta A	-1.0293	2.665×10^{-5}	75.7874
CLEC14A	DOWN	Matrisome-associated	ECM-affiliated Proteins	C-type lectin domain family 14, member A	-1.0288	4.069×10^{-20}	2930.881
HYAL2	DOWN	Matrisome-associated	ECM Regulators	hyaluronoglucosaminidase 2	-1.0202	7.749×10^{-20}	1506.4869
TIMP3	DOWN	Matrisome-associated	ECM Regulators	TIMP metallopeptidase inhibitor 3	-0.9611	6.630×10^{-21}	19718.5292
CLEC9A	DOWN	Matrisome-associated	ECM-affiliated Proteins	C-type lectin domain family 9, member A	-0.9224	1.542×10^{-7}	19.1835
CCN3	DOWN	Core matrisome	ECM Glycoproteins	nephroblastoma overexpressed gene	-0.8991	0.0108	8.4411
LAMB4	DOWN	Core matrisome	ECM Glycoproteins	laminin, beta 4	-0.8746	1.233×10^{-7}	18.8816
COL6A6	DOWN	Core matrisome	Collagens	collagen, type VI, alpha 6	-0.8447	6.342×10^{-8}	559.7382
CELA2B	DOWN	Matrisome-associated	ECM Regulators	chymotrypsin-like elastase family, member 2B	-0.84	1.144×10^{-6}	59.414
CTSW	DOWN	Matrisome-associated	ECM Regulators	cathepsin W	-0.8033	3.037×10^{-8}	142.8097
ZP2	DOWN	Core matrisome	ECM Glycoproteins	zona pellucida glycoprotein 2 (sperm receptor)	-0.7946	1.547×10^{-6}	3.5412
GPC3	DOWN	Matrisome-associated	ECM-affiliated Proteins	glypican 3	-0.7879	1.644×10^{-13}	3730.3365
GDF2	DOWN	Matrisome-associated	Secreted Factors	growth differentiation factor 2	-0.7773	0.0061	2.6826
ADAMTSL3	DOWN	Matrisome-associated	ECM Regulators	ADAMTS-like 3	-0.7282	1.395×10^{-13}	115.3291
FBLN5	DOWN	Core matrisome	ECM Glycoproteins	fibulin 5	-0.721	1.392×10^{-14}	4350.1593
FGG	DOWN	Core matrisome	ECM Glycoproteins	fibrinogen gamma chain	-0.7103	0.0437	2781.7149
WFIKKN2	DOWN	Matrisome-associated	Secreted Factors	WAP, follistatin/kazal, immunoglobulin, kunitz and netrin domain containing 2	-0.7055	4.901×10^{-4}	9.7704
FRAS1	DOWN	Core matrisome	ECM Glycoproteins	Fraser syndrome 1	-0.7001	2.342×10^{-5}	9.9957
FGF12	DOWN	Matrisome-associated	Secreted Factors	fibroblast growth factor 12	-0.6671	3.430×10^{-7}	53.1239
ANXA1	DOWN	Matrisome-associated	ECM-affiliated Proteins	annexin A1	-0.6639	4.776×10^{-5}	4371.9885

FREM3	DOWN	Matrisome-associated	ECM-affiliated Proteins	FRAS1 related extracellular matrix 3	-0.6634	0.0041	13.4301
IMPG1	DOWN	Core matrisome	Proteoglycans	interphotoreceptor matrix proteoglycan 1	-0.6431	4.730×10^{-5}	51.0838
PCOLCE2	DOWN	Core matrisome	ECM Glycoproteins	procollagen C-endopeptidase enhancer 2	-0.6419	6.622×10^{-5}	927.9929
TNFSF12	DOWN	Matrisome-associated	Secreted Factors	tumor necrosis factor (ligand) superfamily, member 12	-0.624	4.114×10^{-17}	2020.2014
AMBP	DOWN	Matrisome-associated	ECM Regulators	alpha-1-microglobulin/bikunin precursor	-0.62	0.0035	5.9179
ADAMTS2	DOWN	Matrisome-associated	ECM Regulators	ADAM metallopeptidase with thrombospondin type 1 motif, 2	-0.593	6.019×10^{-5}	210.1073

101 comparator statistics and coverage

gene_symbol	HP_log2FC	HP_adj_P	nonIPF_log2FC	nonIPF_adj_P	SSc_log2FC	SSc_adj_P	measurable_all_4	comparator_missing_count
LGALS7					1.7137	0.0727	no	2
MMP3					1.174×10^{-4}	0.9994	no	2
IL13					-0.1347	0.4472	no	2
BMP15					-0.0151	0.94	no	2
DMP1					-0.1977	0.2363	no	2
MMP12	0.0833	0.8247	1.2381	0.2198	-1.5495	0.1541	yes	0
PRSS1					-0.0214	0.9078	no	2
C1QTNF8							no	3
SERPINA6	0.4054	0.159			-0.3187	0.0794	no	1
ITIH6							no	3
MUCL1	0.4099	0.2027					no	2
CCL7	0.0962	0.8163			0.168	0.4304	no	1
ZPLD1							no	3
ADAMDEC1	0.5488	0.0831	0.8179	0.4247	-0.6688	0.5888	yes	0
NELL1	0.5412	0.0017			0.37	0.4301	no	1
WNT4	0.2095	0.1889	0.8383	0.1204	0.1509	0.8208	yes	0
TNFSF11					-0.1038	0.6808	no	2
KERA					-0.0169	0.9284	no	2
ANXA10					0.0692	0.8483	no	2
ITLN1	-0.2989	0.1006	1.5678	0.2688			no	1
CLEC2L							no	3

gene_symbol	HP_log2FC	HP_adj_P	nonIPF_log2FC	nonIPF_adj_P	SSc_log2FC	SSc_adj_P	measurable_all_4	comparator_missing_count
CCL1	0.2724	0.205			-0.269	0.0645	no	1
ADAMTS19							no	3
CLEC4G	0.5638	0.0102	0.2163	0.7909			no	1
ITIH1					-0.2998	0.1084	no	2
VWC2L							no	3
SERPINB7					0.053	0.8391	no	2
COL11A1			0.8208	0.329	-0.4397	0.202	no	1
MEPE					-0.2585	0.2439	no	2
INSL5					-0.2626	0.0714	no	2
ELFN1			0.5405	0.2607			no	2
C1QTNF9							no	3
CBLN1					-0.0303	0.8964	no	2
VWA5B2							no	3
S100A11	0.0867	0.5681	-0.1682	0.5338	0.3	0.2304	yes	0
SEMA6B	-0.0385	0.885	0.2355	0.5925	-0.2386	0.2486	yes	0
TLL2					-0.0605	0.7927	no	2
C1QTNF9B							no	3
IL1RN	0.4167	0.1694	0.435	0.4512	-0.4275	0.639	yes	0
CCL18	0.6245	0.1437	0.8354	0.1665	0.9149	0.3306	yes	0
ST14	0.454	0.0014	0.0719	0.8562	0.3369	0.1841	yes	0
ADAMTS3	-0.1856	0.2284	0.4782	0.0963	0.3569	0.2188	yes	0
RSPO3	-0.3161	0.1559	0.2386	0.6645			no	1
COL9A1					-0.2094	0.3527	no	2
CLEC19A							no	3
IL4					-0.0234	0.9061	no	2
FLG2							no	3
OTOL1							no	3
CTSG	0.3428	0.2897	0.5482	0.3632	0.9133	0.0695	yes	0
CCL16	0.1927	0.4213	0.8322	0.2523	0.2397	0.3924	yes	0
S100P	0.707	0.1037	0.4236	0.5228	-0.9063	0.1466	yes	0
WNT1					-0.0466	0.811	no	2
MUC3A			-0.2499	0.6203	-0.1603	0.3947	no	1
TNFSF18	-0.0739	0.6818			-0.197	0.2506	no	1
ADAMTS4	0.7565	0.0821	1.6213	0.0532			no	1
VWDE	-0.0336	0.8555	0.5598	0.24			no	1
TGM5					-0.0999	0.5629	no	2
ANXA8	-0.3187	0.2977	1.3565	0.0577	-0.3422	0.6647	yes	0
MSTN	0.0587	0.7953			-0.0337	0.8918	no	1
PRSS3					-0.2117	0.3953	no	2
HSPG2	0.4798	0.0024	0.5453	0.023	-0.0416	0.9315	yes	0

gene_symbol	HP_log2FC	HP_adj_P	nonIPF_log2FC	nonIPF_adj_P	SSc_log2FC	SSc_adj_P	measurable_all_4	comparator_missing_count
KY							no	3
VWA1	0.4085	0.0125	0.3709	0.1056	0.0968	0.7384	yes	0
VWA2	0.4237	0.0162	0.0402	0.9459			no	1
IL17A					-0.1233	0.5218	no	2
MMP8			-0.5704	0.612	-0.1971	0.1901	no	1
ADAM29					-0.2687	0.1665	no	2
SEMA3G	0.4373	0.0755	-0.2703	0.6943	-0.9206	0.2931	yes	0
LEFTY2			0.2624	0.7271			no	2
FGL1					-0.2331	0.0857	no	2
CLEC12B			-0.7944	0.127			no	2
CPAMD8	0.1541	0.5748	-0.4088	0.2084			no	1
NTNG1			-0.5551	0.1715	-0.1184	0.4188	no	1
RSPO1	0.1237	0.6509	-0.3818	0.3865			no	1
REG4							no	3
INHBA	0.0988	0.7905	-0.0424	0.9471	-1.0415	0.1878	yes	0
CLEC14A	-0.1848	0.358	-0.6579	0.0506			no	1
HYAL2	-0.4552	0.1253	-0.1851	0.6521	-0.4933	0.2553	yes	0
TIMP3	-0.5404	0.0567	-0.155	0.7435	-0.6685	0.1514	yes	0
CLEC9A	-0.0693	0.7451	0.2601	0.659			no	1
CCN3			0.7728	0.0828			no	2
LAMB4					-0.1367	0.3442	no	2
COL6A6	-0.3828	0.3783	-0.6056	0.2992			no	1
CELA2B					-0.2002	0.2093	no	2
CTSW	0.483	0.0133	-0.2096	0.5483	-0.0801	0.7253	yes	0
ZP2					-0.3267	0.1592	no	2
GPC3			-0.1518	0.6696	0.0524	0.9293	no	1
GDF2					-0.343	0.0142	no	2
ADAMTSL3	-0.5738	0.0073	-0.1168	0.7926	-0.0452	0.9362	yes	0
FBLN5	-0.4116	0.0285	-0.2049	0.6012	-0.05	0.9216	yes	0
FGG	-0.8471	0.2127	-2.0332	0.13	0.2968	0.893	yes	0
WFIKKN2							no	3
FRAS1	0.1617	0.565	-0.0273	0.9598	-0.0281	0.8629	yes	0
FGF12	-0.3774	0.0524	-0.1589	0.8137	-0.0142	0.9506	yes	0
ANXA1	-0.2968	0.026	0.4374	0.0112	-0.1407	0.6852	yes	0
FREM3							no	3
IMPG1					-0.0192	0.9018	no	2
PCOLCE2	-0.1826	0.4941	-0.7422	0.1412	0.3085	0.7185	yes	0
TNFSF12	-0.2157	0.0766	-0.3509	0.0274	0.039	0.8459	yes	0
AMBP					-0.1254	0.4999	no	2
ADAMTS2	-0.0256	0.9277	0.5346	0.0212	-0.0716	0.815	yes	0

101 evidence and partial replication

gene_symbol	all_comparator_abs_log2FC_lt_0.3	evidence_class	survives_FC_0.8	survives_FC_1.0	GSE184316_IPF_log2FC	GSE184316_IPF_adj_P	replicated_GSE184316	low_abundance_flag
LGALS7	no	coverage-limited	yes	yes			no	not_evaluated_no_cutoff
MMP3	no	coverage-limited	yes	yes			no	not_evaluated_no_cutoff
IL13	no	coverage-limited	yes	yes			no	not_evaluated_no_cutoff
BMP15	no	coverage-limited	yes	yes			no	not_evaluated_no_cutoff
DMP1	no	coverage-limited	yes	yes			no	not_evaluated_no_cutoff
MMP12	no	power-sensitive	yes	yes	-0.0218	0.9691	no	not_evaluated_no_cutoff
PRSS1	no	coverage-limited	yes	yes			no	not_evaluated_no_cutoff
C1QTNF8	no	coverage-limited	yes	yes			no	not_evaluated_no_cutoff
SERPINA6	no	coverage-limited	yes	yes	0.4558	0.1764	no	not_evaluated_no_cutoff
ITIH6	no	coverage-limited	yes	yes			no	not_evaluated_no_cutoff
MUCL1	no	coverage-limited	yes	yes	0.6933	0.1315	no	not_evaluated_no_cutoff
CCL7	no	coverage-limited	yes	yes	0.4401	0.3914	no	not_evaluated_no_cutoff
ZPLD1	no	coverage-limited	yes	yes			no	not_evaluated_no_cutoff
ADAMDEC1	no	power-sensitive	yes	yes	0.8676	0.0865	no	not_evaluated_no_cutoff
NELL1	no	coverage-limited	yes	yes	0.4593	0.0208	no	not_evaluated_no_cutoff
WNT4	no	power-sensitive	yes	yes	0.4388	0.042	no	not_evaluated_no_cutoff
TNFSF11	no	coverage-limited	yes	yes			no	not_evaluated_no_cutoff
KERA	no	coverage-	yes	yes			no	not_evaluated_n

gene_symbol	all_comparator_abs_log2FC_lt_0.3	evidence_class	survives_FC_0.8	survives_FC_1.0	GSE184316_IPF_log2FC	GSE184316_IPF_adj_P	replicated_GSE184316	low_abundance_flag
		limited						o_cutoff
ANXA10	no	coverage-limited	yes	yes			no	not_evaluated_no_cutoff
ITLN1	no	coverage-limited	yes	yes	0.0304	0.9478	no	not_evaluated_no_cutoff
CLEC2L	no	coverage-limited	yes	yes			no	not_evaluated_no_cutoff
CCL1	no	coverage-limited	yes	yes	0.5099	0.1502	no	not_evaluated_no_cutoff
ADAMTS19	no	coverage-limited	yes	yes			no	not_evaluated_no_cutoff
CLEC4G	no	coverage-limited	yes	yes	0.2944	0.347	no	not_evaluated_no_cutoff
ITIH1	no	coverage-limited	yes	yes			no	not_evaluated_no_cutoff
VWC2L	no	coverage-limited	yes	yes			no	not_evaluated_no_cutoff
SERPINB7	no	coverage-limited	yes	yes			no	not_evaluated_no_cutoff
COL11A1	no	coverage-limited	yes	yes			no	not_evaluated_no_cutoff
MEPE	no	coverage-limited	yes	yes			no	not_evaluated_no_cutoff
INSL5	no	coverage-limited	yes	yes			no	not_evaluated_no_cutoff
ELFN1	no	coverage-limited	yes	yes			no	not_evaluated_no_cutoff
C1QTNF9	no	coverage-limited	yes	yes			no	not_evaluated_no_cutoff
CBLN1	no	coverage-limited	yes	yes			no	not_evaluated_no_cutoff
VWA5B2	no	coverage-limited	yes	no			no	not_evaluated_no_cutoff
S100A11	no	power-sensitive	yes	no	0.0235	0.9264	no	not_evaluated_no_cutoff
SEMA6B	yes	IPF-dominant	yes	no	0.2143	0.5408	no	not_evaluated_no_cutoff
TLL2	no	coverage-limited	yes	no			no	not_evaluated_no_cutoff

gene_symbol	all_comparator_abs_log2FC_lt_0.3	evidence_class	survives_FC_0.8	survives_FC_1.0	GSE184316_IPF_log2FC	GSE184316_IPF_adj_P	replicated_GSE184316	low_abundance_flag
C1QTNF9B	no	coverage-limited	yes	no			no	not_evaluated_no_cutoff
IL1RN	no	power-sensitive	yes	no	0.1614	0.7535	no	not_evaluated_no_cutoff
CCL18	no	power-sensitive	yes	no	1.0784	0.0854	no	not_evaluated_no_cutoff
ST14	no	power-sensitive	yes	no	0.55	0.0019	no	not_evaluated_no_cutoff
ADAMTS3	no	power-sensitive	yes	no	0.0266	0.916	no	not_evaluated_no_cutoff
RSPO3	no	coverage-limited	yes	no	0.0606	0.8791	no	not_evaluated_no_cutoff
COL9A1	no	coverage-limited	no	no			no	not_evaluated_no_cutoff
CLEC19A	no	coverage-limited	no	no			no	not_evaluated_no_cutoff
IL4	no	coverage-limited	no	no			no	not_evaluated_no_cutoff
FLG2	no	coverage-limited	no	no			no	not_evaluated_no_cutoff
OTOL1	no	coverage-limited	no	no			no	not_evaluated_no_cutoff
CTSG	no	power-sensitive	no	no	0.2099	0.7418	no	not_evaluated_no_cutoff
CCL16	no	power-sensitive	no	no	-0.0378	0.9129	no	not_evaluated_no_cutoff
S100P	no	power-sensitive	no	no	0.8842	0.1835	no	not_evaluated_no_cutoff
WNT1	no	coverage-limited	no	no			no	not_evaluated_no_cutoff
MUC3A	no	coverage-limited	no	no			no	not_evaluated_no_cutoff
TNFSF18	no	coverage-limited	no	no	0.2203	0.4429	no	not_evaluated_no_cutoff
ADAMTS4	no	coverage-limited	no	no	1.3012	0.0075	yes	not_evaluated_no_cutoff
VWDE	no	coverage-limited	no	no	0.3379	0.1836	no	not_evaluated_no_cutoff
TGM5	no	coverage-	no	no			no	not_evaluated_n

gene_symbol	all_comparator_abs_log2FC_lt_0.3	evidence_class	survives_FC_0.8	survives_FC_1.0	GSE184316_IPF_log2FC	GSE184316_IPF_adj_P	replicated_GSE184316	low_abundance_flag
		limited						o_cutoff
ANXA8	no	power-sensitive	no	no	-0.3133	0.5698	no	not_evaluated_no_cutoff
MSTN	no	coverage-limited	no	no	0.4975	0.0802	no	not_evaluated_no_cutoff
PRSS3	no	coverage-limited	no	no			no	not_evaluated_no_cutoff
HSPG2	no	power-sensitive	no	no	0.2964	0.236	no	not_evaluated_no_cutoff
KY	no	coverage-limited	no	no			no	not_evaluated_no_cutoff
VWA1	no	power-sensitive	no	no	0.3081	0.1564	no	not_evaluated_no_cutoff
VWA2	no	coverage-limited	no	no	0.4145	0.0726	no	not_evaluated_no_cutoff
IL17A	no	coverage-limited	yes	yes			no	not_evaluated_no_cutoff
MMP8	no	coverage-limited	yes	yes			no	not_evaluated_no_cutoff
ADAM29	no	coverage-limited	yes	yes			no	not_evaluated_no_cutoff
SEMA3G	no	power-sensitive	yes	yes	-0.6334	0.1532	no	not_evaluated_no_cutoff
LEFTY2	no	coverage-limited	yes	yes			no	not_evaluated_no_cutoff
FGL1	no	coverage-limited	yes	yes			no	not_evaluated_no_cutoff
CLEC12B	no	coverage-limited	yes	yes			no	not_evaluated_no_cutoff
CPAMD8	no	coverage-limited	yes	yes	-1.2516	7.113×10^{-9}	yes	not_evaluated_no_cutoff
NTNG1	no	coverage-limited	yes	yes			no	not_evaluated_no_cutoff
RSPO1	no	coverage-limited	yes	yes	-1.4737	9.681×10^{-8}	yes	not_evaluated_no_cutoff
REG4	no	coverage-limited	yes	yes			no	not_evaluated_no_cutoff
INHBA	no	power-sensitive	yes	yes	0.2827	0.5178	no	not_evaluated_no_cutoff

gene_symbol	all_comparator_abs_log2FC_lt_0.3	evidence_class	survives_FC_0.8	survives_FC_1.0	GSE184316_IPF_log2FC	GSE184316_IPF_adj_P	replicated_GSE184316	low_abundance_flag
CLEC14A	no	coverage-limited	yes	yes	-0.8091	0.002	yes	not_evaluated_no_cutoff
HYAL2	no	power-sensitive	yes	yes	-0.8655	0.0152	yes	not_evaluated_no_cutoff
TIMP3	no	power-sensitive	yes	no	-1.1112	0.0012	yes	not_evaluated_no_cutoff
CLEC9A	no	coverage-limited	yes	no	0.1948	0.4956	no	not_evaluated_no_cutoff
CCN3	no	coverage-limited	yes	no			no	not_evaluated_no_cutoff
LAMB4	no	coverage-limited	yes	no			no	not_evaluated_no_cutoff
COL6A6	no	coverage-limited	yes	no	-0.1728	0.7605	no	not_evaluated_no_cutoff
CELA2B	no	coverage-limited	yes	no			no	not_evaluated_no_cutoff
CTSW	no	IPF-dominant	yes	no	-0.794	0.0096	yes	not_evaluated_no_cutoff
ZP2	no	coverage-limited	no	no			no	not_evaluated_no_cutoff
GPC3	no	coverage-limited	no	no			no	not_evaluated_no_cutoff
GDF2	no	coverage-limited	no	no			no	not_evaluated_no_cutoff
ADAMTSL3	no	power-sensitive	no	no	-0.1319	0.6182	no	not_evaluated_no_cutoff
FBLN5	no	power-sensitive	no	no	-0.2656	0.3254	no	not_evaluated_no_cutoff
FGG	no	power-sensitive	no	no	-0.8107	0.407	no	not_evaluated_no_cutoff
WFIKKN2	no	coverage-limited	no	no			no	not_evaluated_no_cutoff
FRAS1	yes	IPF-dominant	no	no	-0.2763	0.4671	no	not_evaluated_no_cutoff
FGF12	no	power-sensitive	no	no	-0.643	0.0038	yes	not_evaluated_no_cutoff
ANXA1	no	IPF-dominant	no	no	0.1766	0.3889	no	not_evaluated_no_cutoff
FREM3	no	coverage-	no	no			no	not_evaluated_n

gene_symbol	all_comparator_abs_log2FC_lt_0.3	evidence_class	survives_FC_0.8	survives_FC_1.0	GSE184316_IPF_log2FC	GSE184316_IPF_adj_P	replicated_GSE184316	low_abundance_flag
		limited						o_cutoff
IMPG1	no	coverage-limited	no	no			no	not_evaluated_no_cutoff
PCOLCE2	no	power-sensitive	no	no	-0.0148	0.9802	no	not_evaluated_no_cutoff
TNFSF12	no	power-sensitive	no	no	-0.3192	0.1379	no	not_evaluated_no_cutoff
AMBP	no	coverage-limited	no	no			no	not_evaluated_no_cutoff
ADAMTS2	no	IPF-dominant	no	no	0.3423	0.2255	no	not_evaluated_no_cutoff
