# Supplementary material for: Shared and Disease-Preferential Matrisome Programs in Fibrosing Interstitial Lung Disease: Evidence from Cross-Cohort and Within-Cohort Analysis
Source: Curr Issues Mol Biol. 2026 Sep 17;48(9):951. doi: 10.3390/cimb48090951 (PMC13605140; doi:10.3390/cimb48090951)
Supplement: Supplementary file 1 [file cimb-48-00951-s001.zip › TableS3a_pathway_gene_sets_membership_and_mapping.pdf]

Table S3a. The 17 author-curated pathway gene sets: complete membership with, for each set, the nearest KEGG, Reactome or MSigDB Hallmark counterpart and its stable identifier. 248 gene-set memberships across 216 distinct genes.

set_name	gene_symbol	n_genes_in_set	nearest_KEGG	nearest_Reactome	nearest_MSigDB_Hallmark
Angiogenesis / VEGF signalling	ANGPT1	12	KEGG hsa04370 (VEGF signaling pathway)	R-HSA-194138 (Signaling by VEGF)	HALLMARK_ANGIOGENESIS
Angiogenesis / VEGF signalling	ANGPT2	12	KEGG hsa04370 (VEGF signaling pathway)	R-HSA-194138 (Signaling by VEGF)	HALLMARK_ANGIOGENESIS
Angiogenesis / VEGF signalling	DLL4	12	KEGG hsa04370 (VEGF signaling pathway)	R-HSA-194138 (Signaling by VEGF)	HALLMARK_ANGIOGENESIS
Angiogenesis / VEGF signalling	FLT1	12	KEGG hsa04370 (VEGF signaling pathway)	R-HSA-194138 (Signaling by VEGF)	HALLMARK_ANGIOGENESIS
Angiogenesis / VEGF signalling	FLT4	12	KEGG hsa04370 (VEGF signaling pathway)	R-HSA-194138 (Signaling by VEGF)	HALLMARK_ANGIOGENESIS
Angiogenesis / VEGF signalling	HIF1A	12	KEGG hsa04370 (VEGF signaling pathway)	R-HSA-194138 (Signaling by VEGF)	HALLMARK_ANGIOGENESIS
Angiogenesis / VEGF signalling	KDR	12	KEGG hsa04370 (VEGF signaling pathway)	R-HSA-194138 (Signaling by VEGF)	HALLMARK_ANGIOGENESIS
Angiogenesis / VEGF signalling	PECAM1	12	KEGG hsa04370 (VEGF signaling pathway)	R-HSA-194138 (Signaling by VEGF)	HALLMARK_ANGIOGENESIS
Angiogenesis / VEGF signalling	PGF	12	KEGG hsa04370 (VEGF signaling pathway)	R-HSA-194138 (Signaling by VEGF)	HALLMARK_ANGIOGENESIS
Angiogenesis / VEGF signalling	TEK	12	KEGG hsa04370 (VEGF signaling pathway)	R-HSA-194138 (Signaling by VEGF)	HALLMARK_ANGIOGENESIS
Angiogenesis / VEGF signalling	VEGFA	12	KEGG hsa04370 (VEGF signaling pathway)	R-HSA-194138 (Signaling by VEGF)	HALLMARK_ANGIOGENESIS
Angiogenesis / VEGF signalling	VWF	12	KEGG hsa04370 (VEGF signaling pathway)	R-HSA-194138 (Signaling by VEGF)	HALLMARK_ANGIOGENESIS
B cell receptor signalling	BANK1	13	KEGG hsa04662 (B cell receptor signaling pathway)	R-HSA-983705 (Signaling by the B Cell Receptor)	no counterpart
B cell receptor signalling	BCL11A	13	KEGG hsa04662 (B cell receptor signaling pathway)	R-HSA-983705 (Signaling by the B Cell Receptor)	no counterpart
B cell receptor signalling	BLNK	13	KEGG hsa04662 (B cell receptor signaling pathway)	R-HSA-983705 (Signaling by the B Cell Receptor)	no counterpart
B cell receptor signalling	CD19	13	KEGG hsa04662 (B cell receptor signaling pathway)	R-HSA-983705 (Signaling by the B Cell Receptor)	no counterpart
B cell receptor signalling	CD22	13	KEGG hsa04662 (B cell receptor signaling pathway)	R-HSA-983705 (Signaling by the B Cell Receptor)	no counterpart
B cell receptor signalling	CD79A	13	KEGG hsa04662 (B cell receptor signaling pathway)	R-HSA-983705 (Signaling by the B Cell Receptor)	no counterpart
B cell receptor signalling	CD79B	13	KEGG hsa04662 (B cell receptor signaling pathway)	R-HSA-983705 (Signaling by the B Cell Receptor)	no counterpart
B cell receptor signalling	CR2	13	KEGG hsa04662 (B cell receptor signaling pathway)	R-HSA-983705 (Signaling by the B Cell Receptor)	no counterpart
B cell receptor signalling	LYN	13	KEGG hsa04662 (B cell receptor signaling pathway)	R-HSA-983705 (Signaling by the B Cell Receptor)	no counterpart
B cell receptor signalling	MS4A1	13	KEGG hsa04662 (B cell receptor	R-HSA-983705 (Signaling by the B	no counterpart

set_name	gene_symbol	n_genes_in_set	nearest_KEGG	nearest_Reactome	nearest_MSigDB_Hallmark
			signaling pathway)	Cell Receptor)	
B cell receptor signalling	PAX5	13	KEGG hsa04662 (B cell receptor signaling pathway)	R-HSA-983705 (Signaling by the B Cell Receptor)	no counterpart
B cell receptor signalling	SPIB	13	KEGG hsa04662 (B cell receptor signaling pathway)	R-HSA-983705 (Signaling by the B Cell Receptor)	no counterpart
B cell receptor signalling	SYK	13	KEGG hsa04662 (B cell receptor signaling pathway)	R-HSA-983705 (Signaling by the B Cell Receptor)	no counterpart
Cellular senescence	CCND1	14	KEGG hsa04218 (Cellular senescence)	R-HSA-2559583 (Cellular Senescence)	no counterpart
Cellular senescence	CDK4	14	KEGG hsa04218 (Cellular senescence)	R-HSA-2559583 (Cellular Senescence)	no counterpart
Cellular senescence	CDK6	14	KEGG hsa04218 (Cellular senescence)	R-HSA-2559583 (Cellular Senescence)	no counterpart
Cellular senescence	CDKN1A	14	KEGG hsa04218 (Cellular senescence)	R-HSA-2559583 (Cellular Senescence)	no counterpart
Cellular senescence	CDKN2A	14	KEGG hsa04218 (Cellular senescence)	R-HSA-2559583 (Cellular Senescence)	no counterpart
Cellular senescence	CXCL8	14	KEGG hsa04218 (Cellular senescence)	R-HSA-2559583 (Cellular Senescence)	no counterpart
Cellular senescence	E2F1	14	KEGG hsa04218 (Cellular senescence)	R-HSA-2559583 (Cellular Senescence)	no counterpart
Cellular senescence	GLB1	14	KEGG hsa04218 (Cellular senescence)	R-HSA-2559583 (Cellular Senescence)	no counterpart
Cellular senescence	IL6	14	KEGG hsa04218 (Cellular senescence)	R-HSA-2559583 (Cellular Senescence)	no counterpart
Cellular senescence	MDM2	14	KEGG hsa04218 (Cellular senescence)	R-HSA-2559583 (Cellular Senescence)	no counterpart
Cellular senescence	RB1	14	KEGG hsa04218 (Cellular senescence)	R-HSA-2559583 (Cellular Senescence)	no counterpart
Cellular senescence	SERPINE1	14	KEGG hsa04218 (Cellular senescence)	R-HSA-2559583 (Cellular Senescence)	no counterpart
Cellular senescence	TGFB1	14	KEGG hsa04218 (Cellular senescence)	R-HSA-2559583 (Cellular Senescence)	no counterpart
Cellular senescence	TP53	14	KEGG hsa04218 (Cellular senescence)	R-HSA-2559583 (Cellular Senescence)	no counterpart
Chemokine signalling	ACKR3	12	KEGG hsa04062 (Chemokine signaling pathway)	R-HSA-380108 (Chemokine receptors bind chemokines)	no counterpart
Chemokine signalling	CCL13	12	KEGG hsa04062 (Chemokine signaling pathway)	R-HSA-380108 (Chemokine receptors bind chemokines)	no counterpart
Chemokine signalling	CCL19	12	KEGG hsa04062 (Chemokine signaling pathway)	R-HSA-380108 (Chemokine receptors bind chemokines)	no counterpart
Chemokine signalling	CCL2	12	KEGG hsa04062 (Chemokine signaling pathway)	R-HSA-380108 (Chemokine receptors bind chemokines)	no counterpart
Chemokine signalling	CCL21	12	KEGG hsa04062 (Chemokine signaling pathway)	R-HSA-380108 (Chemokine receptors bind chemokines)	no counterpart
Chemokine	CCL5	12	KEGG hsa04062	R-HSA-380108	no counterpart

set_name	gene_symbol	n_genes_in_set	nearest_KEGG	nearest_Reactome	nearest_MSigDB_Hallmark
signalling			(Chemokine signaling pathway)	(Chemokine receptors bind chemokines)	
Chemokine signalling	CCR2	12	KEGG hsa04062 (Chemokine signaling pathway)	R-HSA-380108 (Chemokine receptors bind chemokines)	no counterpart
Chemokine signalling	CCR5	12	KEGG hsa04062 (Chemokine signaling pathway)	R-HSA-380108 (Chemokine receptors bind chemokines)	no counterpart
Chemokine signalling	CXCL12	12	KEGG hsa04062 (Chemokine signaling pathway)	R-HSA-380108 (Chemokine receptors bind chemokines)	no counterpart
Chemokine signalling	CXCL13	12	KEGG hsa04062 (Chemokine signaling pathway)	R-HSA-380108 (Chemokine receptors bind chemokines)	no counterpart
Chemokine signalling	CXCL14	12	KEGG hsa04062 (Chemokine signaling pathway)	R-HSA-380108 (Chemokine receptors bind chemokines)	no counterpart
Chemokine signalling	CXCR4	12	KEGG hsa04062 (Chemokine signaling pathway)	R-HSA-380108 (Chemokine receptors bind chemokines)	no counterpart
Collagen formation	COL11A1	16	no counterpart	R-HSA-1474290 (Collagen formation)	no counterpart
Collagen formation	COL14A1	16	no counterpart	R-HSA-1474290 (Collagen formation)	no counterpart
Collagen formation	COL15A1	16	no counterpart	R-HSA-1474290 (Collagen formation)	no counterpart
Collagen formation	COL17A1	16	no counterpart	R-HSA-1474290 (Collagen formation)	no counterpart
Collagen formation	COL1A1	16	no counterpart	R-HSA-1474290 (Collagen formation)	no counterpart
Collagen formation	COL1A2	16	no counterpart	R-HSA-1474290 (Collagen formation)	no counterpart
Collagen formation	COL3A1	16	no counterpart	R-HSA-1474290 (Collagen formation)	no counterpart
Collagen formation	COL5A1	16	no counterpart	R-HSA-1474290 (Collagen formation)	no counterpart
Collagen formation	COL5A2	16	no counterpart	R-HSA-1474290 (Collagen formation)	no counterpart
Collagen formation	COL6A1	16	no counterpart	R-HSA-1474290 (Collagen formation)	no counterpart
Collagen formation	COL6A2	16	no counterpart	R-HSA-1474290 (Collagen formation)	no counterpart
Collagen formation	COL6A3	16	no counterpart	R-HSA-1474290 (Collagen formation)	no counterpart
Collagen formation	LOX	16	no counterpart	R-HSA-1474290 (Collagen formation)	no counterpart
Collagen formation	P4HA1	16	no counterpart	R-HSA-1474290 (Collagen formation)	no counterpart
Collagen formation	PLOD1	16	no counterpart	R-HSA-1474290 (Collagen formation)	no counterpart
Collagen formation	PLOD2	16	no counterpart	R-HSA-1474290 (Collagen formation)	no counterpart
Complement and coagulation	C1QA	16	KEGG hsa04610 (Complement	R-HSA-166658 (Complement	HALLMARK_COMPLEMENT

set_name	gene_symbol	n_genes_in_set	nearest_KEGG	nearest_Reactome	nearest_MSigDB_Hallmark
			and coagulation cascades)	cascade)	
Complement and coagulation	C1QB	16	KEGG hsa04610 (Complement and coagulation cascades)	R-HSA-166658 (Complement cascade)	HALLMARK_COMPLEMENT
Complement and coagulation	C1QC	16	KEGG hsa04610 (Complement and coagulation cascades)	R-HSA-166658 (Complement cascade)	HALLMARK_COMPLEMENT
Complement and coagulation	C2	16	KEGG hsa04610 (Complement and coagulation cascades)	R-HSA-166658 (Complement cascade)	HALLMARK_COMPLEMENT
Complement and coagulation	C3	16	KEGG hsa04610 (Complement and coagulation cascades)	R-HSA-166658 (Complement cascade)	HALLMARK_COMPLEMENT
Complement and coagulation	C4A	16	KEGG hsa04610 (Complement and coagulation cascades)	R-HSA-166658 (Complement cascade)	HALLMARK_COMPLEMENT
Complement and coagulation	C4B	16	KEGG hsa04610 (Complement and coagulation cascades)	R-HSA-166658 (Complement cascade)	HALLMARK_COMPLEMENT
Complement and coagulation	CFB	16	KEGG hsa04610 (Complement and coagulation cascades)	R-HSA-166658 (Complement cascade)	HALLMARK_COMPLEMENT
Complement and coagulation	CFD	16	KEGG hsa04610 (Complement and coagulation cascades)	R-HSA-166658 (Complement cascade)	HALLMARK_COMPLEMENT
Complement and coagulation	F3	16	KEGG hsa04610 (Complement and coagulation cascades)	R-HSA-166658 (Complement cascade)	HALLMARK_COMPLEMENT
Complement and coagulation	PLAT	16	KEGG hsa04610 (Complement and coagulation cascades)	R-HSA-166658 (Complement cascade)	HALLMARK_COMPLEMENT
Complement and coagulation	PLAU	16	KEGG hsa04610 (Complement and coagulation cascades)	R-HSA-166658 (Complement cascade)	HALLMARK_COMPLEMENT
Complement and coagulation	PLAUR	16	KEGG hsa04610 (Complement and coagulation cascades)	R-HSA-166658 (Complement cascade)	HALLMARK_COMPLEMENT
Complement and coagulation	PROC	16	KEGG hsa04610 (Complement and coagulation cascades)	R-HSA-166658 (Complement cascade)	HALLMARK_COMPLEMENT
Complement and coagulation	SERPINE1	16	KEGG hsa04610 (Complement and coagulation cascades)	R-HSA-166658 (Complement cascade)	HALLMARK_COMPLEMENT
Complement and coagulation	VWF	16	KEGG hsa04610 (Complement and coagulation cascades)	R-HSA-166658 (Complement cascade)	HALLMARK_COMPLEMENT
ECM-receptor interaction	CD44	15	KEGG hsa04512 (ECM-receptor interaction)	R-HSA-1474244 (Extracellular matrix organization)	no counterpart
ECM-receptor interaction	COL1A1	15	KEGG hsa04512 (ECM-receptor interaction)	R-HSA-1474244 (Extracellular matrix organization)	no counterpart
ECM-receptor interaction	COL1A2	15	KEGG hsa04512 (ECM-receptor interaction)	R-HSA-1474244 (Extracellular matrix organization)	no counterpart
ECM-receptor interaction	COL3A1	15	KEGG hsa04512 (ECM-receptor interaction)	R-HSA-1474244 (Extracellular matrix organization)	no counterpart
ECM-receptor interaction	FN1	15	KEGG hsa04512 (ECM-receptor interaction)	R-HSA-1474244 (Extracellular matrix organization)	no counterpart

set_name	gene_symbol	n_genes_in_set	nearest_KEGG	nearest_Reactome	nearest_MSigDB_Hallmark
ECM-receptor interaction	ITGA5	15	KEGG hsa04512 (ECM-receptor interaction)	R-HSA-1474244 (Extracellular matrix organization)	no counterpart
ECM-receptor interaction	ITGAV	15	KEGG hsa04512 (ECM-receptor interaction)	R-HSA-1474244 (Extracellular matrix organization)	no counterpart
ECM-receptor interaction	ITGB1	15	KEGG hsa04512 (ECM-receptor interaction)	R-HSA-1474244 (Extracellular matrix organization)	no counterpart
ECM-receptor interaction	ITGB3	15	KEGG hsa04512 (ECM-receptor interaction)	R-HSA-1474244 (Extracellular matrix organization)	no counterpart
ECM-receptor interaction	ITGB6	15	KEGG hsa04512 (ECM-receptor interaction)	R-HSA-1474244 (Extracellular matrix organization)	no counterpart
ECM-receptor interaction	LAMA3	15	KEGG hsa04512 (ECM-receptor interaction)	R-HSA-1474244 (Extracellular matrix organization)	no counterpart
ECM-receptor interaction	LAMB3	15	KEGG hsa04512 (ECM-receptor interaction)	R-HSA-1474244 (Extracellular matrix organization)	no counterpart
ECM-receptor interaction	LAMC2	15	KEGG hsa04512 (ECM-receptor interaction)	R-HSA-1474244 (Extracellular matrix organization)	no counterpart
ECM-receptor interaction	THBS1	15	KEGG hsa04512 (ECM-receptor interaction)	R-HSA-1474244 (Extracellular matrix organization)	no counterpart
ECM-receptor interaction	TNC	15	KEGG hsa04512 (ECM-receptor interaction)	R-HSA-1474244 (Extracellular matrix organization)	no counterpart
Epithelial-mesenchymal transition	ACTA2	18	no counterpart	no counterpart	HALLMARK_EPITHELIAL_MESENCHYMAL_TRANSITION
Epithelial-mesenchymal transition	CDH2	18	no counterpart	no counterpart	HALLMARK_EPITHELIAL_MESENCHYMAL_TRANSITION
Epithelial-mesenchymal transition	COL1A1	18	no counterpart	no counterpart	HALLMARK_EPITHELIAL_MESENCHYMAL_TRANSITION
Epithelial-mesenchymal transition	COL3A1	18	no counterpart	no counterpart	HALLMARK_EPITHELIAL_MESENCHYMAL_TRANSITION
Epithelial-mesenchymal transition	FN1	18	no counterpart	no counterpart	HALLMARK_EPITHELIAL_MESENCHYMAL_TRANSITION
Epithelial-mesenchymal transition	ITGA5	18	no counterpart	no counterpart	HALLMARK_EPITHELIAL_MESENCHYMAL_TRANSITION
Epithelial-mesenchymal transition	MMP2	18	no counterpart	no counterpart	HALLMARK_EPITHELIAL_MESENCHYMAL_TRANSITION
Epithelial-mesenchymal transition	MMP9	18	no counterpart	no counterpart	HALLMARK_EPITHELIAL_MESENCHYMAL_TRANSITION
Epithelial-mesenchymal transition	POSTN	18	no counterpart	no counterpart	HALLMARK_EPITHELIAL_MESENCHYMAL_TRANSITION
Epithelial-mesenchymal transition	SERPINE1	18	no counterpart	no counterpart	HALLMARK_EPITHELIAL_MESENCHYMAL_TRANSITION
Epithelial-mesenchymal transition	SNAI1	18	no counterpart	no counterpart	HALLMARK_EPITHELIAL_MESENCHYMAL_TRANSITION
Epithelial-mesenchymal transition	SNAI2	18	no counterpart	no counterpart	HALLMARK_EPITHELIAL_MESENCHYMAL_TRANSITION
Epithelial-mesenchymal transition	TAGLN	18	no counterpart	no counterpart	HALLMARK_EPITHELIAL_MESENCHYMAL_TRANSITION
Epithelial-mesenchymal transition	TGFBI	18	no counterpart	no counterpart	HALLMARK_EPITHELIAL_MESENCHYMAL_TRANSITION
Epithelial-mesenchymal transition	TWIST1	18	no counterpart	no counterpart	HALLMARK_EPITHELIAL_MESENCHYMAL_TRANSITION
Epithelial-mesenchymal	VIM	18	no counterpart	no counterpart	HALLMARK_EPITHELIAL_MESENCHYMAL_TRANSITION

set_name	gene_symbol	n_genes_in_set	nearest_KEGG	nearest_Reactome	nearest_MSigDB_Hallmark
transition					
Epithelial-mesenchymal transition	ZEB1	18	no counterpart	no counterpart	HALLMARK_EPITHELIAL_MESENCHYMAL_TRANSITION
Epithelial-mesenchymal transition	ZEB2	18	no counterpart	no counterpart	HALLMARK_EPITHELIAL_MESENCHYMAL_TRANSITION
Fibroblast activation	ACTA2	18	no counterpart	no counterpart	no counterpart
Fibroblast activation	CNN1	18	no counterpart	no counterpart	no counterpart
Fibroblast activation	COL1A1	18	no counterpart	no counterpart	no counterpart
Fibroblast activation	COL1A2	18	no counterpart	no counterpart	no counterpart
Fibroblast activation	COL3A1	18	no counterpart	no counterpart	no counterpart
Fibroblast activation	COL5A1	18	no counterpart	no counterpart	no counterpart
Fibroblast activation	COL6A1	18	no counterpart	no counterpart	no counterpart
Fibroblast activation	CTGF	18	no counterpart	no counterpart	no counterpart
Fibroblast activation	FAP	18	no counterpart	no counterpart	no counterpart
Fibroblast activation	FN1	18	no counterpart	no counterpart	no counterpart
Fibroblast activation	LOX	18	no counterpart	no counterpart	no counterpart
Fibroblast activation	MYLK	18	no counterpart	no counterpart	no counterpart
Fibroblast activation	PDGFRA	18	no counterpart	no counterpart	no counterpart
Fibroblast activation	PDGFRB	18	no counterpart	no counterpart	no counterpart
Fibroblast activation	POSTN	18	no counterpart	no counterpart	no counterpart
Fibroblast activation	TAGLN	18	no counterpart	no counterpart	no counterpart
Fibroblast activation	TGFBI	18	no counterpart	no counterpart	no counterpart
Fibroblast activation	THY1	18	no counterpart	no counterpart	no counterpart
Hedgehog signalling	DHH	11	KEGG hsa04340 (Hedgehog signaling pathway)	R-HSA-5358351 (Signaling by Hedgehog)	HALLMARK_HEDGEHOG_SIGNALING
Hedgehog signalling	GLI1	11	KEGG hsa04340 (Hedgehog signaling pathway)	R-HSA-5358351 (Signaling by Hedgehog)	HALLMARK_HEDGEHOG_SIGNALING
Hedgehog signalling	GLI2	11	KEGG hsa04340 (Hedgehog signaling pathway)	R-HSA-5358351 (Signaling by Hedgehog)	HALLMARK_HEDGEHOG_SIGNALING
Hedgehog signalling	GLI3	11	KEGG hsa04340 (Hedgehog signaling pathway)	R-HSA-5358351 (Signaling by Hedgehog)	HALLMARK_HEDGEHOG_SIGNALING
Hedgehog signalling	HHIP	11	KEGG hsa04340 (Hedgehog signaling pathway)	R-HSA-5358351 (Signaling by Hedgehog)	HALLMARK_HEDGEHOG_SIGNALING
Hedgehog signalling	IHH	11	KEGG hsa04340 (Hedgehog signaling pathway)	R-HSA-5358351 (Signaling by Hedgehog)	HALLMARK_HEDGEHOG_SIGNALING
Hedgehog signalling	PTCH1	11	KEGG hsa04340 (Hedgehog signaling pathway)	R-HSA-5358351 (Signaling by Hedgehog)	HALLMARK_HEDGEHOG_SIGNALING
Hedgehog signalling	PTCH2	11	KEGG hsa04340 (Hedgehog signaling pathway)	R-HSA-5358351 (Signaling by Hedgehog)	HALLMARK_HEDGEHOG_SIGNALING
Hedgehog signalling	SHH	11	KEGG hsa04340 (Hedgehog	R-HSA-5358351 (Signaling by	HALLMARK_HEDGEHOG_SIGNALING

set_name	gene_symbol	n_genes_in_set	nearest_KEGG	nearest_Reactome	nearest_MSigDB_Hallmark
			signaling pathway)	Hedgehog)	
Hedgehog signalling	SMO	11	KEGG hsa04340 (Hedgehog signaling pathway)	R-HSA-5358351 (Signaling by Hedgehog)	HALLMARK_HEDGEHOG_SIGNALING
Hedgehog signalling	SUFU	11	KEGG hsa04340 (Hedgehog signaling pathway)	R-HSA-5358351 (Signaling by Hedgehog)	HALLMARK_HEDGEHOG_SIGNALING
Matrix metalloproteinases	MMP1	13	no counterpart	R-HSA-1592389 (Activation of Matrix Metalloproteinases)	no counterpart
Matrix metalloproteinases	MMP10	13	no counterpart	R-HSA-1592389 (Activation of Matrix Metalloproteinases)	no counterpart
Matrix metalloproteinases	MMP11	13	no counterpart	R-HSA-1592389 (Activation of Matrix Metalloproteinases)	no counterpart
Matrix metalloproteinases	MMP12	13	no counterpart	R-HSA-1592389 (Activation of Matrix Metalloproteinases)	no counterpart
Matrix metalloproteinases	MMP14	13	no counterpart	R-HSA-1592389 (Activation of Matrix Metalloproteinases)	no counterpart
Matrix metalloproteinases	MMP2	13	no counterpart	R-HSA-1592389 (Activation of Matrix Metalloproteinases)	no counterpart
Matrix metalloproteinases	MMP3	13	no counterpart	R-HSA-1592389 (Activation of Matrix Metalloproteinases)	no counterpart
Matrix metalloproteinases	MMP7	13	no counterpart	R-HSA-1592389 (Activation of Matrix Metalloproteinases)	no counterpart
Matrix metalloproteinases	MMP8	13	no counterpart	R-HSA-1592389 (Activation of Matrix Metalloproteinases)	no counterpart
Matrix metalloproteinases	MMP9	13	no counterpart	R-HSA-1592389 (Activation of Matrix Metalloproteinases)	no counterpart
Matrix metalloproteinases	TIMP1	13	no counterpart	R-HSA-1592389 (Activation of Matrix Metalloproteinases)	no counterpart
Matrix metalloproteinases	TIMP2	13	no counterpart	R-HSA-1592389 (Activation of Matrix Metalloproteinases)	no counterpart
Matrix metalloproteinases	TIMP3	13	no counterpart	R-HSA-1592389 (Activation of Matrix Metalloproteinases)	no counterpart
NF-kappaB signalling	BCL2A1	13	KEGG hsa04064 (NF-kappa B signaling pathway)	R-HSA-975138 (TRAF6-mediated NF-kB activation)	HALLMARK_TNFA_SIGNALING_VIA_NFKB
NF-kappaB signalling	CCL2	13	KEGG hsa04064 (NF-kappa B signaling pathway)	R-HSA-975138 (TRAF6-mediated NF-kB activation)	HALLMARK_TNFA_SIGNALING_VIA_NFKB
NF-kappaB signalling	CXCL8	13	KEGG hsa04064 (NF-kappa B signaling pathway)	R-HSA-975138 (TRAF6-mediated NF-kB activation)	HALLMARK_TNFA_SIGNALING_VIA_NFKB
NF-kappaB signalling	ICAM1	13	KEGG hsa04064 (NF-kappa B signaling pathway)	R-HSA-975138 (TRAF6-mediated NF-kB activation)	HALLMARK_TNFA_SIGNALING_VIA_NFKB
NF-kappaB signalling	IKBKB	13	KEGG hsa04064 (NF-kappa B signaling pathway)	R-HSA-975138 (TRAF6-mediated NF-kB activation)	HALLMARK_TNFA_SIGNALING_VIA_NFKB
NF-kappaB signalling	IL1B	13	KEGG hsa04064 (NF-kappa B signaling pathway)	R-HSA-975138 (TRAF6-mediated NF-kB activation)	HALLMARK_TNFA_SIGNALING_VIA_NFKB
NF-kappaB signalling	NFKB1	13	KEGG hsa04064 (NF-kappa B signaling pathway)	R-HSA-975138 (TRAF6-mediated NF-kB activation)	HALLMARK_TNFA_SIGNALING_VIA_NFKB

set_name	gene_symbol	n_genes_in_set	nearest_KEGG	nearest_Reactome	nearest_MSigDB_Hallmark
NF-kappaB signalling	NFKBIA	13	KEGG hsa04064 (NF-kappa B signaling pathway)	R-HSA-975138 (TRAF6-mediated NF-kB activation)	HALLMARK_TNFA_SIGNALING_VIA_NFKB
NF-kappaB signalling	RELA	13	KEGG hsa04064 (NF-kappa B signaling pathway)	R-HSA-975138 (TRAF6-mediated NF-kB activation)	HALLMARK_TNFA_SIGNALING_VIA_NFKB
NF-kappaB signalling	TNF	13	KEGG hsa04064 (NF-kappa B signaling pathway)	R-HSA-975138 (TRAF6-mediated NF-kB activation)	HALLMARK_TNFA_SIGNALING_VIA_NFKB
NF-kappaB signalling	TNFAIP3	13	KEGG hsa04064 (NF-kappa B signaling pathway)	R-HSA-975138 (TRAF6-mediated NF-kB activation)	HALLMARK_TNFA_SIGNALING_VIA_NFKB
NF-kappaB signalling	TRAF1	13	KEGG hsa04064 (NF-kappa B signaling pathway)	R-HSA-975138 (TRAF6-mediated NF-kB activation)	HALLMARK_TNFA_SIGNALING_VIA_NFKB
NF-kappaB signalling	TRAF6	13	KEGG hsa04064 (NF-kappa B signaling pathway)	R-HSA-975138 (TRAF6-mediated NF-kB activation)	HALLMARK_TNFA_SIGNALING_VIA_NFKB
NK cell cytotoxicity	CTSW	18	KEGG hsa04650 (Natural killer cell mediated cytotoxicity)	no counterpart	no counterpart
NK cell cytotoxicity	FASLG	18	KEGG hsa04650 (Natural killer cell mediated cytotoxicity)	no counterpart	no counterpart
NK cell cytotoxicity	FCGR3A	18	KEGG hsa04650 (Natural killer cell mediated cytotoxicity)	no counterpart	no counterpart
NK cell cytotoxicity	GNLY	18	KEGG hsa04650 (Natural killer cell mediated cytotoxicity)	no counterpart	no counterpart
NK cell cytotoxicity	GZMA	18	KEGG hsa04650 (Natural killer cell mediated cytotoxicity)	no counterpart	no counterpart
NK cell cytotoxicity	GZMB	18	KEGG hsa04650 (Natural killer cell mediated cytotoxicity)	no counterpart	no counterpart
NK cell cytotoxicity	HCST	18	KEGG hsa04650 (Natural killer cell mediated cytotoxicity)	no counterpart	no counterpart
NK cell cytotoxicity	IFNG	18	KEGG hsa04650 (Natural killer cell mediated cytotoxicity)	no counterpart	no counterpart
NK cell cytotoxicity	KLRC1	18	KEGG hsa04650 (Natural killer cell mediated cytotoxicity)	no counterpart	no counterpart
NK cell cytotoxicity	KLRC2	18	KEGG hsa04650 (Natural killer cell mediated cytotoxicity)	no counterpart	no counterpart
NK cell cytotoxicity	KLRD1	18	KEGG hsa04650 (Natural killer cell mediated cytotoxicity)	no counterpart	no counterpart
NK cell cytotoxicity	NCR1	18	KEGG hsa04650 (Natural killer cell mediated cytotoxicity)	no counterpart	no counterpart
NK cell cytotoxicity	NKG7	18	KEGG hsa04650 (Natural killer cell mediated cytotoxicity)	no counterpart	no counterpart
NK cell cytotoxicity	PRF1	18	KEGG hsa04650	no counterpart	no counterpart

set_name	gene_symbol	n_genes_in_set	nearest_KEGG	nearest_Reactome	nearest_MSigDB_Hallmark
			(Natural killer cell mediated cytotoxicity)		
NK cell cytotoxicity	SH2D1B	18	KEGG hsa04650 (Natural killer cell mediated cytotoxicity)	no counterpart	no counterpart
NK cell cytotoxicity	TYROBP	18	KEGG hsa04650 (Natural killer cell mediated cytotoxicity)	no counterpart	no counterpart
NK cell cytotoxicity	XCL1	18	KEGG hsa04650 (Natural killer cell mediated cytotoxicity)	no counterpart	no counterpart
NK cell cytotoxicity	XCL2	18	KEGG hsa04650 (Natural killer cell mediated cytotoxicity)	no counterpart	no counterpart
Surfactant metabolism	ABCA3	18	no counterpart	R-HSA-5683826 (Surfactant metabolism)	no counterpart
Surfactant metabolism	CLDN18	18	no counterpart	R-HSA-5683826 (Surfactant metabolism)	no counterpart
Surfactant metabolism	CSF2RA	18	no counterpart	R-HSA-5683826 (Surfactant metabolism)	no counterpart
Surfactant metabolism	CSF2RB	18	no counterpart	R-HSA-5683826 (Surfactant metabolism)	no counterpart
Surfactant metabolism	ETV5	18	no counterpart	R-HSA-5683826 (Surfactant metabolism)	no counterpart
Surfactant metabolism	LAMP3	18	no counterpart	R-HSA-5683826 (Surfactant metabolism)	no counterpart
Surfactant metabolism	LPCAT1	18	no counterpart	R-HSA-5683826 (Surfactant metabolism)	no counterpart
Surfactant metabolism	NAPSA	18	no counterpart	R-HSA-5683826 (Surfactant metabolism)	no counterpart
Surfactant metabolism	NKX2-1	18	no counterpart	R-HSA-5683826 (Surfactant metabolism)	no counterpart
Surfactant metabolism	PGC	18	no counterpart	R-HSA-5683826 (Surfactant metabolism)	no counterpart
Surfactant metabolism	SFTA2	18	no counterpart	R-HSA-5683826 (Surfactant metabolism)	no counterpart
Surfactant metabolism	SFTA3	18	no counterpart	R-HSA-5683826 (Surfactant metabolism)	no counterpart
Surfactant metabolism	SFTPA1	18	no counterpart	R-HSA-5683826 (Surfactant metabolism)	no counterpart
Surfactant metabolism	SFTPA2	18	no counterpart	R-HSA-5683826 (Surfactant metabolism)	no counterpart
Surfactant metabolism	SFTPB	18	no counterpart	R-HSA-5683826 (Surfactant metabolism)	no counterpart
Surfactant metabolism	SFTPC	18	no counterpart	R-HSA-5683826 (Surfactant metabolism)	no counterpart
Surfactant metabolism	SFTPD	18	no counterpart	R-HSA-5683826 (Surfactant metabolism)	no counterpart
Surfactant metabolism	SLC34A2	18	no counterpart	R-HSA-5683826 (Surfactant metabolism)	no counterpart
T cell receptor signalling	CD247	13	KEGG hsa04660 (T cell receptor signaling pathway)	R-HSA-202403 (TCR signaling)	no counterpart

set_name	gene_symbol	n_genes_in_set	nearest_KEGG	nearest_Reactome	nearest_MSigDB_Hallmark
T cell receptor signalling	CD3D	13	KEGG hsa04660 (T cell receptor signaling pathway)	R-HSA-202403 (TCR signaling)	no counterpart
T cell receptor signalling	CD3E	13	KEGG hsa04660 (T cell receptor signaling pathway)	R-HSA-202403 (TCR signaling)	no counterpart
T cell receptor signalling	CD3G	13	KEGG hsa04660 (T cell receptor signaling pathway)	R-HSA-202403 (TCR signaling)	no counterpart
T cell receptor signalling	FYN	13	KEGG hsa04660 (T cell receptor signaling pathway)	R-HSA-202403 (TCR signaling)	no counterpart
T cell receptor signalling	ITK	13	KEGG hsa04660 (T cell receptor signaling pathway)	R-HSA-202403 (TCR signaling)	no counterpart
T cell receptor signalling	LAT	13	KEGG hsa04660 (T cell receptor signaling pathway)	R-HSA-202403 (TCR signaling)	no counterpart
T cell receptor signalling	LCK	13	KEGG hsa04660 (T cell receptor signaling pathway)	R-HSA-202403 (TCR signaling)	no counterpart
T cell receptor signalling	PTPRC	13	KEGG hsa04660 (T cell receptor signaling pathway)	R-HSA-202403 (TCR signaling)	no counterpart
T cell receptor signalling	TRAC	13	KEGG hsa04660 (T cell receptor signaling pathway)	R-HSA-202403 (TCR signaling)	no counterpart
T cell receptor signalling	TRBC1	13	KEGG hsa04660 (T cell receptor signaling pathway)	R-HSA-202403 (TCR signaling)	no counterpart
T cell receptor signalling	TRBC2	13	KEGG hsa04660 (T cell receptor signaling pathway)	R-HSA-202403 (TCR signaling)	no counterpart
T cell receptor signalling	ZAP70	13	KEGG hsa04660 (T cell receptor signaling pathway)	R-HSA-202403 (TCR signaling)	no counterpart
TGF-beta signalling	COL1A1	15	KEGG hsa04350 (TGF-beta signaling pathway)	R-HSA-170834 (Signaling by TGF-beta Receptor Complex)	HALLMARK_TGF_BETA_SIGNALING
TGF-beta signalling	COL3A1	15	KEGG hsa04350 (TGF-beta signaling pathway)	R-HSA-170834 (Signaling by TGF-beta Receptor Complex)	HALLMARK_TGF_BETA_SIGNALING
TGF-beta signalling	CTGF	15	KEGG hsa04350 (TGF-beta signaling pathway)	R-HSA-170834 (Signaling by TGF-beta Receptor Complex)	HALLMARK_TGF_BETA_SIGNALING
TGF-beta signalling	FN1	15	KEGG hsa04350 (TGF-beta signaling pathway)	R-HSA-170834 (Signaling by TGF-beta Receptor Complex)	HALLMARK_TGF_BETA_SIGNALING
TGF-beta signalling	ID1	15	KEGG hsa04350 (TGF-beta signaling pathway)	R-HSA-170834 (Signaling by TGF-beta Receptor Complex)	HALLMARK_TGF_BETA_SIGNALING
TGF-beta signalling	ID3	15	KEGG hsa04350 (TGF-beta signaling pathway)	R-HSA-170834 (Signaling by TGF-beta Receptor Complex)	HALLMARK_TGF_BETA_SIGNALING
TGF-beta signalling	POSTN	15	KEGG hsa04350 (TGF-beta signaling pathway)	R-HSA-170834 (Signaling by TGF-beta Receptor Complex)	HALLMARK_TGF_BETA_SIGNALING
TGF-beta signalling	SERPINE1	15	KEGG hsa04350	R-HSA-170834	HALLMARK_TGF_BETA_SIGNALING

set_name	gene_symbol	n_genes_in_set	nearest_KEGG	nearest_Reactome	nearest_MSigDB_Hallmark
			(TGF-beta signaling pathway)	(Signaling by TGF-beta Receptor Complex)	
TGF-beta signalling	SMAD2	15	KEGG hsa04350 (TGF-beta signaling pathway)	R-HSA-170834 (Signaling by TGF-beta Receptor Complex)	HALLMARK_TGF_BETA_SIGNALING
TGF-beta signalling	SMAD3	15	KEGG hsa04350 (TGF-beta signaling pathway)	R-HSA-170834 (Signaling by TGF-beta Receptor Complex)	HALLMARK_TGF_BETA_SIGNALING
TGF-beta signalling	SMAD4	15	KEGG hsa04350 (TGF-beta signaling pathway)	R-HSA-170834 (Signaling by TGF-beta Receptor Complex)	HALLMARK_TGF_BETA_SIGNALING
TGF-beta signalling	SMAD7	15	KEGG hsa04350 (TGF-beta signaling pathway)	R-HSA-170834 (Signaling by TGF-beta Receptor Complex)	HALLMARK_TGF_BETA_SIGNALING
TGF-beta signalling	TGFB1	15	KEGG hsa04350 (TGF-beta signaling pathway)	R-HSA-170834 (Signaling by TGF-beta Receptor Complex)	HALLMARK_TGF_BETA_SIGNALING
TGF-beta signalling	TGFBR1	15	KEGG hsa04350 (TGF-beta signaling pathway)	R-HSA-170834 (Signaling by TGF-beta Receptor Complex)	HALLMARK_TGF_BETA_SIGNALING
TGF-beta signalling	TGFBR2	15	KEGG hsa04350 (TGF-beta signaling pathway)	R-HSA-170834 (Signaling by TGF-beta Receptor Complex)	HALLMARK_TGF_BETA_SIGNALING
Wnt/beta-catenin	APC	13	KEGG hsa04310 (Wnt signaling pathway)	R-HSA-195721 (Signaling by WNT)	HALLMARK_WNT_BETA_CATENIN_SIGNALING
Wnt/beta-catenin	AXIN2	13	KEGG hsa04310 (Wnt signaling pathway)	R-HSA-195721 (Signaling by WNT)	HALLMARK_WNT_BETA_CATENIN_SIGNALING
Wnt/beta-catenin	CTNNB1	13	KEGG hsa04310 (Wnt signaling pathway)	R-HSA-195721 (Signaling by WNT)	HALLMARK_WNT_BETA_CATENIN_SIGNALING
Wnt/beta-catenin	FZD1	13	KEGG hsa04310 (Wnt signaling pathway)	R-HSA-195721 (Signaling by WNT)	HALLMARK_WNT_BETA_CATENIN_SIGNALING
Wnt/beta-catenin	FZD2	13	KEGG hsa04310 (Wnt signaling pathway)	R-HSA-195721 (Signaling by WNT)	HALLMARK_WNT_BETA_CATENIN_SIGNALING
Wnt/beta-catenin	FZD7	13	KEGG hsa04310 (Wnt signaling pathway)	R-HSA-195721 (Signaling by WNT)	HALLMARK_WNT_BETA_CATENIN_SIGNALING
Wnt/beta-catenin	GSK3B	13	KEGG hsa04310 (Wnt signaling pathway)	R-HSA-195721 (Signaling by WNT)	HALLMARK_WNT_BETA_CATENIN_SIGNALING
Wnt/beta-catenin	LEF1	13	KEGG hsa04310 (Wnt signaling pathway)	R-HSA-195721 (Signaling by WNT)	HALLMARK_WNT_BETA_CATENIN_SIGNALING
Wnt/beta-catenin	LRP5	13	KEGG hsa04310 (Wnt signaling pathway)	R-HSA-195721 (Signaling by WNT)	HALLMARK_WNT_BETA_CATENIN_SIGNALING
Wnt/beta-catenin	LRP6	13	KEGG hsa04310 (Wnt signaling pathway)	R-HSA-195721 (Signaling by WNT)	HALLMARK_WNT_BETA_CATENIN_SIGNALING
Wnt/beta-catenin	TCF7	13	KEGG hsa04310 (Wnt signaling pathway)	R-HSA-195721 (Signaling by WNT)	HALLMARK_WNT_BETA_CATENIN_SIGNALING
Wnt/beta-catenin	WNT3A	13	KEGG hsa04310 (Wnt signaling pathway)	R-HSA-195721 (Signaling by WNT)	HALLMARK_WNT_BETA_CATENIN_SIGNALING
Wnt/beta-catenin	WNT5A	13	KEGG hsa04310 (Wnt signaling pathway)	R-HSA-195721 (Signaling by WNT)	HALLMARK_WNT_BETA_CATENIN_SIGNALING
