# Supplementary material for: Shared and Disease-Preferential Matrisome Programs in Fibrosing Interstitial Lung Disease: Evidence from Cross-Cohort and Within-Cohort Analysis
Source: Curr Issues Mol Biol. 2026 Sep 17;48(9):951. doi: 10.3390/cimb48090951 (PMC13605140; doi:10.3390/cimb48090951)
Supplement: Supplementary file 1 [file cimb-48-00951-s001.zip › TableS3b_pathway_gene_sets_summary.pdf]

**Table S3b (summary). One row per pathway set: size, nearest public counterpart in each resource and full gene membership.**

| set_name | n_genes | nearest_KEGG | nearest_Reactome | nearest_MSigDB_Hallmark | genes |
|---|---|---|---|---|---|
| Angiogenesis / VEGF signalling | 12 | KEGG hsa04370 (VEGF signaling pathway) | R-HSA-194138 (Signaling by VEGF) | HALLMARK_ANGIOGENESIS | ANGPT1; ANGPT2; DLL4; FLT1; FLT4; HIF1A; KDR; PECAM1; PGF; TEK; VEGFA; VWF |
| B cell receptor signalling | 13 | KEGG hsa04662 (B cell receptor signaling pathway) | R-HSA-983705 (Signaling by the B Cell Receptor) | no counterpart | BANK1; BCL11A; BLNK; CD19; CD22; CD79A; CD79B; CR2; LYN; MS4A1; PAX5; SPIB; SYK |
| Cellular senescence | 14 | KEGG hsa04218 (Cellular senescence) | R-HSA-2559583 (Cellular Senescence) | no counterpart | CCND1; CDK4; CDK6; CDKN1A; CDKN2A; CXCL8; E2F1; GLB1; IL6; MDM2; RB1; SERPINE1; TGFB1; TP53 |
| Chemokine signalling | 12 | KEGG hsa04062 (Chemokine signaling pathway) | R-HSA-380108 (Chemokine receptors bind chemokines) | no counterpart | ACKR3; CCL13; CCL19; CCL2; CCL21; CCL5; CCR2; CCR5; CXCL12; CXCL13; CXCL14; CXCR4 |
| Collagen formation | 16 | no counterpart | R-HSA-1474290 (Collagen formation) | no counterpart | COL11A1; COL14A1; COL15A1; COL17A1; COL1A1; COL1A2; COL3A1; COL5A1; COL5A2; COL6A1; COL6A2; COL6A3; LOX; P4HA1; PLOD1; PLOD2 |
| Complement and coagulation | 16 | KEGG hsa04610 (Complement and coagulation cascades) | R-HSA-166658 (Complement cascade) | HALLMARK_COMPLEMENT | C1QA; C1QB; C1QC; C2; C3; C4A; C4B; CFB; CFD; F3; PLAT; PLAU; PLAUR; PROC; SERPINE1; VWF |
| ECM-receptor interaction | 15 | KEGG hsa04512 (ECM-receptor interaction) | R-HSA-1474244 (Extracellular matrix organization) | no counterpart | CD44; COL1A1; COL1A2; COL3A1; FN1; ITGA5; ITGAV; ITGB1; ITGB3; ITGB6; LAMA3; LAMB3; LAMC2; THBS1; TNC |
| Epithelial-mesenchymal transition | 18 | no counterpart | no counterpart | HALLMARK_EPITHELIAL_MESENCHYMAL_TRANSITION | ACTA2; CDH2; COL1A1; COL3A1; FN1; ITGA5; MMP2; MMP9; POSTN; SERPINE1; SNAI1; SNAI2; TAGLN; TGFBI; TWIST1; VIM; ZEB1; ZEB2 |
| Fibroblast activation | 18 | no counterpart | no counterpart | no counterpart | ACTA2; CNN1; COL1A1; COL1A2; COL3A1; COL5A1; |

| set_name | n_genes | nearest_KEGG | nearest_Reactome | nearest_MSigDB_Hallmark | genes |
|---|---|---|---|---|---|
| | | | | | COL6A1; CTGF; FAP; FN1; LOX; MYLK; PDGFRA; PDGFRB; POSTN; TAGLN; TGFBI; THY1 |
| Hedgehog signalling | 11 | KEGG hsa04340 (Hedgehog signaling pathway) | R-HSA-5358351 (Signaling by Hedgehog) | HALLMARK_HEDGEHOG_SIGNALING | DHH; GLI1; GLI2; GLI3; HHIP; IHH; PTCH1; PTCH2; SHH; SMO; SUFU |
| Matrix metalloproteinases | 13 | no counterpart | R-HSA-1592389 (Activation of Matrix Metalloproteinases) | no counterpart | MMP1; MMP10; MMP11; MMP12; MMP14; MMP2; MMP3; MMP7; MMP8; MMP9; TIMP1; TIMP2; TIMP3 |
| NF-kappaB signalling | 13 | KEGG hsa04064 (NF-kappa B signaling pathway) | R-HSA-975138 (TRAF6-mediated NF-kB activation) | HALLMARK_TNFA_SIGNALING_VIA_NFKB | BCL2A1; CCL2; CXCL8; ICAM1; IKBKB; IL1B; NFKB1; NFKBIA; RELA; TNF; TNFAIP3; TRAF1; TRAF6 |
| NK cell cytotoxicity | 18 | KEGG hsa04650 (Natural killer cell mediated cytotoxicity) | no counterpart | no counterpart | CTSW; FASLG; FCGR3A; GNLY; GZMA; GZMB; HCST; IFNG; KLRC1; KLRC2; KLRD1; NCR1; NKG7; PRF1; SH2D1B; TYROBP; XCL1; XCL2 |
| Surfactant metabolism | 18 | no counterpart | R-HSA-5683826 (Surfactant metabolism) | no counterpart | ABCA3; CLDN18; CSF2RA; CSF2RB; ETV5; LAMP3; LPCAT1; NAPSA; NKX2-1; PGC; SFTA2; SFTA3; SFTPA1; SFTPA2; SFTPB; SFTPC; SFTPD; SLC34A2 |
| T cell receptor signalling | 13 | KEGG hsa04660 (T cell receptor signaling pathway) | R-HSA-202403 (TCR signaling) | no counterpart | CD247; CD3D; CD3E; CD3G; FYN; ITK; LAT; LCK; PTPRC; TRAC; TRBC1; TRBC2; ZAP70 |
| TGF-beta signalling | 15 | KEGG hsa04350 (TGF-beta signaling pathway) | R-HSA-170834 (Signaling by TGF-beta Receptor Complex) | HALLMARK_TGF_BETA_SIGNALING | COL1A1; COL3A1; CTGF; FN1; ID1; ID3; POSTN; SERPINE1; SMAD2; SMAD3; SMAD4; SMAD7; TGFB1; TGFBR1; TGFBR2 |
| Wnt/beta-catenin | 13 | KEGG hsa04310 (Wnt signaling pathway) | R-HSA-195721 (Signaling by WNT) | HALLMARK_WNT_BETA_CATENIN_SIGNALING | APC; AXIN2; CTNNB1; FZD1; FZD2; FZD7; GSK3B; LEF1; LRP5; LRP6; TCF7; WNT3A; |

| set_name | n_genes | nearest_KEGG | nearest_Reactome | nearest_MSigDB_Hallmark | genes |
|---|---|---|---|---|---|
| | | | | | WNT5A |
