# Supplementary material for: Shared and Disease-Preferential Matrisome Programs in Fibrosing Interstitial Lung Disease: Evidence from Cross-Cohort and Within-Cohort Analysis
Source: Curr Issues Mol Biol. 2026 Sep 17;48(9):951. doi: 10.3390/cimb48090951 (PMC13605140; doi:10.3390/cimb48090951)
Supplement: Supplementary file 1 [file cimb-48-00951-s001.zip › TableS4_cross_disease_effect_sizes_101genes.pdf]

Table S4. Cross-disease effect sizes and evidence class for all 101 genes of the threshold-defined IPF-dominant candidate list. Comparator values are given for every arm, with the number measured, the number concordant at |log2FC| >= 0.3, and the assigned evidence class (IPF-dominant, power-sensitive or coverage-limited).

gene_symbol	gene_name	direction	matrisome_division	matrisome_category	IPF_log2FC	IPF_adj_P	IPF_mean_expression	HP_log2FC	HP_adj_P	HP_status	Non-IPF ILD_log2FC	Non-IPF ILD_adj_P	Non-IPF ILD_status	SSc-ILD_log2FC	SSc-ILD_adj_P	SSc-ILD_status	n_comparators_measured	n_comparators_concordant_ge0.3	n_comparators_ge50pct_IPF_effect	measurable_all_four	evidence_class	GSE184316_IPF_log2FC	GSE184316_concordant_non_independent	interpretation
LGALS7	lectin, galactoside-binding, soluble, 7	UP	Matrisome-associated	ECM-affiliated Proteins	3.153	3.63×10^{-20}	181			not measured			not measured	1.714	0.07269	concordant sub-threshold	1	1	1	no	coverage-limited		no	coverage-limited: comparator data incomplete
MMP3	matrix metallopeptidase 3 (stromelysin 1, progelatinase)	UP	Matrisome-associated	ECM Regulators	2.544	2.71×10^{-6}	9.52			not measured			not measured	0	0.9994	measured, low/discordant effect	1	0	0	no	coverage-limited		no	coverage-limited: comparator data incomplete
IL13	interleukin 13	UP	Matrisome-associated	Secreted Factors	2.351	1.34×10^{-15}	42.91			not measured			not measured	-0.135	0.4472	measured, low/discordant effect	1	0	0	no	coverage-limited		no	coverage-limited: comparator data incomplete
BMP15	bone morphogenetic protein 15	UP	Matrisome-associated	Secreted Factors	2.118	5.41×10^{-10}	4.96			not measured			not measured	-0.015	0.94	measured, low/discordant effect	1	0	0	no	coverage-limited		no	coverage-limited: comparator data incomplete
DMP1	dentin matrix acidic phosphoprotein 1	UP	Core matrisome	ECM Glycoproteins	2.057	4.37×10^{-10}	2.34			not measured			not measured	-0.198	0.2363	measured, low/discordant effect	1	0	0	no	coverage-limited		no	coverage-limited: comparator data incomplete
MMP12	matrix metallopeptidase 12 (macrophage elastase)	UP	Matrisome-associated	ECM Regulators	1.988	4.87×10^{-10}	154.6	0.083	0.8247	measured, low/discordant effect	1.238	0.2198	concordant sub-threshold	-1.55	0.1541	measured, low/discordant effect	3	1	1	yes	power-sensitive	-0.02177	no	power-sensitive: 1 comparator(s) concordant sub-threshold
PRSS1	protease, serine, 1 (trypsin 1)	UP	Matrisome-associated	ECM Regulators	1.983	2.09×10^{-11}	5.8			not measured			not measured	-0.021	0.9078	measured, low/discordant effect	1	0	0	no	coverage-limited		no	coverage-limited: comparator data incomplete
C1QTNF8	C1q and tumor necrosis factor related protein 8	UP	Matrisome-associated	ECM-affiliated Proteins	1.952	1.28×10^{-8}	17.49			not measured			not measured			not measured	0	0	0	no	coverage-limited		no	coverage-limited: comparator data incomplete
SERPINA6	serpin peptidase inhibitor,	UP	Matrisome-associate	ECM Regulators	1.914	1.98×10^{-5}	3.92	0.405	0.159	concordant sub-			not measured	-0.319	0.07942	measured, low/di	2	1	0	no	coverage-limite	0.4558	no	coverage-limite

gene_symbol	gene_name	direction	matrisome_division	matrisome_category	IPF_log2FC	IPF_adj_P	IPF_mean_expression	HP_log2FC	HP_adj_P	HP_status	Non-IPF ILD_log2FC	Non-IPF ILD_adj_P	Non-IPF ILD_status	SSc-ILD_log2FC	SSc-ILD_adj_P	SSc-ILD_status	n_comparators_measured	n_comparators_concordant_ge0.3	n_comparators_ge50pct_IPF_effect	measurable_all_four	evidence_class	GSE184316_IPF_log2FC	GSE184316_concordant_non_independent	interpretation
	clade A (alpha-1 antiproteinase, antitrypsin), member 6		d							threshold						scordant effect					d			d: comparator data incomplete
ITIH6	inter-alpha (globulin) inhibitor H5-like	UP	Matrisome-associated	ECM Regulators	1.897	4.53×10^{-5}	1.92			not measured			not measured			not measured	0	0	0	no	coverage-limited		no	coverage-limited: comparator data incomplete
MUCL1	mucin-like 1	UP	Matrisome-associated	ECM-affiliated Proteins	1.861	1.33×10^{-4}	23.14	0.41	0.2027	concordant sub-threshold			not measured			not measured	1	1	0	no	coverage-limited	0.6933	no	coverage-limited: comparator data incomplete
CCL7	chemokine (C-C motif) ligand 7	UP	Matrisome-associated	Secreted Factors	1.749	9.26×10^{-7}	195.1	0.096	0.8163	measured, low/discordant effect			not measured	0.168	0.4304	measured, low/discordant effect	2	0	0	no	coverage-limited	0.4401	no	coverage-limited: comparator data incomplete
ZPLD1	zona pellucida-like domain containing 1	UP	Core matrisome	ECM Glycoproteins	1.631	4.74×10^{-11}	3.69			not measured			not measured			not measured	0	0	0	no	coverage-limited		no	coverage-limited: comparator data incomplete
ADAMDEC1	ADAM-like, decysin 1	UP	Matrisome-associated	ECM Regulators	1.523	1.19×10^{-8}	137.2	0.549	0.08311	concordant sub-threshold	0.818	0.4247	concordant sub-threshold	-0.669	0.5888	measured, low/discordant effect	3	2	1	yes	power-sensitive	0.8676	no	power-sensitive: 2 comparator(s) concordant sub-threshold
NELL1	NEL-like 1 (chicken)	UP	Core matrisome	ECM Glycoproteins	1.492	1.54×10^{-9}	26.22	0.541	0.001665	concordant sub-threshold			not measured	0.37	0.4301	concordant sub-threshold	2	2	0	no	coverage-limited	0.4593	no	coverage-limited: comparator data incomplete
WNT4	wingless-type MMTV integration site family, member 4	UP	Matrisome-associated	Secreted Factors	1.483	2.32×10^{-17}	32.54	0.209	0.1889	measured, low/discordant effect	0.838	0.1204	concordant sub-threshold	0.151	0.8208	measured, low/discordant effect	3	1	1	yes	power-sensitive	0.4388	no	power-sensitive: 1 comparator(s) concordant sub-threshold
TNFSF11	tumor necrosis factor (ligand) superfamily, member 11	UP	Matrisome-associated	Secreted Factors	1.474	5.29×10^{-12}	10.54			not measured			not measured	-0.104	0.6808	measured, low/discordant effect	1	0	0	no	coverage-limited		no	coverage-limited: comparator data

gene_symbol	gene_name	direction	matrisome_division	matrisome_category	IPF_log2FC	IPF_adj_P	IPF_mean_expression	HP_log2FC	HP_adj_P	HP_status	Non-IPF ILD_log2FC	Non-IPF ILD_adj_P	Non-IPF ILD_status	SSc-ILD_log2FC	SSc-ILD_adj_P	SSc-ILD_status	n_comparators_measured	n_comparators_concordant_ge0.3	n_comparators_ge50pct_IPF_effect	measurable_all_four	evidence_class	GSE184316_IPF_log2FC	GSE184316_concordant_non_independent	interpretation
																								incomplete
KERA	keratocan	UP	Core matrisome	Proteoglycans	1.474	8.01×10^{-15}	10.32			not measured			not measured	-0.017	0.9284	measured, low/discordant effect	1	0	0	no	coverage-limited		no	coverage-limited: comparator data incomplete
ANXA10	annexin A10	UP	Matrisome-associated	ECM-affiliated Proteins	1.367	1.02×10^{-10}	20.13			not measured			not measured	0.069	0.8483	measured, low/discordant effect	1	0	0	no	coverage-limited		no	coverage-limited: comparator data incomplete
ITLN1	intelectin 1 (galactofuranose binding)	UP	Matrisome-associated	ECM-affiliated Proteins	1.339	0.001324	23.08	-0.299	0.1006	measured, low/discordant effect	1.568	0.2688	concordant sub-threshold			not measured	2	1	1	no	coverage-limited	0.03038	no	coverage-limited: comparator data incomplete
CLEC2L	C-type lectin domain family 2, member L	UP	Matrisome-associated	ECM-affiliated Proteins	1.302	1.56×10^{-4}	4.93			not measured			not measured			not measured	0	0	0	no	coverage-limited		no	coverage-limited: comparator data incomplete
CCL1	chemokine (C-C motif) ligand 1	UP	Matrisome-associated	Secreted Factors	1.274	9.98×10^{-5}	7.62	0.272	0.205	measured, low/discordant effect			not measured	-0.269	0.06449	measured, low/discordant effect	2	0	0	no	coverage-limited	0.5099	no	coverage-limited: comparator data incomplete
ADAMTS19	ADAM metallopeptidase with thrombospondin type 1 motif, 19	UP	Matrisome-associated	ECM Regulators	1.271	2.43×10^{-5}	7.02			not measured			not measured			not measured	0	0	0	no	coverage-limited		no	coverage-limited: comparator data incomplete
CLEC4G	C-type lectin domain family 4, member G	UP	Matrisome-associated	ECM-affiliated Proteins	1.258	2.29×10^{-10}	94.92	0.564	0.01016	concordant sub-threshold	0.216	0.7909	measured, low/discordant effect			not measured	2	1	0	no	coverage-limited	0.2944	no	coverage-limited: comparator data incomplete
ITIH1	inter-alpha (globulin) inhibitor H1	UP	Matrisome-associated	ECM Regulators	1.245	1.42×10^{-22}	31.67			not measured			not measured	-0.3	0.1084	measured, low/discordant effect	1	0	0	no	coverage-limited		no	coverage-limited: comparator data incomplete
VWC2L	von Willebrand factor C domain-containing protein 2-like	UP	Matrisome-associated	Secreted Factors	1.238	1.24×10^{-4}	1.69			not measured			not measured			not measured	0	0	0	no	coverage-limited		no	coverage-limited: comparator data incomplete

gene_symbol	gene_name	direction	matrisome_division	matrisome_category	IPF_log2FC	IPF_adj_P	IPF_mean_expression	HP_log2FC	HP_adj_P	HP_status	Non-IPF ILD_log2FC	Non-IPF ILD_adj_P	Non-IPF ILD_status	SSc-ILD_log2FC	SSc-ILD_adj_P	SSc-ILD_status	n_comparators_measured	n_comparators_concordant_ge0.3	n_comparators_ge50pct_IPF_effect	measurable_all_four	evidence_class	GSE184316_IPF_log2FC	GSE184316_concordant_non_independent	interpretation
SERPINB7	serpin peptidase inhibitor, clade B (ovalbumin), member 7	UP	Matrisome-associated	ECM Regulators	1.156	0.003561	19.62			not measured			not measured	0.053	0.8391	measured, low/discordant effect	1	0	0	no	coverage-limited		no	coverage-limited: comparator data incomplete
COL11A1	collagen, type XI, alpha 1	UP	Core matrisome	Collagens	1.155	3.98×10^{-6}	28.79			not measured	0.821	0.329	concordant sub-threshold	-0.44	0.202	measured, low/discordant effect	2	1	1	no	coverage-limited		no	coverage-limited: comparator data incomplete
MEPE	matrix extracellular phosphoglycoprotein	UP	Core matrisome	ECM Glycoproteins	1.131	9.75×10^{-4}	5.92			not measured			not measured	-0.258	0.2439	measured, low/discordant effect	1	0	0	no	coverage-limited		no	coverage-limited: comparator data incomplete
INSL5	insulin-like 5	UP	Matrisome-associated	Secreted Factors	1.101	6.91×10^{-5}	6.86			not measured			not measured	-0.263	0.07144	measured, low/discordant effect	1	0	0	no	coverage-limited		no	coverage-limited: comparator data incomplete
ELFN1	extracellular leucine-rich repeat and fibronectin type III domain containing 1	UP	Matrisome-associated	ECM-affiliated Proteins	1.062	1.96×10^{-13}	38.68			not measured	0.541	0.2607	concordant sub-threshold			not measured	1	1	1	no	coverage-limited		no	coverage-limited: comparator data incomplete
C1QTNF9	C1q and tumor necrosis factor related protein 9	UP	Matrisome-associated	ECM-affiliated Proteins	1.038	3.33×10^{-8}	18.2			not measured			not measured			not measured	0	0	0	no	coverage-limited		no	coverage-limited: comparator data incomplete
CBLN1	cerebellin 1 precursor	UP	Matrisome-associated	Secreted Factors	1.025	0.01225	3.83			not measured			not measured	-0.03	0.8964	measured, low/discordant effect	1	0	0	no	coverage-limited		no	coverage-limited: comparator data incomplete
VWA5B2	von Willebrand factor A domain containing 5B2	UP	Core matrisome	ECM Glycoproteins	0.978	4.20×10^{-10}	35.92			not measured			not measured			not measured	0	0	0	no	coverage-limited		no	coverage-limited: comparator data incomplete
S100A11	S100 calcium binding protein A11	UP	Matrisome-associated	Secreted Factors	0.949	1.81×10^{-7}	348	0.087	0.5681	measured, low/discordant effect	-0.168	0.5338	measured, low/discordant effect	0.3	0.2304	concordant sub-threshold	3	1	0	yes	power-sensitive	0.02353	no	power-sensitive: 1 comparator(s) concordant sub-thresh

gene_symbol	gene_name	direction	matrisome_division	matrisome_category	IPF_log2FC	IPF_adj_P	IPF_mean_expression	HP_log2FC	HP_adj_P	HP_status	Non-IPF ILD_log2FC	Non-IPF ILD_adj_P	Non-IPF ILD_status	SSc-ILD_log2FC	SSc-ILD_adj_P	SSc-ILD_status	n_comparators_measured	n_comparators_concordant_ge0.3	n_comparators_ge50pct_IPF_effect	measurable_all_four	evidence_class	GSE184316_IPF_log2FC	GSE184316_concordant_non_independent	interpretation
																								old
SEMA6B	sema domain, transmembrane domain (TM), and cytoplasmic domain, (semaphorin) 6B	UP	Matrisome-associated	ECM-affiliated Proteins	0.942	1.45×10^{-5}	183.2	-0.039	0.885	measured, low/discordant effect	0.236	0.5925	measured, low/discordant effect	-0.239	0.2486	measured, low/discordant effect	3	0	0	yes	IPF-dominant	0.2143	no	IPF-dominant: no concordant comparator signal
TLL2	tolloid-like 2	UP	Matrisome-associated	ECM Regulators	0.926	2.68×10^{-6}	4.47			not measured			not measured	-0.06	0.7927	measured, low/discordant effect	1	0	0	no	coverage-limited		no	coverage-limited: comparator data incomplete
C1QTNF9B	C1q and tumor necrosis factor related protein 9B	UP	Matrisome-associated	Secreted Factors	0.913	1.53×10^{-9}	16.31			not measured			not measured			not measured	0	0	0	no	coverage-limited		no	coverage-limited: comparator data incomplete
IL1RN	interleukin 1 receptor antagonist	UP	Matrisome-associated	Secreted Factors	0.888	1.18×10^{-5}	2464	0.417	0.1694	concordant sub-threshold	0.435	0.4512	concordant sub-threshold	-0.428	0.639	measured, low/discordant effect	3	2	0	yes	power-sensitive	0.1614	no	power-sensitive: 2 comparator(s) concordant sub-threshold
CCL18	chemokine (C-C motif) ligand 18 (pulmonary and activation-regulated)	UP	Matrisome-associated	Secreted Factors	0.864	6.76×10^{-5}	1.688×10^{4}	0.625	0.1437	concordant sub-threshold	0.835	0.1665	concordant sub-threshold	0.915	0.3306	concordant sub-threshold	3	3	3	yes	power-sensitive	1.078	no	power-sensitive: 3 comparator(s) concordant sub-threshold
ST14	suppression of tumorigenicity 14 (colon carcinoma)	UP	Matrisome-associated	ECM Regulators	0.847	6.28×10^{-6}	32.86	0.454	0.001377	concordant sub-threshold	0.072	0.8562	measured, low/discordant effect	0.337	0.1841	concordant sub-threshold	3	2	1	yes	power-sensitive	0.55	no	power-sensitive: 2 comparator(s) concordant sub-threshold
ADAMTS3	ADAM metallopeptidase with thrombospondin type 1 motif, 3	UP	Matrisome-associated	ECM Regulators	0.839	0.001054	6.06	-0.186	0.2284	measured, low/discordant effect	0.478	0.09627	concordant sub-threshold	0.357	0.2188	concordant sub-threshold	3	2	1	yes	power-sensitive	0.02664	no	power-sensitive: 2 comparator(s) concordant sub-threshold
RSPO3	R-spondin 3 homolog (Xenopus laevis)	UP	Core matrisome	ECM Glycoproteins	0.809	1.11×10^{-5}	72.92	-0.316	0.1559	measured, low/discordant effect	0.239	0.6645	measured, low/discordant effect			not measured	2	0	0	no	coverage-limited	0.06059	no	coverage-limited: comparator

gene_symbol	gene_name	direction	matrisome_division	matrisome_category	IPF_log2FC	IPF_adj_P	IPF_mean_expression	HP_log2FC	HP_adj_P	HP_status	Non-IPF ILD_log2FC	Non-IPF ILD_adj_P	Non-IPF ILD_status	SSc-ILD_log2FC	SSc-ILD_adj_P	SSc-ILD_status	n_comparators_measured	n_comparators_concordant_ge0.3	n_comparators_ge50pct_IPF_effect	measurable_all_four	evidence_class	GSE184316_IPF_log2FC	GSE184316_concordant_non_independent	interpretation
																								data incomplete
COL9A1	collagen, type IX, alpha 1	UP	Core matrisome	Collagens	0.791	5.40×10^{-5}	5.21			not measured			not measured	-0.209	0.3527	measured, low/discordant effect	1	0	0	no	coverage-limited		no	coverage-limited: comparator data incomplete
CLEC19A	C-type lectin domain family 19, member A	UP	Matrisome-associated	ECM-affiliated Proteins	0.783	4.44×10^{-6}	42.68			not measured			not measured			not measured	0	0	0	no	coverage-limited		no	coverage-limited: comparator data incomplete
IL4	interleukin 4	UP	Matrisome-associated	Secreted Factors	0.773	0.001581	29.2			not measured			not measured	-0.023	0.9061	measured, low/discordant effect	1	0	0	no	coverage-limited		no	coverage-limited: comparator data incomplete
FLG2	filaggrin family member 2	UP	Matrisome-associated	Secreted Factors	0.768	3.65×10^{-5}	3.01			not measured			not measured			not measured	0	0	0	no	coverage-limited		no	coverage-limited: comparator data incomplete
OTOL1	otolin-1	UP	Core matrisome	ECM Glycoproteins	0.765	0.001488	2.58			not measured			not measured			not measured	0	0	0	no	coverage-limited		no	coverage-limited: comparator data incomplete
CTSG	cathepsin G	UP	Matrisome-associated	ECM Regulators	0.748	0.01928	9.46	0.343	0.2897	concordant sub-threshold	0.548	0.3632	concordant sub-threshold	0.913	0.06948	concordant sub-threshold	3	3	2	yes	power-sensitive	0.2099	no	power-sensitive: 3 comparator(s) concordant sub-threshold
CCL16	chemokine (C-C motif) ligand 16	UP	Matrisome-associated	Secreted Factors	0.715	0.002205	18.78	0.193	0.4213	measured, low/discordant effect	0.832	0.2523	concordant sub-threshold	0.24	0.3924	measured, low/discordant effect	3	1	1	yes	power-sensitive	-0.03783	no	power-sensitive: 1 comparator(s) concordant sub-threshold
S100P	S100 calcium binding protein P	UP	Matrisome-associated	Secreted Factors	0.703	0.001146	1807	0.707	0.1037	concordant sub-threshold	0.424	0.5228	concordant sub-threshold	-0.906	0.1466	measured, low/discordant effect	3	2	2	yes	power-sensitive	0.8842	no	power-sensitive: 2 comparator(s) concordant sub-thresh

gene_symbol	gene_name	direction	matrisome_division	matrisome_category	IPF_log2FC	IPF_adj_P	IPF_mean_expression	HP_log2FC	HP_adj_P	HP_status	Non-IPF ILD_log2FC	Non-IPF ILD_adj_P	Non-IPF ILD_status	SSc-ILD_log2FC	SSc-ILD_adj_P	SSc-ILD_status	n_comparators_measured	n_comparators_concordant_ge0.3	n_comparators_ge50pct_IPF_effect	measurable_all_four	evidence_class	GSE184316_IPF_log2FC	GSE184316_concordant_non_independent	interpretation
																								old
WNT1	wingless-type MMTV integration site family, member 1	UP	Matrisome-associated	Secreted Factors	0.692	6.19×10^{-6}	13.88			not measured			not measured	-0.047	0.811	measured, low/discordant effect	1	0	0	no	coverage-limited		no	coverage-limited: comparator data incomplete
MUC3A	mucin 3A, cell surface associated	UP	Matrisome-associated	ECM-affiliated Proteins	0.69	3.66×10^{-4}	48.86			not measured	-0.25	0.6203	measured, low/discordant effect	-0.16	0.3947	measured, low/discordant effect	2	0	0	no	coverage-limited		no	coverage-limited: comparator data incomplete
TNFSF18	tumor necrosis factor (ligand) superfamily, member 18	UP	Matrisome-associated	Secreted Factors	0.679	2.38×10^{-4}	26.01	-0.074	0.6818	measured, low/discordant effect			not measured	-0.197	0.2506	measured, low/discordant effect	2	0	0	no	coverage-limited	0.2203	no	coverage-limited: comparator data incomplete
ADAMTS4	ADAM metallopeptidase with thrombospondin type 1 motif, 4	UP	Matrisome-associated	ECM Regulators	0.677	0.04996	1142	0.756	0.08207	concordant sub-threshold	1.621	0.05317	concordant sub-threshold			not measured	2	2	2	no	coverage-limited	1.301	yes	coverage-limited: comparator data incomplete
VWDE	von Willebrand factor D and EGF domains	UP	Core matrisome	ECM Glycoproteins	0.676	1.34×10^{-5}	20.99	-0.034	0.8555	measured, low/discordant effect	0.56	0.24	concordant sub-threshold			not measured	2	1	1	no	coverage-limited	0.3379	no	coverage-limited: comparator data incomplete
TGM5	transglutaminase 5	UP	Matrisome-associated	ECM Regulators	0.667	2.72×10^{-4}	7.17			not measured			not measured	-0.1	0.5629	measured, low/discordant effect	1	0	0	no	coverage-limited		no	coverage-limited: comparator data incomplete
ANXA8	annexin A8	UP	Matrisome-associated	ECM-affiliated Proteins	0.664	2.62×10^{-4}	966	-0.319	0.2977	measured, low/discordant effect	1.356	0.0577	concordant sub-threshold	-0.342	0.6647	measured, low/discordant effect	3	1	1	yes	power-sensitive	-0.3133	no	power-sensitive: 1 comparator(s) concordant sub-threshold
MSTN	myostatin	UP	Matrisome-associated	Secreted Factors	0.658	8.09×10^{-7}	13.29	0.059	0.7953	measured, low/discordant effect			not measured	-0.034	0.8918	measured, low/discordant effect	2	0	0	no	coverage-limited	0.4975	no	coverage-limited: comparator data incomplete
PRSS3	protease, serine, 3	UP	Matrisome-associated	ECM Regulators	0.618	0.006438	9.99			not measured			not measured	-0.212	0.3953	measured, low/discordant effect	1	0	0	no	coverage-limited		no	coverage-limited: comparator data

gene_symbol	gene_name	direction	matrisome_division	matrisome_category	IPF_log2FC	IPF_adj_P	IPF_mean_expression	HP_log2FC	HP_adj_P	HP_status	Non-IPF ILD_log2FC	Non-IPF ILD_adj_P	Non-IPF ILD_status	SSc-ILD_log2FC	SSc-ILD_adj_P	SSc-ILD_status	n_comparators_measured	n_comparators_concordant_ge0.3	n_comparators_ge50pct_IPF_effect	measurable_all_four	evidence_class	GSE184316_IPF_log2FC	GSE184316_concordant_non_independent	interpretation
																								incomplete
HSPG2	heparan sulfate proteoglycan 2	UP	Core matrisome	Proteoglycans	0.609	2.13×10^{-7}	3904	0.48	0.002353	concordant sub-threshold	0.545	0.02302	concordant sub-threshold	-0.042	0.9315	measured, low/discordant effect	3	2	2	yes	power-sensitive	0.2964	no	power-sensitive: 2 comparator(s) concordant sub-threshold
KY	kyphoscoliosis peptidase	UP	Matrisome-associated	ECM Regulators	0.593	1.16×10^{-5}	11.86			not measured			not measured			not measured	0	0	0	no	coverage-limited		no	coverage-limited: comparator data incomplete
VWA1	von Willebrand factor A domain containing 1	UP	Core matrisome	ECM Glycoproteins	0.592	8.08×10^{-15}	2697	0.408	0.01253	concordant sub-threshold	0.371	0.1056	concordant sub-threshold	0.097	0.7384	measured, low/discordant effect	3	2	2	yes	power-sensitive	0.3081	no	power-sensitive: 2 comparator(s) concordant sub-threshold
VWA2	von Willebrand factor A domain containing 2	UP	Core matrisome	ECM Glycoproteins	0.587	1.83×10^{-4}	21.77	0.424	0.01624	concordant sub-threshold	0.04	0.9459	measured, low/discordant effect			not measured	2	1	1	no	coverage-limited	0.4145	no	coverage-limited: comparator data incomplete
IL17A	interleukin 17A	DOWN	Matrisome-associated	Secreted Factors	-2.334	1.14×10^{-5}	1.76			not measured			not measured	-0.123	0.5218	measured, low/discordant effect	1	0	0	no	coverage-limited		no	coverage-limited: comparator data incomplete
MMP8	matrix metallopeptidase 8 (neutrophil collagenase)	DOWN	Matrisome-associated	ECM Regulators	-1.806	4.31×10^{-5}	18.58			not measured	-0.57	0.612	concordant sub-threshold	-0.197	0.1901	measured, low/discordant effect	2	1	0	no	coverage-limited		no	coverage-limited: comparator data incomplete
ADAM29	ADAM metallopeptidase domain 29	DOWN	Matrisome-associated	ECM Regulators	-1.492	5.76×10^{-18}	17.9			not measured			not measured	-0.269	0.1665	measured, low/discordant effect	1	0	0	no	coverage-limited		no	coverage-limited: comparator data incomplete
SEMA3G	sema domain, immunoglobulin domain (Ig), short basic domain, secreted, (semaphorin) 3G	DOWN	Matrisome-associated	ECM-affiliated Proteins	-1.27	3.64×10^{-14}	1210	0.437	0.07547	measured, low/discordant effect	-0.27	0.6943	measured, low/discordant effect	-0.921	0.2931	concordant sub-threshold	3	1	1	yes	power-sensitive	-0.6334	no	power-sensitive: 1 comparator(s) concordant sub-threshold

gene_symbol	gene_name	direction	matrisome_division	matrisome_category	IPF_log2FC	IPF_adj_P	IPF_mean_expression	HP_log2FC	HP_adj_P	HP_status	Non-IPF ILD_log2FC	Non-IPF ILD_adj_P	Non-IPF ILD_status	SSc-ILD_log2FC	SSc-ILD_adj_P	SSc-ILD_status	n_comparators_measured	n_comparators_concordant_ge0.3	n_comparators_ge50pct_IPF_effect	measurable_all_four	evidence_class	GSE184316_IPF_log2FC	GSE184316_concordant_non_independent	interpretation
LEFTY2	left-right determination factor 2	DOWN	Matrisome-associated	Secreted Factors	-1.153	2.13×10^{-12}	72.96			not measured	0.262	0.7271	measured, low/discordant effect			not measured	1	0	0	no	coverage-limited		no	coverage-limited: comparator data incomplete
FGL1	fibrinogen-like 1	DOWN	Core matrisome	ECM Glycoproteins	-1.119	6.24×10^{-6}	19.55			not measured			not measured	-0.233	0.08572	measured, low/discordant effect	1	0	0	no	coverage-limited		no	coverage-limited: comparator data incomplete
CLEC12B	C-type lectin domain family 12, member B	DOWN	Matrisome-associated	ECM-affiliated Proteins	-1.102	8.15×10^{-13}	43.92			not measured	-0.794	0.127	concordant sub-threshold			not measured	1	1	1	no	coverage-limited		no	coverage-limited: comparator data incomplete
CPAMD8	C3 and PZP-like, alpha-2-macroglobulin domain containing 8	DOWN	Matrisome-associated	ECM Regulators	-1.072	2.95×10^{-28}	1003	0.154	0.5748	measured, low/discordant effect	-0.409	0.2084	concordant sub-threshold			not measured	2	1	0	no	coverage-limited	-1.252	yes	coverage-limited: comparator data incomplete
NTNG1	netrin G1	DOWN	Core matrisome	ECM Glycoproteins	-1.061	1.45×10^{-15}	176.8			not measured	-0.555	0.1715	concordant sub-threshold	-0.118	0.4188	measured, low/discordant effect	2	1	1	no	coverage-limited		no	coverage-limited: comparator data incomplete
RSPO1	R-spondin homolog (Xenopus laevis)	DOWN	Core matrisome	ECM Glycoproteins	-1.05	2.56×10^{-13}	142.4	0.124	0.6509	measured, low/discordant effect	-0.382	0.3865	concordant sub-threshold			not measured	2	1	0	no	coverage-limited	-1.474	yes	coverage-limited: comparator data incomplete
REG4	regenerating islet-derived family, member 4	DOWN	Matrisome-associated	ECM-affiliated Proteins	-1.042	0.006607	0.96			not measured			not measured			not measured	0	0	0	no	coverage-limited		no	coverage-limited: comparator data incomplete
INHBA	inhibin, beta A	DOWN	Matrisome-associated	Secreted Factors	-1.029	2.66×10^{-5}	75.79	0.099	0.7905	measured, low/discordant effect	-0.042	0.9471	measured, low/discordant effect	-1.042	0.1878	concordant sub-threshold	3	1	1	yes	power-sensitive	0.2827	no	power-sensitive: 1 comparator(s) concordant sub-threshold
CLEC14A	C-type lectin domain family 14, member A	DOWN	Matrisome-associated	ECM-affiliated Proteins	-1.029	4.07×10^{-20}	2931	-0.185	0.358	measured, low/discordant effect	-0.658	0.05055	concordant sub-threshold			not measured	2	1	1	no	coverage-limited	-0.8091	yes	coverage-limited: comparator data incom

gene_symbol	gene_name	direction	matrisome_division	matrisome_category	IPF_log2FC	IPF_adj_P	IPF_mean_expression	HP_log2FC	HP_adj_P	HP_status	Non-IPF ILD_log2FC	Non-IPF ILD_adj_P	Non-IPF ILD_status	SSc-ILD_log2FC	SSc-ILD_adj_P	SSc-ILD_status	n_comparators_measured	n_comparators_concordant_ge0.3	n_comparators_ge50pct_IPF_effect	measurable_all_four	evidence_class	GSE184316_IPF_log2FC	GSE184316_concordant_non_independent	interpretation
																								plete
HYAL2	hyaluronoglucosaminidase 2	DOWN	Matrisome-associated	ECM Regulators	-1.02	7.75×10^{-20}	1506	-0.455	0.1253	concordant sub-threshold	-0.185	0.6521	measured, low/discordant effect	-0.493	0.2553	concordant sub-threshold	3	2	0	yes	power-sensitive	-0.8655	yes	power-sensitive: 2 comparator(s) concordant sub-threshold
TIMP3	TIMP metallopeptidase inhibitor 3	DOWN	Matrisome-associated	ECM Regulators	-0.961	6.63×10^{-21}	1.972×10^{4}	-0.54	0.05666	concordant sub-threshold	-0.155	0.7435	measured, low/discordant effect	-0.669	0.1514	concordant sub-threshold	3	2	2	yes	power-sensitive	-1.111	yes	power-sensitive: 2 comparator(s) concordant sub-threshold
CLEC9A	C-type lectin domain family 9, member A	DOWN	Matrisome-associated	ECM-affiliated Proteins	-0.922	1.54×10^{-7}	19.18	-0.069	0.7451	measured, low/discordant effect	0.26	0.659	measured, low/discordant effect			not measured	2	0	0	no	coverage-limited	0.1948	no	coverage-limited: comparator data incomplete
CCN3	nephroblastoma overexpressed gene	DOWN	Core matrisome	ECM Glycoproteins	-0.899	0.01076	8.44			not measured	0.773	0.08278	measured, low/discordant effect			not measured	1	0	0	no	coverage-limited		no	coverage-limited: comparator data incomplete
LAMB4	laminin, beta 4	DOWN	Core matrisome	ECM Glycoproteins	-0.875	1.23×10^{-7}	18.88			not measured			not measured	-0.137	0.3442	measured, low/discordant effect	1	0	0	no	coverage-limited		no	coverage-limited: comparator data incomplete
COL6A6	collagen, type VI, alpha 6	DOWN	Core matrisome	Collagens	-0.845	6.34×10^{-8}	559.7	-0.383	0.3783	concordant sub-threshold	-0.606	0.2992	concordant sub-threshold			not measured	2	2	1	no	coverage-limited	-0.1728	no	coverage-limited: comparator data incomplete
CELA2B	chymotrypsin-like elastase family, member 2B	DOWN	Matrisome-associated	ECM Regulators	-0.84	1.14×10^{-6}	59.41			not measured			not measured	-0.2	0.2093	measured, low/discordant effect	1	0	0	no	coverage-limited		no	coverage-limited: comparator data incomplete
CTSW	cathepsin W	DOWN	Matrisome-associated	ECM Regulators	-0.803	3.04×10^{-8}	142.8	0.483	0.01327	measured, low/discordant effect	-0.21	0.5483	measured, low/discordant effect	-0.08	0.7253	measured, low/discordant effect	3	0	0	yes	IPF-dominant	-0.794	yes	IPF-dominant: no concordant comparator signal
ZP2	zona pellucida glycoprotein 2 (sperm	DOWN	Core matrisome	ECM Glycoproteins	-0.795	1.55×10^{-6}	3.54			not measured			not measured	-0.327	0.1592	concordant sub-thresh	1	1	0	no	coverage-limited		no	coverage-limited:

gene_symbol	gene_name	direction	matrisome_division	matrisome_category	IPF_log2FC	IPF_adj_P	IPF_mean_expression	HP_log2FC	HP_adj_P	HP_status	Non-IPF ILD_log2FC	Non-IPF ILD_adj_P	Non-IPF ILD_status	SSc-ILD_log2FC	SSc-ILD_adj_P	SSc-ILD_status	n_comparators_measured	n_comparators_concordant_ge0.3	n_comparators_ge50pct_IPF_effect	measurable_all_four	evidence_class	GSE184316_IPF_log2FC	GSE184316_concordant_non_independent	interpretation
	receptor)															old								comparator data incomplete
GPC3	glypican 3	DOWN	Matrisome-associated	ECM-affiliated Proteins	-0.788	1.64×10^{-13}	3730			not measured	-0.152	0.6696	measured, low/discordant effect	0.052	0.9293	measured, low/discordant effect	2	0	0	no	coverage-limited		no	coverage-limited: comparator data incomplete
GDF2	growth differentiation factor 2	DOWN	Matrisome-associated	Secreted Factors	-0.777	0.006114	2.68			not measured			not measured	-0.343	0.01419	concordant sub-threshold	1	1	0	no	coverage-limited		no	coverage-limited: comparator data incomplete
ADAMTSL3	ADAMTS-like 3	DOWN	Matrisome-associated	ECM Regulators	-0.728	1.40×10^{-13}	115.3	-0.574	0.007341	concordant sub-threshold	-0.117	0.7926	measured, low/discordant effect	-0.045	0.9362	measured, low/discordant effect	3	1	1	yes	power-sensitive	-0.1319	no	power-sensitive: 1 comparator(s) concordant sub-threshold
FBLN5	fibulin 5	DOWN	Core matrisome	ECM Glycoproteins	-0.721	1.39×10^{-14}	4350	-0.412	0.02854	concordant sub-threshold	-0.205	0.6012	measured, low/discordant effect	-0.05	0.9216	measured, low/discordant effect	3	1	1	yes	power-sensitive	-0.2656	no	power-sensitive: 1 comparator(s) concordant sub-threshold
FGG	fibrinogen gamma chain	DOWN	Core matrisome	ECM Glycoproteins	-0.71	0.04368	2782	-0.847	0.2127	concordant sub-threshold	-2.033	0.13	concordant sub-threshold	0.297	0.893	measured, low/discordant effect	3	2	2	yes	power-sensitive	-0.8107	no	power-sensitive: 2 comparator(s) concordant sub-threshold
WFIKKN2	WAP, follistatin/kazal, immunoglobulin, kunitz and netrin domain containing 2	DOWN	Matrisome-associated	Secreted Factors	-0.705	4.90×10^{-4}	9.77			not measured			not measured			not measured	0	0	0	no	coverage-limited		no	coverage-limited: comparator data incomplete
FRAS1	Fraser syndrome 1	DOWN	Core matrisome	ECM Glycoproteins	-0.7	2.34×10^{-5}	10	0.162	0.565	measured, low/discordant effect	-0.027	0.9598	measured, low/discordant effect	-0.028	0.8629	measured, low/discordant effect	3	0	0	yes	IPF-dominant	-0.2763	no	IPF-dominant: no concordant comparator signal
FGF12	fibroblast growth factor 12	DOWN	Matrisome-associated	Secreted Factors	-0.667	3.43×10^{-7}	53.12	-0.377	0.05237	concordant sub-threshold	-0.159	0.8137	measured, low/discordant	-0.014	0.9506	measured, low/discordant	3	1	1	yes	power-sensitive	-0.643	yes	power-sensitive: 1 compa

gene_symbol	gene_name	direction	matrisome_division	matrisome_category	IPF_log2FC	IPF_adj_P	IPF_mean_expression	HP_log2FC	HP_adj_P	HP_status	Non-IPF ILD_log2FC	Non-IPF ILD_adj_P	Non-IPF ILD_status	SSc-ILD_log2FC	SSc-ILD_adj_P	SSc-ILD_status	n_comparators_measured	n_comparators_concordant_ge0.3	n_comparators_ge50pct_IPF_effect	measurable_all_four	evidence_class	GSE184316_IPF_log2FC	GSE184316_concordant_non_independent	interpretation
													effect			effect								rator(s) concordant sub-threshold
ANXA1	annexin A1	DOWN	Matrisome-associated	ECM-affiliated Proteins	-0.664	4.78×10^{-5}	4372	-0.297	0.02598	measured, low/discordant effect	0.437	0.01117	measured, low/discordant effect	-0.141	0.6852	measured, low/discordant effect	3	0	0	yes	IPF-dominant	0.1766	no	IPF-dominant: no concordant comparator signal
FREM3	FRAS1 related extracellular matrix 3	DOWN	Matrisome-associated	ECM-affiliated Proteins	-0.663	0.00407	13.43			not measured			not measured			not measured	0	0	0	no	coverage-limited		no	coverage-limited: comparator data incomplete
IMPG1	interphotoreceptor matrix proteoglycan 1	DOWN	Core matrisome	Proteoglycans	-0.643	4.73×10^{-5}	51.08			not measured			not measured	-0.019	0.9018	measured, low/discordant effect	1	0	0	no	coverage-limited		no	coverage-limited: comparator data incomplete
PCOLCE2	procollagen C-endopeptidase enhancer 2	DOWN	Core matrisome	ECM Glycoproteins	-0.642	6.62×10^{-5}	928	-0.183	0.4941	measured, low/discordant effect	-0.742	0.1412	concordant sub-threshold	0.308	0.7185	measured, low/discordant effect	3	1	1	yes	power-sensitive	-0.0148	no	power-sensitive: 1 comparator(s) concordant sub-threshold
TNFSF12	tumor necrosis factor (ligand) superfamily, member 12	DOWN	Matrisome-associated	Secreted Factors	-0.624	4.11×10^{-17}	2020	-0.216	0.07655	measured, low/discordant effect	-0.351	0.0274	concordant sub-threshold	0.039	0.8459	measured, low/discordant effect	3	1	1	yes	power-sensitive	-0.3192	no	power-sensitive: 1 comparator(s) concordant sub-threshold
AMBP	alpha-1-microglobulin/bikunin precursor	DOWN	Matrisome-associated	ECM Regulators	-0.62	0.003461	5.92			not measured			not measured	-0.125	0.4999	measured, low/discordant effect	1	0	0	no	coverage-limited		no	coverage-limited: comparator data incomplete
ADAMTS2	ADAM metallopeptidase with thrombospondin type 1 motif, 2	DOWN	Matrisome-associated	ECM Regulators	-0.593	6.02×10^{-5}	210.1	-0.026	0.9277	measured, low/discordant effect	0.535	0.02116	measured, low/discordant effect	-0.072	0.815	measured, low/discordant effect	3	0	0	yes	IPF-dominant	0.3423	no	IPF-dominant: no concordant comparator signal
