# Supplementary material for: Shared and Disease-Preferential Matrisome Programs in Fibrosing Interstitial Lung Disease: Evidence from Cross-Cohort and Within-Cohort Analysis
Source: Curr Issues Mol Biol. 2026 Sep 17;48(9):951. doi: 10.3390/cimb48090951 (PMC13605140; doi:10.3390/cimb48090951)
Supplement: Supplementary file 1 [file cimb-48-00951-s001.zip › TableS5_matrisome_enrichment_by_sharing_degree.pdf]

Table S5. Matrisome enrichment by degree of cross-disease sharing, with odds ratios, Woolf 95% confidence intervals, P values and the exact background universe.

direction	sharing_degree	n_genes	n_matrisome	pct	OR	CI_low	CI_high	P	background_universe_genes	background_matrisome
UP	1	4750	204	4.29	0.27	0.22	0.33	1.40×10^{-37}	6369	435
UP	2	899	124	13.79	2.65	2.13	3.31	5.00×10^{-16}	6369	435
UP	3	674	86	12.76	2.24	1.74	2.88	3.53×10^{-9}	6369	435
UP	4	46	21	45.65	11.99	6.65	21.6	2.94×10^{-13}	6369	435
