# Supplementary material for: Shared and Disease-Preferential Matrisome Programs in Fibrosing Interstitial Lung Disease: Evidence from Cross-Cohort and Within-Cohort Analysis
Source: Curr Issues Mol Biol. 2026 Sep 17;48(9):951. doi: 10.3390/cimb48090951 (PMC13605140; doi:10.3390/cimb48090951)
Supplement: Supplementary file 1 [file cimb-48-00951-s001.zip › TableS6a_category_composition_with_CIs.pdf]

Table S6a. Matrisome category composition of the threshold-defined upregulated signatures, with exact counts and Wilson binomial 95% confidence intervals.

disease	category	count	total	percent	CI95_low_pct	CI95_high_pct
HP	Collagens	1	84	1.19	0.21	6.44
HP	ECM Glycoproteins	10	84	11.9	6.6	20.55
HP	ECM Regulators	15	84	17.86	11.13	27.39
HP	ECM-affiliated Proteins	16	84	19.05	12.08	28.72
HP	Proteoglycans	2	84	2.38	0.66	8.27
HP	Secreted Factors	40	84	47.62	37.28	58.17
IPF	Collagens	2	64	3.12	0.86	10.7
IPF	ECM Glycoproteins	10	64	15.62	8.71	26.43
IPF	ECM Regulators	17	64	26.56	17.3	38.48
IPF	ECM-affiliated Proteins	13	64	20.31	12.27	31.71
IPF	Proteoglycans	2	64	3.12	0.86	10.7
IPF	Secreted Factors	20	64	31.25	21.23	43.39
Non-IPF ILD	Collagens	2	36	5.56	1.54	18.14
Non-IPF ILD	ECM Glycoproteins	11	36	30.56	18	46.86
Non-IPF ILD	ECM Regulators	11	36	30.56	18	46.86
Non-IPF ILD	ECM-affiliated Proteins	2	36	5.56	1.54	18.14
Non-IPF ILD	Proteoglycans	1	36	2.78	0.49	14.17
Non-IPF ILD	Secreted Factors	9	36	25	13.75	41.07
SSc-ILD	Collagens	0	8	0	0	32.44
SSc-ILD	ECM Glycoproteins	4	8	50	21.52	78.48
SSc-ILD	ECM Regulators	1	8	12.5	2.24	47.09
SSc-ILD	ECM-affiliated Proteins	1	8	12.5	2.24	47.09
SSc-ILD	Proteoglycans	0	8	0	0	32.44
SSc-ILD	Secreted Factors	2	8	25	7.15	59.07
