# Supplementary material for: Shared and Disease-Preferential Matrisome Programs in Fibrosing Interstitial Lung Disease: Evidence from Cross-Cohort and Within-Cohort Analysis
Source: Curr Issues Mol Biol. 2026 Sep 17;48(9):951. doi: 10.3390/cimb48090951 (PMC13605140; doi:10.3390/cimb48090951)
Supplement: Supplementary file 1 [file cimb-48-00951-s001.zip › TableS6b_category_composition_tests.pdf]

Table S6b. Pairwise contingency tests of matrisome category composition against IPF.

comparison	chi2	P	min_expected
IPF vs HP	4.82	0.4382	1.3
IPF vs Non-IPF ILD	6.58	0.2536	1.08
IPF vs SSc-ILD	5.7	0.3367	0.22
