# Supplementary material for: Shared and Disease-Preferential Matrisome Programs in Fibrosing Interstitial Lung Disease: Evidence from Cross-Cohort and Within-Cohort Analysis
Source: Curr Issues Mol Biol. 2026 Sep 17;48(9):951. doi: 10.3390/cimb48090951 (PMC13605140; doi:10.3390/cimb48090951)
Supplement: Supplementary file 1 [file cimb-48-00951-s001.zip › TableS7_leave_one_out_enrichment.pdf]

**Table S7. Leave-one-cohort-out robustness of the matrisome enrichment. Each disease arm is removed in turn and both the enrichment odds ratio at maximum sharing degree and the Cochran-Armitage trend are re-estimated against the recomputed background universe.**

| analysis | arms_retained | sharing_degree | n_genes | n_matrisome | pct_matrisome | OR | CI_low | CI_high | P | background_universe_genes | background_matrisome | background_pct | trend_Z | trend_P |
|---|---|---|---|---|---|---|---|---|---|---|---|---|---|---|
| Full analysis (all four arms) | 4 | 4 | 46 | 21 | 45.65 | 11.99 | 6.65 | 21.6 | $2.94 \times 10^{-13}$ | 6369 | 435 | 6.83 | 13.93 | $4.35 \times 10^{-44}$ |
| Leave out IPF | 3 | 3 | 52 | 23 | 44.23 | 11.78 | 6.74 | 20.59 | $2.66 \times 10^{-14}$ | 5539 | 369 | 6.66 | 14.82 | $1.02 \times 10^{-49}$ |
| Leave out HP | 3 | 3 | 63 | 27 | 42.86 | 7.07 | 4.24 | 11.8 | $1.06 \times 10^{-11}$ | 3368 | 344 | 10.21 | 7.78 | $7.50 \times 10^{-15}$ |
| Leave out Non-IPF ILD | 3 | 3 | 57 | 22 | 38.6 | 9.22 | 5.35 | 15.87 | $3.27 \times 10^{-12}$ | 5931 | 397 | 6.69 | 10.55 | $4.94 \times 10^{-26}$ |
| Leave out SSc-ILD | 3 | 3 | 686 | 98 | 14.29 | 2.48 | 1.95 | 3.15 | $5.80 \times 10^{-12}$ | 5888 | 426 | 7.24 | 11.6 | $4.01 \times 10^{-31}$ |
