# Supplementary material for: Shared and Disease-Preferential Matrisome Programs in Fibrosing Interstitial Lung Disease: Evidence from Cross-Cohort and Within-Cohort Analysis
Source: Curr Issues Mol Biol. 2026 Sep 17;48(9):951. doi: 10.3390/cimb48090951 (PMC13605140; doi:10.3390/cimb48090951)
Supplement: Supplementary file 1 [file cimb-48-00951-s001.zip › TableS8_within_cohort_IPF_vs_CHP.pdf]

Table S8. Within-cohort comparison of IPF and chronic hypersensitivity pneumonitis in GSE150910 (103 IPF versus 82 chronic HP), per-gene results for the 101 candidate genes.

gene_symbol	direction	matrisome_category	IPF_log2FC	evidence_class	IPFvsCHP_log2FC	IPFvsCHP_lfcSE	IPFvsCHP_p	IPFvsCHP_padj	testable_in_GSE150910	IPF_preferential_vs_CHP
LGALS7	UP	ECM-affiliated Proteins	3.153	coverage-limited	-1.212	0.3601	7.61×10^{-4}	0.002119	True	False
MMP3	UP	ECM Regulators	2.544	coverage-limited	-0.1074	0.4401	0.8071	0.849	True	False
IL13	UP	Secreted Factors	2.351	coverage-limited	1.896	0.3007	2.91×10^{-10}	3.53×10^{-9}	True	True
BMP15	UP	Secreted Factors	2.118	coverage-limited	0.6858	0.308	0.02595	0.04754	True	True
DMP1	UP	ECM Glycoproteins	2.057	coverage-limited	0.04697	0.3208	0.8836	0.9109	True	False
MMP12	UP	ECM Regulators	1.988	power-sensitive	0.4701	0.2833	0.09706	0.1495	True	False
PRSS1	UP	ECM Regulators	1.983	coverage-limited	0.1303	0.2807	0.6426	0.712	True	False
C1QTNF8	UP	ECM-affiliated Proteins	1.952	coverage-limited	0.3283	0.3204	0.3055	0.3915	True	False
SERPINA6	UP	ECM Regulators	1.914	coverage-limited	1.009	0.4416	0.02235	0.04175	True	True
ITIH6	UP	ECM Regulators	1.897	coverage-limited	0.9928	0.4355	0.02261	0.0422	True	True
MUCL1	UP	ECM-affiliated Proteins	1.861	coverage-limited	0.9232	0.4069	0.02326	0.04318	True	True
CCL7	UP	Secreted Factors	1.749	coverage-limited	0.7679	0.289	0.007882	0.01683	True	True
ZPLD1	UP	ECM Glycoproteins	1.631	coverage-limited	0.4662	0.2504	0.06265	0.1023	True	False
ADAMDEC1	UP	ECM Regulators	1.523	power-sensitive	-1.243	0.277	7.27×10^{-6}	3.33×10^{-5}	True	False
NELL1	UP	ECM Glycoproteins	1.492	coverage-limited	0.3548	0.2118	0.09389	0.1452	True	False
WNT4	UP	Secreted Factors	1.483	power-sensitive	-0.1508	0.1565	0.3351	0.4227	True	False
TNFSF11	UP	Secreted Factors	1.474	coverage-limited	0.2689	0.2073	0.1947	0.269	True	False
KERA	UP	Proteoglycans	1.474	coverage-limited	0.5207	0.1737	0.002717	0.00659	True	False
ANXA10	UP	ECM-affiliated Proteins	1.367	coverage-limited	0.04808	0.2178	0.8253	0.8647	True	False
ITLN1	UP	ECM-affiliated Proteins	1.339	coverage-limited	-1.407	0.327	1.68×10^{-5}	7.12×10^{-5}	True	False
CLEC2L	UP	ECM-affiliated Proteins	1.302	coverage-limited	0.4156	0.3307	0.2089	0.2847	True	False
CCL1	UP	Secreted Factors	1.274	coverage-limited	-0.9953	0.2903	6.07×10^{-4}	0.001736	True	False
ADAMTS19	UP	ECM Regulators	1.271	coverage-limited	0.4157	0.2826	0.1413	0.2051	True	False
CLEC4G	UP	ECM-affiliated Proteins	1.258	coverage-limited	0.2933	0.2138	0.1702	0.2401	True	False
ITIH1	UP	ECM Regulators	1.245	coverage-limited	-0.1151	0.1462	0.4313	0.5187	True	False
VWC2L	UP	Secreted Factors	1.238	coverage-limited	0.7951	0.2991	0.00786	0.01679	True	True
SERPINB7	UP	ECM Regulators	1.156	coverage-limited	1.078	0.286	1.65×10^{-4}	5.45×10^{-4}	True	True
COL11A1	UP	Collagens	1.155	coverage-limited	0.3178	0.2671	0.234	0.3131	True	False
MEPE	UP	ECM Glycoproteins	1.131	coverage-limited	0.9238	0.2887	0.001372	0.003579	True	True
INSL5	UP	Secreted Factors	1.101	coverage-limited	0.1835	0.2547	0.4712	0.5567	True	False
ELFN1	UP	ECM-affiliated Proteins	1.062	coverage-limited	0.13	0.137	0.3426	0.4301	True	False
C1QTNF9	UP	ECM-affiliated Proteins	1.038	coverage-limited	-0.04098	0.1816	0.8215	0.8617	True	False
CBLN1	UP	Secreted Factors	1.025	coverage-limited	0.24	0.3806	0.5283	0.6103	True	False
VWA5B2	UP	ECM Glycoproteins	0.978	coverage-limited	0.1246	0.1529	0.4152	0.5028	True	False
S100A11	UP	Secreted Factors	0.949	power-sensitive	-0.03862	0.181	0.8311	0.8694	True	False
SEMA6B	UP	ECM-affiliated Proteins	0.942	IPF-dominant	1.694	0.2501	1.25×10^{-11}	1.98×10^{-10}	True	True
TLL2	UP	ECM Regulators	0.926	coverage-limited	-0.1403	0.1844	0.4466	0.5334	True	False
C1QTNF9B	UP	Secreted Factors	0.913	coverage-limited	0.1861	0.1532	0.2245	0.3025	True	False
IL1RN	UP	Secreted Factors	0.888	power-sensitive	0.7277	0.1866	9.65×10^{-5}	3.37×10^{-4}	True	True
CCL18	UP	Secreted Factors	0.864	power-sensitive	-0.6324	0.1795	4.27×10^{-4}	0.001272	True	False
ST14	UP	ECM Regulators	0.847	power-sensitive	1.052	0.1787	4.00×10^{-9}	3.84×10^{-8}	True	True
ADAMTS3	UP	ECM Regulators	0.839	power-sensitive	0.7374	0.2717	0.006657	0.01455	True	True
RSPO3	UP	ECM Glycoproteins	0.809	coverage-limited	0.5629	0.1826	0.002048	0.00512	True	False
COL9A1	UP	Collagens	0.791	coverage-limited	0.804	0.2165	2.04×10^{-4}	6.57×10^{-4}	True	True
CLEC19A	UP	ECM-affiliated Proteins	0.783	coverage-limited	0.2843	0.1907	0.1361	0.1989	True	False
IL4	UP	Secreted Factors	0.773	coverage-limited	-0.0558	0.2089	0.7894	0.8355	True	False
FLG2	UP	Secreted Factors	0.768	coverage-limited	-0.1042	0.1844	0.572	0.6499	True	False
OTOL1	UP	ECM Glycoproteins	0.765	coverage-limited	0.7484	0.2465	0.002401	0.005894	True	True
CTSG	UP	ECM Regulators	0.748	power-sensitive	0.5036	0.2814	0.07359	0.1177	True	False
CCL16	UP	Secreted Factors	0.715	power-sensitive	0.7233	0.2395	0.002528	0.006173	True	True
S100P	UP	Secreted Factors	0.703	power-sensitive	0.2815	0.2148	0.19	0.2634	True	False
WNT1	UP	Secreted Factors	0.692	coverage-limited	0.3699	0.1632	0.02344	0.04347	True	False
MUC3A	UP	ECM-affiliated Proteins	0.69	coverage-limited	0.1068	0.1721	0.535	0.6164	True	False

gene_symbol	direction	matrisome_category	IPF_log2FC	evidence_class	IPFvsCHP_log2FC	IPFvsCHP_lfcSE	IPFvsCHP_p	IPFvsCHP_padj	testable_in_GSE150910	IPF_preferential_vs_CHP
TNFSF18	UP	Secreted Factors	0.679	coverage-limited	0.3404	0.1587	0.03195	0.05682	True	False
ADAMTS4	UP	ECM Regulators	0.677	coverage-limited	1.969	0.3029	7.95×10^{-11}	1.09×10^{-9}	True	True
VWDE	UP	ECM Glycoproteins	0.676	coverage-limited	-0.3321	0.1718	0.05328	0.08892	True	False
TGM5	UP	ECM Regulators	0.667	coverage-limited	-0.08108	0.1752	0.6435	0.7127	True	False
ANXA8	UP	ECM-affiliated Proteins	0.664	power-sensitive	-0.06184	0.189	0.7436	0.7985	True	False
MSTN	UP	Secreted Factors	0.658	coverage-limited	0.3921	0.1351	0.003706	0.008679	True	False
PRSS3	UP	ECM Regulators	0.618	coverage-limited	0.05265	0.247	0.8312	0.8694	True	False
HSPG2	UP	Proteoglycans	0.609	power-sensitive	-0.2992	0.1191	0.01201	0.02433	True	False
KY	UP	ECM Regulators	0.593	coverage-limited	0.5809	0.1475	8.22×10^{-5}	2.92×10^{-4}	True	False
VWA1	UP	ECM Glycoproteins	0.592	power-sensitive	-0.329	0.07893	3.06×10^{-5}	1.21×10^{-4}	True	False
VWA2	UP	ECM Glycoproteins	0.587	coverage-limited	1.034	0.158	5.89×10^{-11}	8.25×10^{-10}	True	True
IL17A	DOWN	Secreted Factors	-2.334	coverage-limited	-0.4182	0.4865	0.39	0.4777	True	False
MMP8	DOWN	ECM Regulators	-1.806	coverage-limited	0.408	0.3654	0.2641	0.3463	True	False
ADAM29	DOWN	ECM Regulators	-1.492	coverage-limited	0.3446	0.1445	0.01706	0.033	True	False
SEMA3G	DOWN	ECM-affiliated Proteins	-1.27	power-sensitive	-0.1674	0.1715	0.3289	0.4164	True	False
LEFTY2	DOWN	Secreted Factors	-1.153	coverage-limited	-0.5836	0.1662	4.47×10^{-4}	0.001326	True	False
FGL1	DOWN	ECM Glycoproteins	-1.119	coverage-limited	0.5462	0.2022	0.006912	0.01502	True	False
CLEC12B	DOWN	ECM-affiliated Proteins	-1.102	coverage-limited	0.1813	0.1797	0.3131	0.3995	True	False
CPAMD8	DOWN	ECM Regulators	-1.072	coverage-limited	-0.5252	0.1041	4.51×10^{-7}	2.77×10^{-6}	True	False
NTNG1	DOWN	ECM Glycoproteins	-1.061	coverage-limited	0.03189	0.1514	0.8332	0.8709	True	False
RSPO1	DOWN	ECM Glycoproteins	-1.05	coverage-limited	-0.2201	0.1564	0.1594	0.2267	True	False
REG4	DOWN	ECM-affiliated Proteins	-1.042	coverage-limited	0.6667	0.4626	0.1495		False	False
INHBA	DOWN	Secreted Factors	-1.029	power-sensitive	1.14	0.2034	2.06×10^{-8}	1.70×10^{-7}	True	False
CLEC14A	DOWN	ECM-affiliated Proteins	-1.029	coverage-limited	-0.464	0.1008	4.16×10^{-6}	2.02×10^{-5}	True	False
HYAL2	DOWN	ECM Regulators	-1.02	power-sensitive	0.195	0.102	0.05584	0.09256	True	False
TIMP3	DOWN	ECM Regulators	-0.961	power-sensitive	0.1208	0.1122	0.2815	0.3655	True	False
CLEC9A	DOWN	ECM-affiliated Proteins	-0.922	coverage-limited	0.4802	0.177	0.006662	0.01456	True	False
CCN3	DOWN	ECM Glycoproteins	-0.899	coverage-limited	-0.1874	0.3317	0.5721	0.6499	True	False
LAMB4	DOWN	ECM Glycoproteins	-0.875	coverage-limited	-0.5931	0.1582	1.78×10^{-4}	5.81×10^{-4}	True	True
COL6A6	DOWN	Collagens	-0.845	coverage-limited	0.1665	0.1603	0.2989	0.3846	True	False
CELA2B	DOWN	ECM Regulators	-0.84	coverage-limited	0.4517	0.1497	0.002546	0.006215	True	False
CTSW	DOWN	ECM Regulators	-0.803	IPF-dominant	-0.604	0.1647	2.46×10^{-4}	7.78×10^{-4}	True	True
ZP2	DOWN	ECM Glycoproteins	-0.795	coverage-limited	0.453	0.1908	0.01758	0.0339	True	False
GPC3	DOWN	ECM-affiliated Proteins	-0.788	coverage-limited	0.02499	0.1146	0.8274	0.8663	True	False
GDF2	DOWN	Secreted Factors	-0.777	coverage-limited	0.7425	0.3065	0.01541	0.03021	True	False
ADAMTSL3	DOWN	ECM Regulators	-0.728	power-sensitive	0.4164	0.1157	3.19×10^{-4}	9.84×10^{-4}	True	False
FBLN5	DOWN	ECM Glycoproteins	-0.721	power-sensitive	-0.09344	0.09152	0.3073	0.3934	True	False
FGG	DOWN	ECM Glycoproteins	-0.71	power-sensitive	0.4931	0.3399	0.1469	0.2119	True	False
WFIKKN2	DOWN	Secreted Factors	-0.705	coverage-limited	0.1653	0.1451	0.2546	0.3355	True	False
FRAS1	DOWN	ECM Glycoproteins	-0.7	IPF-dominant	0.1559	0.174	0.3704	0.4577	True	False
FGF12	DOWN	Secreted Factors	-0.667	power-sensitive	-0.003418	0.1126	0.9758	0.9821	True	False
ANXA1	DOWN	ECM-affiliated Proteins	-0.664	IPF-dominant	0.8882	0.1275	3.28×10^{-12}	5.75×10^{-11}	True	False
FREM3	DOWN	ECM-affiliated Proteins	-0.663	coverage-limited	-0.3871	0.2194	0.07764	0.1231	True	False
IMPG1	DOWN	Proteoglycans	-0.643	coverage-limited	0.6811	0.1551	1.13×10^{-5}	4.97×10^{-5}	True	False
PCOLCE2	DOWN	ECM Glycoproteins	-0.642	power-sensitive	0.0737	0.1694	0.6636	0.7306	True	False
TNFSF12	DOWN	Secreted Factors	-0.624	power-sensitive	-0.1291	0.09021	0.1522	0.2182	True	False
AMBP	DOWN	ECM Regulators	-0.62	coverage-limited	-0.3167	0.2726	0.2454	0.3256	True	False
ADAMTS2	DOWN	ECM Regulators	-0.593	IPF-dominant	0.4221	0.1307	0.001238	0.003269	True	False
