# Supplementary material for: Shared and Disease-Preferential Matrisome Programs in Fibrosing Interstitial Lung Disease: Evidence from Cross-Cohort and Within-Cohort Analysis
Source: Curr Issues Mol Biol. 2026 Sep 17;48(9):951. doi: 10.3390/cimb48090951 (PMC13605140; doi:10.3390/cimb48090951)
Supplement: Supplementary file 1 [file cimb-48-00951-s001.zip › TableS9_matrisome_genes_IPF_vs_CHP.pdf]

Table S9. Matrisome genes separating IPF from chronic hypersensitivity pneumonitis within GSE150910 (205 genes at adjusted P < 0.05 and |log2FC| >= 0.585).

gene_symbol	baseMean	IPFvsCHP_log2FC	IPFvsCHP_lfcSE	stat	IPFvsCHP_p	IPFvsCHP_padj	g	is_mat	sig
BGN	2057	-3.459	0.2729	-12.67	8.37×10^{-37}	7.38×10^{-33}	BGN	True	True
CXCL9	2465	-2.604	0.253	-10.29	7.68×10^{-25}	2.46×10^{-22}	CXCL9	True	True
MMP13	24.34	2.59	0.3506	7.387	1.50×10^{-13}	3.45×10^{-12}	MMP13	True	True
CLEC3B	130.7	-2.291	0.3268	-7.009	2.39×10^{-12}	4.32×10^{-11}	CLEC3B	True	True
CXCL11	374.7	-2.14	0.2707	-7.906	2.65×10^{-15}	8.59×10^{-14}	CXCL11	True	True
LIF	139.9	2.125	0.2631	8.075	6.77×10^{-16}	2.59×10^{-14}	LIF	True	True
CXCL6	22.88	2.068	0.3808	5.431	5.59×10^{-8}	4.21×10^{-7}	CXCL6	True	True
ADAMTS4	801.4	1.969	0.3029	6.502	7.95×10^{-11}	1.09×10^{-9}	ADAMTS4	True	True
IL13	39.55	1.896	0.3007	6.303	2.91×10^{-10}	3.53×10^{-9}	IL13	True	True
LGI3	145.3	-1.756	0.2977	-5.899	3.66×10^{-9}	3.55×10^{-8}	LGI3	True	True
CXCL8	419.5	1.741	0.2437	7.144	9.10×10^{-13}	1.80×10^{-11}	CXCL8	True	True
SEMA6B	140.1	1.694	0.2501	6.774	1.25×10^{-11}	1.98×10^{-10}	SEMA6B	True	True
IL6	1770	1.638	0.2643	6.197	5.77×10^{-10}	6.60×10^{-9}	IL6	True	True
S100A14	611.6	-1.611	0.1519	-10.61	2.79×10^{-26}	1.33×10^{-23}	S100A14	True	True
ITLN2	208	-1.562	0.3638	-4.294	1.76×10^{-5}	7.40×10^{-5}	ITLN2	True	True
LAMC3	608.4	-1.546	0.2128	-7.264	3.77×10^{-13}	8.02×10^{-12}	LAMC3	True	True
ADAMTS1	691.5	1.541	0.1943	7.933	2.14×10^{-15}	7.14×10^{-14}	ADAMTS1	True	True
CCN4	30.51	1.508	0.2972	5.074	3.90×10^{-7}	2.44×10^{-6}	CCN4	True	True
FSTL1	617.1	1.465	0.1777	8.244	1.66×10^{-16}	7.15×10^{-15}	FSTL1	True	True
OSM	51.6	1.443	0.2566	5.625	1.86×10^{-8}	1.55×10^{-7}	OSM	True	True
MUC5AC	94.66	1.419	0.4029	3.523	4.26×10^{-4}	0.00127	MUC5AC	True	True
ITLN1	44.42	-1.407	0.327	-4.303	1.68×10^{-5}	7.12×10^{-5}	ITLN1	True	True
TNFAIP6	47.78	1.383	0.3265	4.236	2.27×10^{-5}	9.30×10^{-5}	TNFAIP6	True	True
COL4A1	87.42	1.382	0.2266	6.098	1.07×10^{-9}	1.16×10^{-8}	COL4A1	True	True
CST1	176.7	-1.333	0.4098	-3.253	0.001142	0.003037	CST1	True	True
COL5A3	27.64	1.302	0.2018	6.453	1.10×10^{-10}	1.46×10^{-9}	COL5A3	True	True
IL11	74.63	1.294	0.2225	5.817	6.00×10^{-9}	5.52×10^{-8}	IL11	True	True
CST3	1.573×10^{4}	-1.25	0.1616	-7.737	1.02×10^{-14}	2.94×10^{-13}	CST3	True	True
ADAMDEC1	270.7	-1.243	0.277	-4.486	7.27×10^{-6}	3.33×10^{-5}	ADAMDEC1	True	True
TIMP1	2.197×10^{4}	-1.231	0.2042	-6.029	1.65×10^{-9}	1.72×10^{-8}	TIMP1	True	True
PZP	51.45	1.229	0.222	5.538	3.06×10^{-8}	2.44×10^{-7}	PZP	True	True
COL17A1	310.2	-1.227	0.226	-5.427	5.72×10^{-8}	4.29×10^{-7}	COL17A1	True	True
LGALS7	428.9	-1.212	0.3601	-3.366	7.61×10^{-4}	0.002119	LGALS7	True	True
AMELX	1.365	1.2	0.4688	2.56	0.01046	0.02165	AMELX	True	True
CLEC6A	16.62	-1.196	0.2501	-4.783	1.72×10^{-6}	9.19×10^{-6}	CLEC6A	True	True
SEMA3C	192.1	1.175	0.2378	4.941	7.77×10^{-7}	4.50×10^{-6}	SEMA3C	True	True
GREM1	265.1	1.172	0.2531	4.631	3.65×10^{-6}	1.81×10^{-5}	GREM1	True	True
C1QA	1.587×10^{4}	-1.159	0.1433	-8.09	5.96×10^{-16}	2.33×10^{-14}	C1QA	True	True
FGB	8.075	1.154	0.4294	2.688	0.007178	0.01551	FGB	True	True
INHBA	34.08	1.14	0.2034	5.607	2.06×10^{-8}	1.70×10^{-7}	INHBA	True	True
AREG	95.48	1.138	0.2361	4.819	1.44×10^{-6}	7.84×10^{-6}	AREG	True	True
SERPINB11	61.26	1.135	0.3555	3.194	0.001405	0.003655	SERPINB11	True	True
SEMA3A	23.02	1.133	0.2537	4.468	7.89×10^{-6}	3.59×10^{-5}	SEMA3A	True	True
VWA3A	437.2	1.12	0.2513	4.456	8.36×10^{-6}	3.78×10^{-5}	VWA3A	True	True
CCN1	460.8	1.109	0.1913	5.8	6.63×10^{-9}	6.05×10^{-8}	CCN1	True	True
ANXA11	9111	-1.102	0.1022	-10.78	4.33×10^{-27}	2.55×10^{-24}	ANXA11	True	True
COL24A1	7.213	1.101	0.2129	5.171	2.32×10^{-7}	1.53×10^{-6}	COL24A1	True	True
ADAM12	130.8	1.1	0.1701	6.467	9.97×10^{-11}	1.34×10^{-9}	ADAM12	True	True
CCL24	1070	1.099	0.2208	4.978	6.44×10^{-7}	3.82×10^{-6}	CCL24	True	True
PAPPA2	7.113	1.079	0.4095	2.634	0.00843	0.01789	PAPPA2	True	True
MMP25	161	1.078	0.1863	5.787	7.17×10^{-9}	6.49×10^{-8}	MMP25	True	True
SERPINB7	18.92	1.078	0.286	3.767	1.65×10^{-4}	5.45×10^{-4}	SERPINB7	True	True
HBEGF	57.69	1.056	0.2025	5.215	1.83×10^{-7}	1.23×10^{-6}	HBEGF	True	True
ST14	27.84	1.052	0.1787	5.884	4.00×10^{-9}	3.84×10^{-8}	ST14	True	True
IGFBP1	1.62	-1.048	0.4736	-2.212	0.02697	0.04915	IGFBP1	True	True
VWA2	16.95	1.034	0.158	6.546	5.89×10^{-11}	8.25×10^{-10}	VWA2	True	True
SNED1	239.9	1.03	0.1095	9.406	5.17×10^{-21}	6.20×10^{-19}	SNED1	True	True
COL4A2	198.2	1.026	0.1562	6.567	5.13×10^{-11}	7.24×10^{-10}	COL4A2	True	True
IL1B	683.8	1.017	0.2205	4.611	4.01×10^{-6}	1.95×10^{-5}	IL1B	True	True
MUC1	4.032×10^{4}	-1.016	0.1213	-8.372	5.65×10^{-17}	2.70×10^{-15}	MUC1	True	True
SERPINA6	4.06	1.009	0.4416	2.284	0.02235	0.04175	SERPINA6	True	True
SPON1	60.56	0.9986	0.1285	7.773	7.68×10^{-15}	2.30×10^{-13}	SPON1	True	True
MMRN1	328	0.9969	0.2139	4.661	3.14×10^{-6}	1.58×10^{-5}	MMRN1	True	True
CCL1	13.99	-0.9953	0.2903	-3.428	6.07×10^{-4}	0.001736	CCL1	True	True
ITIH6	2.186	0.9928	0.4355	2.28	0.02261	0.0422	ITIH6	True	True
EGLN2	2357	-0.9866	0.1043	-9.463	2.99×10^{-21}	4.06×10^{-19}	EGLN2	True	True

gene_symbol	baseMean	IPFvsCHP_log2FC	IPFvsCHP_lfcSE	stat	IPFvsCHP_p	IPFvsCHP_padj	g	is_mat	sig
CRISPLD2	240.7	0.9848	0.1962	5.019	5.20×10^{-7}	3.15×10^{-6}	CRISPLD2	True	True
MUC5B	2686	0.9788	0.3095	3.162	0.001565	0.004021	MUC5B	True	True
RSPO4	27.14	0.9701	0.2121	4.574	4.79×10^{-6}	2.29×10^{-5}	RSPO4	True	True
PXDN	92.89	0.9688	0.1278	7.578	3.52×10^{-14}	9.02×10^{-13}	PXDN	True	True
NID1	235.8	-0.9673	0.2651	-3.648	2.64×10^{-4}	8.29×10^{-4}	NID1	True	True
CCL13	88.5	-0.961	0.3071	-3.129	0.001751	0.004456	CCL13	True	True
THBS1	9017	0.9556	0.2026	4.715	2.41×10^{-6}	1.24×10^{-5}	THBS1	True	True
EREG	22.5	0.9544	0.1975	4.833	1.35×10^{-6}	7.38×10^{-6}	EREG	True	True
LMAN1	152.6	0.9525	0.1236	7.706	1.30×10^{-14}	3.66×10^{-13}	LMAN1	True	True
INHA	5.919	0.9523	0.3031	3.142	0.001676	0.00428	INHA	True	True
AEBP1	4435	-0.9414	0.1603	-5.873	4.28×10^{-9}	4.08×10^{-8}	AEBP1	True	True
LRG1	89.62	0.9403	0.2034	4.624	3.77×10^{-6}	1.85×10^{-5}	LRG1	True	True
COL3A1	2.386×10^{4}	0.9338	0.1741	5.364	8.13×10^{-8}	5.91×10^{-7}	COL3A1	True	True
FGF7	1312	0.9268	0.2028	4.57	4.88×10^{-6}	2.32×10^{-5}	FGF7	True	True
MEPE	5.425	0.9238	0.2887	3.2	0.001372	0.003579	MEPE	True	True
MUCL1	23.79	0.9232	0.4069	2.269	0.02326	0.04318	MUCL1	True	True
CLEC10A	843.8	-0.9156	0.1556	-5.883	4.03×10^{-9}	3.86×10^{-8}	CLEC10A	True	True
VEGFC	15.05	0.9109	0.1933	4.712	2.45×10^{-6}	1.26×10^{-5}	VEGFC	True	True
HABP2	29.95	-0.9079	0.2866	-3.168	0.001533	0.003947	HABP2	True	True
ANXA1	2399	0.8882	0.1275	6.965	3.28×10^{-12}	5.75×10^{-11}	ANXA1	True	True
COLEC10	3.211	0.8859	0.2737	3.236	0.00121	0.003203	COLEC10	True	True
IL3	0.896	0.8843	0.4005	2.208	0.02726	0.04958	IL3	True	True
SFTPC	7.80×10^{5}	-0.877	0.219	-4.005	6.19×10^{-5}	2.27×10^{-4}	SFTPC	True	True
LOX	111.4	0.8765	0.2036	4.305	1.67×10^{-5}	7.06×10^{-5}	LOX	True	True
LTB	1838	-0.8702	0.1574	-5.527	3.26×10^{-8}	2.58×10^{-7}	LTB	True	True
ANXA6	2842	-0.8639	0.1246	-6.935	4.07×10^{-12}	6.92×10^{-11}	ANXA6	True	True
CTSD	4.123×10^{4}	-0.8634	0.1211	-7.132	9.88×10^{-13}	1.95×10^{-11}	CTSD	True	True
TDGF1	10.46	0.8536	0.2438	3.502	4.62×10^{-4}	0.001364	TDGF1	True	True
F2	0.9637	0.8405	0.302	2.783	0.005388	0.01209	F2	True	True
COL1A1	1.523×10^{4}	0.8282	0.1824	4.541	5.60×10^{-6}	2.63×10^{-5}	COL1A1	True	True
CCL25	13.82	-0.8263	0.2463	-3.355	7.93×10^{-4}	0.002193	CCL25	True	True
SEMA7A	62.76	0.82	0.1696	4.835	1.33×10^{-6}	7.31×10^{-6}	SEMA7A	True	True
IGFALS	47.22	-0.8194	0.1364	-6.008	1.88×10^{-9}	1.93×10^{-8}	IGFALS	True	True
ADAMTS7	80.88	0.8188	0.1461	5.603	2.11×10^{-8}	1.74×10^{-7}	ADAMTS7	True	True
LGALS4	57.93	-0.8054	0.1423	-5.659	1.52×10^{-8}	1.29×10^{-7}	LGALS4	True	True
SERPINB5	10.68	0.8042	0.3415	2.355	0.01852	0.03548	SERPINB5	True	True
COL9A1	4.784	0.804	0.2165	3.714	2.04×10^{-4}	6.57×10^{-4}	COL9A1	True	True
SERPINB1	1007	0.8034	0.1512	5.312	1.09×10^{-7}	7.65×10^{-7}	SERPINB1	True	True
MASP2	51.87	0.8028	0.1276	6.293	3.11×10^{-10}	3.74×10^{-9}	MASP2	True	True
IFNW1	3.225	0.8019	0.1527	5.251	1.51×10^{-7}	1.03×10^{-6}	IFNW1	True	True
ADAM17	3.958	0.8007	0.2823	2.836	0.004567	0.01044	ADAM17	True	True
VWC2L	1.671	0.7951	0.2991	2.658	0.00786	0.01679	VWC2L	True	True
CTSK	58.94	0.7947	0.1651	4.812	1.49×10^{-6}	8.09×10^{-6}	CTSK	True	True
C1QB	2.587×10^{4}	-0.789	0.1517	-5.202	1.97×10^{-7}	1.31×10^{-6}	C1QB	True	True
CBLN4	119.8	0.7876	0.2206	3.571	3.56×10^{-4}	0.001084	CBLN4	True	True
CCL27	6.154	-0.786	0.2901	-2.709	0.006746	0.01472	CCL27	True	True
MMP14	149	0.7781	0.1158	6.718	1.84×10^{-11}	2.81×10^{-10}	MMP14	True	True
FLT3LG	682	-0.7778	0.0823	-9.451	3.36×10^{-21}	4.49×10^{-19}	FLT3LG	True	True
COL5A1	2326	0.7711	0.1403	5.496	3.89×10^{-8}	3.04×10^{-7}	COL5A1	True	True
CRISPLD1	108.4	0.7705	0.1806	4.267	1.98×10^{-5}	8.23×10^{-5}	CRISPLD1	True	True
CCL7	220	0.7679	0.289	2.657	0.007882	0.01683	CCL7	True	True
VCAN	2704	0.7668	0.1358	5.645	1.65×10^{-8}	1.40×10^{-7}	VCAN	True	True
ZP3	269.7	-0.7631	0.1649	-4.628	3.68×10^{-6}	1.82×10^{-5}	ZP3	True	True
ASTL	14.52	0.7625	0.1994	3.823	1.32×10^{-4}	4.45×10^{-4}	ASTL	True	True
PLAU	104.7	0.7618	0.1578	4.827	1.39×10^{-6}	7.57×10^{-6}	PLAU	True	True
CD109	28.28	0.7611	0.1737	4.382	1.18×10^{-5}	5.16×10^{-5}	CD109	True	True
CTHRC1	484.6	0.758	0.139	5.455	4.89×10^{-8}	3.74×10^{-7}	CTHRC1	True	True
CLEC11A	132.8	-0.7517	0.2671	-2.815	0.004881	0.01109	CLEC11A	True	True
OTOL1	2.276	0.7484	0.2465	3.036	0.002401	0.005894	OTOL1	True	True
GDF2	1.423	0.7425	0.3065	2.423	0.01541	0.03021	GDF2	True	True
SERPINF1	8372	-0.7375	0.1105	-6.674	2.49×10^{-11}	3.72×10^{-10}	SERPINF1	True	True
ADAMTS3	5.405	0.7374	0.2717	2.714	0.006657	0.01455	ADAMTS3	True	True
MMP19	1924	0.729	0.1998	3.648	2.64×10^{-4}	8.30×10^{-4}	MMP19	True	True
SERPINE1	5754	0.7285	0.2468	2.952	0.00316	0.007538	SERPINE1	True	True
MATN4	8.968	-0.7283	0.1776	-4.101	4.11×10^{-5}	1.58×10^{-4}	MATN4	True	True
IL1RN	2203	0.7277	0.1866	3.899	9.65×10^{-5}	3.37×10^{-4}	IL1RN	True	True
POSTN	8688	0.7268	0.1419	5.121	3.05×10^{-7}	1.95×10^{-6}	POSTN	True	True
SCUBE1	61.82	0.7246	0.1713	4.23	2.34×10^{-5}	9.53×10^{-5}	SCUBE1	True	True

gene_symbol	baseMean	IPFvsCHP_log2FC	IPFvsCHP_lfcSE	stat	IPFvsCHP_p	IPFvsCHP_padj	g	is_mat	sig
TGFA	24.51	0.7239	0.202	3.583	3.39×10^{-4}	0.001039	TGFA	True	True
CLEC5A	96.79	0.7239	0.1593	4.543	5.53×10^{-6}	2.60×10^{-5}	CLEC5A	True	True
CCL16	16.06	0.7233	0.2395	3.02	0.002528	0.006173	CCL16	True	True
SPP1	2440	0.7228	0.2703	2.674	0.007496	0.01612	SPP1	True	True
LAMA2	811.2	0.7167	0.1093	6.56	5.39×10^{-11}	7.59×10^{-10}	LAMA2	True	True
ADAMTS6	57.73	0.7159	0.142	5.043	4.59×10^{-7}	2.82×10^{-6}	ADAMTS6	True	True
OGN	53.89	0.7039	0.2004	3.512	4.44×10^{-4}	0.001319	OGN	True	True
HGF	39.18	0.7034	0.1549	4.542	5.58×10^{-6}	2.62×10^{-5}	HGF	True	True
CBLN2	17.73	0.702	0.1989	3.53	4.16×10^{-4}	0.001245	CBLN2	True	True
FAM20C	976.3	-0.7013	0.1419	-4.942	7.72×10^{-7}	4.48×10^{-6}	FAM20C	True	True
TNC	2962	0.6975	0.1247	5.594	2.22×10^{-8}	1.82×10^{-7}	TNC	True	True
MUC16	528.9	0.6964	0.2859	2.436	0.01487	0.02929	MUC16	True	True
IGFBP5	55.73	0.695	0.1598	4.35	1.36×10^{-5}	5.87×10^{-5}	IGFBP5	True	True
ANGPTL1	328.9	0.6944	0.168	4.133	3.58×10^{-5}	1.39×10^{-4}	ANGPTL1	True	True
LAMC1	309.9	0.6913	0.1228	5.631	1.79×10^{-8}	1.50×10^{-7}	LAMC1	True	True
SFTA2	1835	-0.6884	0.1534	-4.487	7.22×10^{-6}	3.31×10^{-5}	SFTA2	True	True
BRINP3	45.94	0.688	0.1804	3.814	1.37×10^{-4}	4.60×10^{-4}	BRINP3	True	True
ANXA3	1055	-0.6869	0.1386	-4.957	7.14×10^{-7}	4.18×10^{-6}	ANXA3	True	True
CX3CL1	47.29	0.6862	0.1783	3.849	1.19×10^{-4}	4.05×10^{-4}	CX3CL1	True	True
BMP15	5.681	0.6858	0.308	2.227	0.02595	0.04754	BMP15	True	True
COL27A1	281.1	0.6848	0.1124	6.09	1.13×10^{-9}	1.22×10^{-8}	COL27A1	True	True
IMPG1	27.75	0.6811	0.1551	4.391	1.13×10^{-5}	4.97×10^{-5}	IMPG1	True	True
CLEC17A	33.15	0.6806	0.2114	3.22	0.001282	0.00337	CLEC17A	True	True
S100A4	6305	-0.6796	0.09649	-7.043	1.88×10^{-12}	3.46×10^{-11}	S100A4	True	True
CCL20	368.6	0.6783	0.2195	3.09	0.001999	0.005008	CCL20	True	True
CCL17	947.4	-0.6781	0.206	-3.292	9.94×10^{-4}	0.002683	CCL17	True	True
FBN1	2572	0.6766	0.1288	5.252	1.50×10^{-7}	1.03×10^{-6}	FBN1	True	True
WNT7A	49.24	-0.6704	0.1478	-4.537	5.71×10^{-6}	2.67×10^{-5}	WNT7A	True	True
WNT10B	15.55	-0.6699	0.2683	-2.497	0.01254	0.02524	WNT10B	True	True
INSL3	29.76	-0.6684	0.1828	-3.657	2.55×10^{-4}	8.04×10^{-4}	INSL3	True	True
C1QC	1961	-0.6682	0.1471	-4.543	5.55×10^{-6}	2.61×10^{-5}	C1QC	True	True
CXCL1	2301	0.668	0.1984	3.367	7.59×10^{-4}	0.002114	CXCL1	True	True
GH1	5.67	-0.6648	0.2959	-2.246	0.02469	0.0455	GH1	True	True
LTBP2	222.2	0.6589	0.14	4.705	2.53×10^{-6}	1.30×10^{-5}	LTBP2	True	True
IGFBP6	3678	-0.6566	0.1638	-4.009	6.09×10^{-5}	2.24×10^{-4}	IGFBP6	True	True
NRG4	203.5	0.6537	0.163	4.01	6.06×10^{-5}	2.23×10^{-4}	NRG4	True	True
GPC2	35.97	0.6523	0.1078	6.054	1.41×10^{-9}	1.49×10^{-8}	GPC2	True	True
LAMB2	1.184×10^{4}	-0.6498	0.1076	-6.04	1.54×10^{-9}	1.62×10^{-8}	LAMB2	True	True
BTC	16.08	0.6492	0.2113	3.073	0.002122	0.005287	BTC	True	True
IGF1	919.4	0.6487	0.1621	4.002	6.27×10^{-5}	2.30×10^{-4}	IGF1	True	True
FGF14	189.6	0.6456	0.1524	4.235	2.28×10^{-5}	9.33×10^{-5}	FGF14	True	True
PCOLCE	1895	-0.6453	0.1425	-4.528	5.96×10^{-6}	2.78×10^{-5}	PCOLCE	True	True
LTA	55.03	-0.6451	0.1623	-3.974	7.06×10^{-5}	2.55×10^{-4}	LTA	True	True
THBS2	1595	0.6423	0.1557	4.125	3.70×10^{-5}	1.44×10^{-4}	THBS2	True	True
ADAM28	651.4	0.634	0.1355	4.678	2.89×10^{-6}	1.46×10^{-5}	ADAM28	True	True
TLL1	12.68	0.6325	0.2195	2.881	0.003961	0.009202	TLL1	True	True
CCL18	2.338×10^{4}	-0.6324	0.1795	-3.523	4.27×10^{-4}	0.001272	CCL18	True	True
COL4A5	404.6	0.6324	0.1115	5.67	1.43×10^{-8}	1.23×10^{-7}	COL4A5	True	True
A2ML1	15.2	0.6316	0.1256	5.028	4.97×10^{-7}	3.02×10^{-6}	A2ML1	True	True
SEMA3D	133.8	0.6297	0.1272	4.95	7.42×10^{-7}	4.32×10^{-6}	SEMA3D	True	True
COL4A6	147.7	0.6247	0.1467	4.258	2.06×10^{-5}	8.53×10^{-5}	COL4A6	True	True
CCL2	1.955×10^{4}	0.6226	0.2049	3.039	0.002373	0.005834	CCL2	True	True
PLOD1	329.7	-0.6223	0.117	-5.32	1.04×10^{-7}	7.36×10^{-7}	PLOD1	True	True
ITIH4	759	-0.6213	0.1233	-5.039	4.68×10^{-7}	2.87×10^{-6}	ITIH4	True	True
PDGFD	27.69	0.6189	0.1809	3.421	6.23×10^{-4}	0.001775	PDGFD	True	True
IL1A	54.83	0.6169	0.2193	2.813	0.004915	0.01115	IL1A	True	True
EGFL7	8321	-0.6152	0.11	-5.591	2.25×10^{-8}	1.84×10^{-7}	EGFL7	True	True
CXCL3	655.2	0.6151	0.1742	3.531	4.14×10^{-4}	0.00124	CXCL3	True	True
CTSW	105.3	-0.604	0.1647	-3.667	2.46×10^{-4}	7.78×10^{-4}	CTSW	True	True
PLXNA4	130.1	0.6008	0.1377	4.363	1.28×10^{-5}	5.58×10^{-5}	PLXNA4	True	True
COL25A1	39.17	0.5985	0.1795	3.335	8.54×10^{-4}	0.002345	COL25A1	True	True
FGF2	229.2	0.5981	0.1443	4.146	3.39×10^{-5}	1.33×10^{-4}	FGF2	True	True
COL7A1	1871	-0.5977	0.1908	-3.132	0.001734	0.004418	COL7A1	True	True
GPC1	316.7	0.5976	0.1783	3.351	8.05×10^{-4}	0.002225	GPC1	True	True
LAMB4	13.86	-0.5931	0.1582	-3.749	1.78×10^{-4}	5.81×10^{-4}	LAMB4	True	True
SPARC	3.604×10^{4}	0.5922	0.155	3.819	1.34×10^{-4}	4.51×10^{-4}	SPARC	True	True
COL6A3	7242	0.5903	0.1266	4.661	3.14×10^{-6}	1.58×10^{-5}	COL6A3	True	True
SRPX	590.9	-0.5898	0.1685	-3.5	4.65×10^{-4}	0.00137	SRPX	True	True

gene_symbol	baseMean	IPFvsCHP_log2FC	IPFvsCHP_lfcSE	stat	IPFvsCHP_p	IPFvsCHP_padj	g	is_mat	sig
COL14A1	3189	0.5889	0.1444	4.077	4.56×10^{-5}	1.73×10^{-4}	COL14A1	True	True
ADAMTS16	94.21	0.5875	0.2213	2.654	0.00795	0.01695	ADAMTS16	True	True
AGRN	1171	-0.5853	0.1177	-4.974	6.56×10^{-7}	3.88×10^{-6}	AGRN	True	True
